# Rational ion transport management mediated through membrane structures

**DOI:** 10.1002/EXP.20210101

**Published:** 2021-10-30

**Authors:** Yupeng Chen, Zhongpeng Zhu, Ye Tian, Lei Jiang

**Affiliations:** ^1^ Key Laboratory of Bio‐Inspired Smart Interfacial Science and Technology of Ministry of Education, School of Chemistry Beihang University Beijing P. R. China; ^2^ CAS Key Laboratory of Bio‐Inspired Materials and Interfacial Science CAS Center for Excellence in Nanoscience Technical Institute of Physics and Chemistry, Chinese Academy of Sciences Beijing P. R. China; ^3^ University of Chinese Academy of Sciences Beijing P. R. China; ^4^ School of Future Technology University of Chinese Academy of Sciences Beijing P. R. China

**Keywords:** ion gating, ion rectification, ion selectivity, ion storage, ion transport, membrane structure

## Abstract

Unique membrane structures endow membranes with controlled ion transport properties in both biological and artificial systems, and they have shown broad application prospects from industrial production to biological interfaces. Herein, current advances in nanochannel‐structured membranes for manipulating ion transport are reviewed from the perspective of membrane structures. First, the controllability of ion transport through ion selectivity, ion gating, ion rectification, and ion storage is introduced. Second, nanochannel‐structured membranes are highlighted according to the nanochannel dimensions, including single‐dimensional nanochannels (i.e., 1D, 2D, and 3D) functioning by the controllable geometrical parameters of 1D nanochannels, the adjustable interlayer spacing of 2D nanochannels, and the interconnected ion diffusion pathways of 3D nanochannels, and mixed‐dimensional nanochannels (i.e., 1D/1D, 1D/2D, 1D/3D, 2D/2D, 2D/3D, and 3D/3D) tuned through asymmetric factors (e.g., components, geometric parameters, and interface properties). Then, ultrathin membranes with short ion transport distances and sandwich‐like membranes with more delicate nanochannels and combination structures are reviewed, and stimulus‐responsive nanochannels are discussed. Construction methods for nanochannel‐structured membranes are briefly introduced, and a variety of applications of these membranes are summarized. Finally, future perspectives to developing nanochannel‐structured membranes with unique structures (e.g., combinations of external macro/micro/nanostructures and the internal nanochannel arrangement) for mediating ion transport are presented.

## Graphical abstract

Unique membrane structures endow membranes with controlled ion transport properties in both biological and artificial systems, showing broad application prospects in different fields. In this review, current advances in nanochannel‐structured membranes for manipulating ion transport are reviewed from the perspective of membrane structures, followed by a discussion of stimulus responsiveness, construction methods, and applications. Finally, future perspectives for developing membranes with unique structures for mediating ion transport are presented.

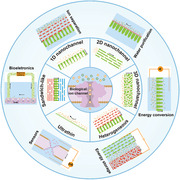

## 1 INTRODUCTION

Ion transport plays a significant role in biological activities, ranging from the basic uptake of ionic mineral elements to the high‐level conversion of biological energy and sensitive transmission of nerve signals. Recently, great efforts have been made to explore the protein structures and underlying ion transport mechanisms of biological ion channel proteins, including cations (e.g., ATPase proton pump,^[^ ^1^ ^]^ TrkH potassium‐ion channel,^[^ ^2^ ^]^ rhodopsin sodium‐ion pump,^[^ ^3^ ^]^ CorA magnesium ion channel,^[^ ^4^ ^]^ TRPV6 calcium ion channel,^[^ ^5^ ^]^ and sCtr1 copper ion transporter^[^ ^6^ ^]^) and anions (e.g., CLC chloride ion channel^[^ ^7^ ^]^ and FNT3 as a hydrosulfide ion channel^[^ ^8^ ^]^). For instance, the opening and closing of the human epithelial calcium channel TRPV6 occurs by exposing different residues to the ion channel caused by the α‐ to π‐helical transition.^[^ ^5^ ^]^ Ion channel proteins are also stimulus responsive to light,^[^ ^9^ ^]^ voltage,^[^ ^10^ ^]^ mechanics,^[^ ^11^ ^]^ ions,^[^ ^12^ ^]^ and molecules.^[^ ^13^ ^]^ These studies have not only deepened our understanding of biological ion channel proteins but also provided inspiration for the development of novel artificial nanochannel‐structured membranes for transporting specific ions. For example, reconstituted ion channels can be directly obtained after inserting extracted biological ion channel proteins^[^ ^14^, ^15^, ^16^, ^17^, ^18^, ^19^, ^20^, ^21^ ^]^ or artificial components with channels^[^ ^22^, ^23^ ^]^ in phospholipid bilayers. Reconstructed EcClC proteins embedded in lipid bilayers can function as H^+^ and Cl^−^ pumps with improved and controllable transport properties because of the superimposing or counterimposing gradients of H^+^ and Cl^−^ ions.^[^ ^14^ ^]^ However, this type of reconstituted ion channel usually requires sophisticated technology, limiting further applications.

**FIGURE 1:**
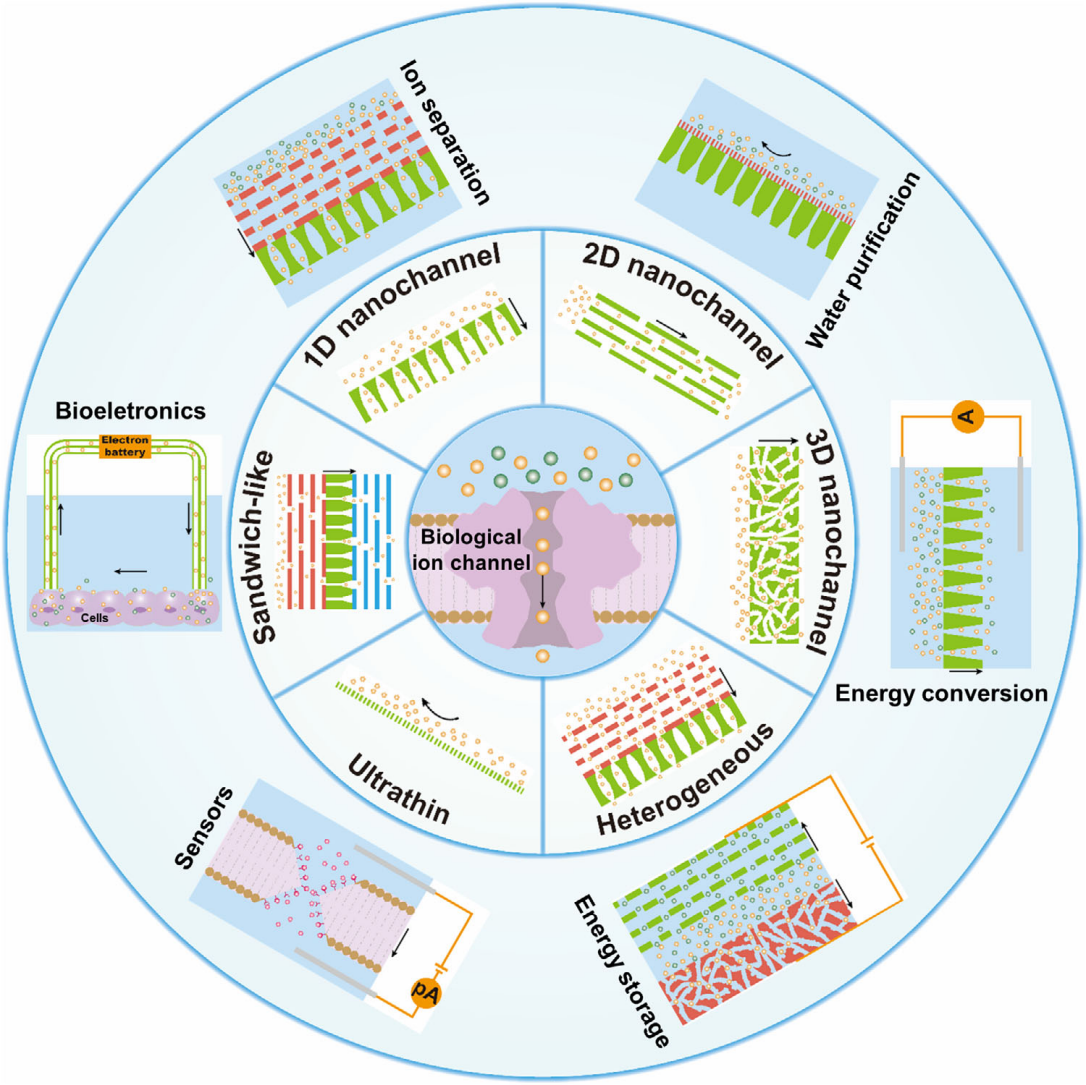
Representative membrane structures for rational ion transport management, including nanochannel‐structured membranes with single‐dimensional nanochannels (i.e., 1D, 2D, and 3D) and mixed‐dimensional nanochannels (i.e., 1D/1D, 1D/2D, 1D/3D, 2D/2D, 2D/3D, and 3D/3D), ultrathin membranes, and sandwich‐like membranes, and their broad applications in the fields of ion separation, water purification, energy storage and conversion, sensors, and bioelectronics

At present, research on nanochannel‐structured membranes for ion transport is mainly focused on selecting artificial raw materials (i.e., components) and constructing nanochannels with specific geometric parameters (e.g., shape and size) and interface properties (e.g., charge, wettability, and recognition). Typical examples of nanochannel‐structured membranes with single‐dimensional nanochannels (i.e., 1D, 2D, and 3D) are provided according to the nanochannel dimensions. For instance, the threading/dethreading states between positively charged azobenzene and negatively charged pillararene on conical nanochannel walls made of polyethylene terephthalate (PET) can be reversibly switched under light irradiation, which contributes to the change in surface charge and achieves ion‐selective transport.^[^ ^24^ ^]^ As a representative reconstructed layered material, negatively charged graphene oxide (GO) with 2D nanochannels 1 nm high showed surface‐charge‐controlled ion transport at high salt concentrations, exhibiting the potential for developing flexible and large‐scale nanochannel‐structured membranes.^[^ ^25^ ^]^ A metal–organic framework (MOF) is a typical porous crystalline material with uniform pores. In situ‐grown UiO‐66‐X MOFs in PET nanochannels showed ultrahigh F^−^ conductivity and F^−^/Cl^−^ selectivity owing to the specific interactions between F^−^ and F^−^ binding sites in channels consisting of angstrom‐scale windows and nanoscale cavities.^[^ ^26^ ^]^ With the development of nanochannel‐structured membranes, a diversity of membrane structures can be constructed, such as nanochannel‐structured (e.g., single‐ and mixed‐dimensional nanochannels), ultrathin (e.g., sub‐micrometer and monolayer), and sandwich‐like (e.g., three‐layer and multilayer) membranes (Figure 1). 1D nanochannels, which are typically simplified nanochannel models, are widely used for transporting specific ions, taking advantage of suitable raw materials, controllable geometrical parameters, and selective surface modifications.^[^ ^27^, ^28^ ^]^ By adjusting the interlayer spacing by physical or chemical methods, membranes with 2D nanochannels consisting of restacked nanosheets showed controlled ion transport properties.^[^ ^29^, ^30^, ^31^ ^]^ However, continuous 3D nanochannels can shorten the ion diffusion pathways and facilitate ion transport.^[^ ^32^ ^]^ Typical heterogeneous membranes with mixed‐dimensional nanochannels (i.e., 1D/1D, 1D/2D, 1D/3D, 2D/2D, 2D/3D, and 3D/3D) generally exhibit unique ion transport properties in contrast to the intrinsic ones because of their asymmetric characteristics such as asymmetric components, geometric parameters (e.g., shape and size), and interface properties (e.g., charge and wettability).^[^ ^33^, ^34^ ^]^ Recently, ultrathin membranes with vertically ordered channels were constructed that showed both high permeability and selectivity owing to the short ion transport distance.^[^ ^35^, ^36^ ^]^ Furthermore, sandwich‐like membranes with a more delicate design of nanochannels and combination structures were applied to precisely tune the ion transport behaviors.^[^ ^37^, ^38^ ^]^ Notably, combining the external macro/micro/nanostructure and the internal nanochannel arrangement adjustment, traditional membranes are endowed with peculiar structures (e.g., anti‐T^[^ ^39^ ^]^ and vertically aligned^[^ ^40^ ^]^ MXenes) and ion transport properties (e.g., directional ion transport). In addition, natural plants (e.g., grass stems^[^ ^41^, ^42^ ^]^ and woods^[^ ^43^, ^44^, ^45^, ^46^ ^]^) with abundant ion channels have also been used to manipulate ion transport after further chemical treatments. Thus, rational ion transport management can be achieved by mediating the membrane structures.

The controllability of ion transport in nanochannel‐structured membranes is mainly reflected in the different ion transport properties (e.g., ion selectivity, ion gating, ion rectification, and ion storage). With respect to construction methods, superwettable interface‐based reactions and assemblies^[^ ^47^, ^48^ ^]^ show great potential for fabricating ultrathin and mixed‐dimensional membranes. For instance, ultrathin membranes have been fabricated by superspreading liquids on superhydrophilic surfaces.^[^ ^49^, ^50^, ^51^ ^]^ Membranes with microscale lateral lengths were also developed through triphase interface‐mediated epitaxial growth on superhydrophobic surfaces,^[^ ^52^, ^53^ ^]^ exhibiting the potential for constructing mixed‐dimensional membranes. Significantly, the applications of nanochannel‐structured membranes are gradually changing from general industrial production to bioelectronics (e.g., electron batteries for detecting ionic signals in biosystems^[^ ^41^ ^]^). Very recently, Jiang et al. reported a quantum‐confined ion superfluid related to biosystems, which shows a new path to investigating controlled ion transport across the nanochannels in both biological membranes and artificial counterparts.^[^ ^54^, ^55^, ^56^, ^57^ ^]^ Therefore, it is necessary to systematically review recent advances in nanochannel‐structured membranes with unique structures for controlling ion transport to further promote their application in the fields of ion separation, water purification, energy storage and conversion, sensors, and bioelectronics.

**FIGURE 2:**
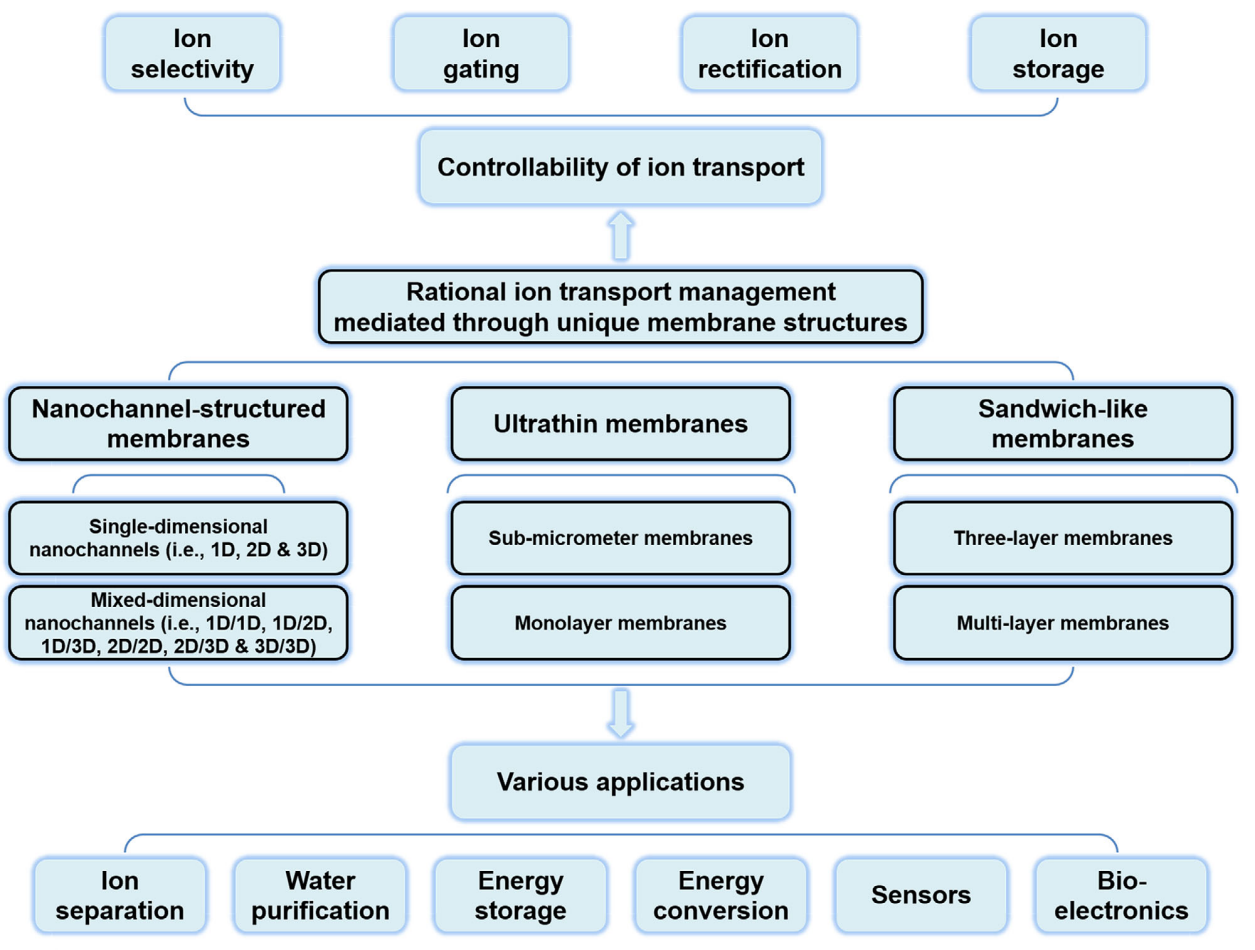
Schematic of rational ion transport management mediated through unique membrane structures. Taking advantage of nanochannel‐structured, ultrathin, and sandwich‐like membranes, ion transport can be manipulated in terms of ion selectivity, ion gating, ion rectification and ion storage, thus showing promising applications in various fields

Herein, we provide a comprehensive overview of rational ion transport management mediated through unique membrane structures (e.g., nanochannel, ultrathin, and sandwich‐like structures) (Figure 2). First, we introduce the controllability of ion transport through ion selectivity, ion gating, ion rectification, and ion storage. Second, we highlight membranes with nanochannels of different dimensions and their heterogeneous membranes. Third, we discuss representative ultrathin membranes and sandwich‐like membranes. Then, various stimulus‐responsive nanochannels are highlighted. Next, we briefly discuss typical construction methods of nanochannel‐structured membranes, followed by a summary of various applications in different fields. Finally, future perspectives in constructing nanochannel‐structured membranes with specific structures for mediating ion transport are presented.

## 2 CONTROLLABILITY OF ION TRANSPORT

To clarify the significant role of the unique structures of nanochannel‐structured membranes for regulating ion transport, it is necessary to first introduce ion transport properties. In this section, the controllability of ion transport in nanochannel‐structured membranes is briefly discussed in terms of ion selectivity, ion gating, ion rectification, and ion storage.

### 2.1 Ion selectivity

Specific ions can be transported across the nanochannels by adjusting the nanochannel size, charge, and wettability of the nanochannel walls and by specific recognition between target ions and recognition sites. More specifically, ions with sizes smaller than the nanochannel diameters can pass through the nanochannels based on the size effect.^[^ ^58^, ^59^, ^60^, ^61^, ^62^, ^63^ ^]^ When the nanochannel diameters are similar to the Debye length, the strong electrostatic interactions between the ions and the nanochannel interfaces can lead to anion‐selective transport in positively charged nanochannels and cation‐selective transport in negatively charged ones.^[^ ^27^, ^64^, ^65^ ^]^ Hydrophilic and hydrophobic nanochannels tend to transport polar and nonpolar matter, respectively.^[^ ^66^, ^67^, ^68^ ^]^ Taking advantage of the specific recognition between the target ions and molecules with binding sites on the inner walls, selective transport of specific cations^[^ ^69^, ^70^ ^]^ and anions^[^ ^71^, ^72^, ^73^ ^]^ can be achieved. The performance of ion selectivity can be quantitatively reflected by the water flux, interception, and ionic conductance of these membranes.

### 2.2 Ion gating

Owing to the changes in the nanochannel size, charge, and wettability of the nanochannel walls, nanochannels usually exhibit stimulus‐responsive closing and opening states. Specific ions can then be conducted or intercepted periodically by nanochannels.^[^ ^27^, ^28^, ^74^ ^]^ For instance, a series of single (e.g., pH,^[^ ^75^ ^]^ light,^[^ ^76^, ^77^ ^]^ electricity,^[^ ^78^ ^]^ and magnetism^[^ ^79^ ^]^) and double (e.g., ion and pH,^[^ ^80^ ^]^ light and pH,^[^ ^81^ ^]^ and electricity and pH^[^ ^82^ ^]^) stimulus‐responsive gatings were obtained after decorating stimulus‐responsive molecules on the nanochannel walls. The ionic conductance of these membranes can be directly used to illustrate the closing and opening states of ion gating.

### 2.3 Ion rectification

Under the effects of the asymmetric shape,^[^ ^83^, ^84^ ^]^ charge,^[^ ^85^ ^]^ and the components^[^ ^67^ ^]^ of nanochannels or asymmetric external stimuli (e.g., pH,^[^ ^86^, ^87^ ^]^ pressure,^[^ ^88^ ^]^ concentration,^[^ ^89^, ^90^ ^]^ and temperature gradients^[^ ^91^ ^]^), specific ions show preferential transport in one direction at a bias, whereas they are blocked in the opposite direction at an inverse bias. For example, negatively charged conical nanochannels exhibit ion rectification owing to their asymmetric structure. When the charge on the nanochannel walls was converted to positive, the ion rectification direction was inverted.^[^ ^65^ ^]^ The performance of ion rectification can be visually illustrated by asymmetric *I*–*V* curves and quantitatively compared using the rectification ratio.

### 2.4 Ion storage

3D interconnected nanochannels can facilitate inserting and extracting electrolyte ions from electrode materials, which is usually adopted in designing electrochemical energy storage devices with superior performance (e.g., ultrahigh energy and power density, suitable cycling stability). For example, electrodes made of holey 2D nanosheets can shorten the ion transport pathways and increase the rate of ion transport, taking advantage of the ultrathin thickness and continuous 3D nanochannels.^[^ ^92^ ^]^ Recently, they have been widely used to construct electrochemical capacitors, lithium‐ion batteries, and sodium‐ion batteries. The corresponding electrochemical performance can be accurately analyzed using cyclic voltammetry (CV) curves, charge/discharge (CD) curves, and specific capacitances.

As mentioned above, the controllability of ion transport is mainly accomplished through ion selectivity, ion gating, ion rectification, and ion storage, which are in turn determined by internal factors such as the components, geometric parameters (e.g., shape and size), and interface properties (e.g., charge and wettability) of these membranes and external factors such as the pH, pressure, concentration, and temperature of the electrolyte solutions. Controllable ion transport mediated by the unique membrane structures (e.g., nanochannel, ultrathin, and sandwich‐like structures) is discussed in the following sections.

## 3 NANOCHANNEL‐STRUCTURED MEMBRANES

**FIGURE 3:**
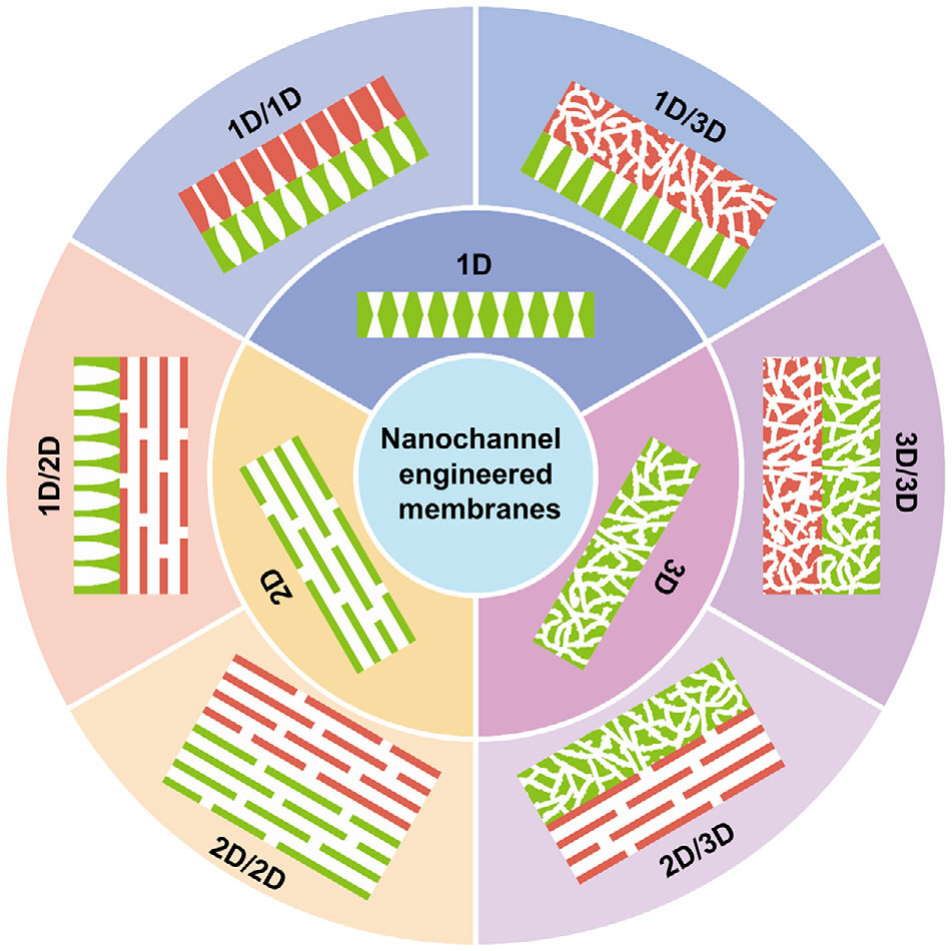
Nanochannel‐structured membranes with single‐dimensional nanochannels (i.e., 1D, 2D, and 3D) and mixed‐dimensional nanochannels (i.e., 1D/1D, 1D/2D, 1D/3D, 2D/2D, 2D/3D, and 3D/3D)

To clarify the concept of nanochannel‐structured membranes with different dimensions (Figure 3), these different dimensions are defined as follows: 1D nanochannel‐structured membranes are membranes with a single nanochannel or nanochannel array in one direction. 2D nanochannel‐structured membranes are membranes with nanoscale interlayer spacing formed by stacking nanosheets. 3D nanochannel‐structured membranes mainly include porous membranes with interconnected 3D nanochannels. In this section, nanochannel‐structured membranes for ion transport are introduced according to the nanochannel dimensions, including both single‐dimensional (i.e., 1D, 2D, and 3D) and mixed‐dimensional (i.e., 1D/1D, 1D/2D, 1D/3D, 2D/2D, 2D/3D, and 3D/3D) nanochannels.

### 3.1 1D Nanochannel‐structured membranes

**FIGURE 4:**
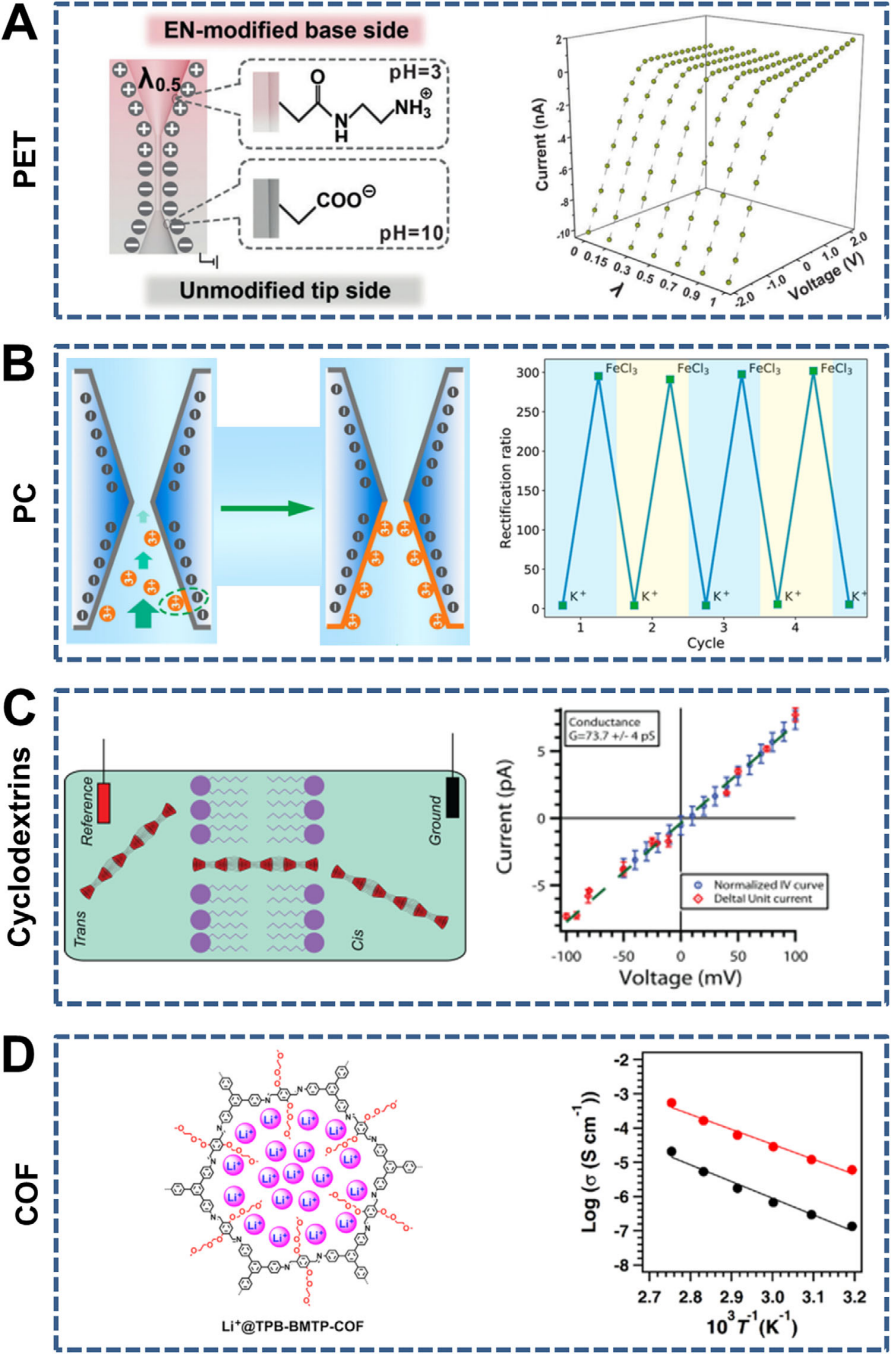
Representative 1D nanochannel‐structured organic membranes. (A) Asymmetric funnel‐shaped PET (left) and *I*–*V* curves with different symmetric geometry degree λ values (right). Reproduced with permission.^[^ ^85^ ^]^ Copyright 2017, Wiley‐VCH. (B) Symmetric hourglass‐shaped polycarbonate (PC) (left) and reversible switching of ion current rectification (ICR) ratios (right). Reproduced with permission.^[^ ^64^ ^]^ Copyright 2019, American Chemical Society. (C) Short cyclodextrins nanotubes (CDNTs) (left) and *I*–*V* curve of the CDNTs (right). Reproduced with permission.^[^ ^108^ ^]^ Copyright 2015, American Chemical Society. (D) Covalent organic framework (COF) with ordered 1D nanochannels (left) and ion conductivities of COFs with (red) and without (black) oligo(ethylene oxide) chains as a function of temperature (right). Reproduced with permission.^[^ ^109^ ^]^ Copyright 2018, American Chemical Society

**FIGURE 5:**
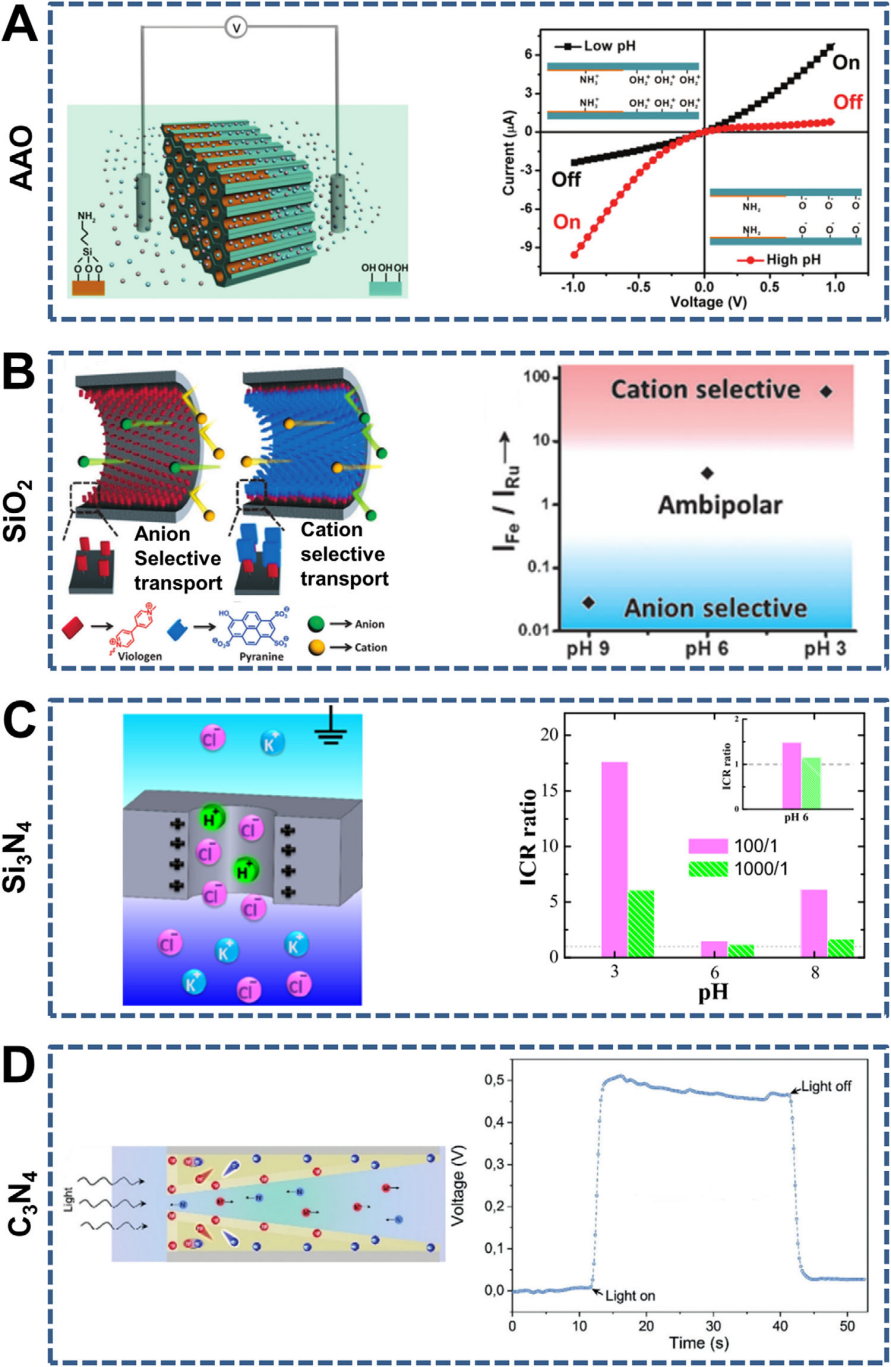
Representative 1D nanochannel‐structured inorganic membranes. (A) Symmetric cylinder‐shaped anodic aluminum oxide (AAO) (left) and pH‐modulated ion rectification property (right). Reproduced with permission.^[^ ^110^ ^]^ Copyright 2013, Wiley‐VCH. (B) Non‐covalent functionalization of ordered and perpendicular mesochannels of silica (SiO_2_) (left) and the distinct ion transport regions of anion selective, ambipolar, and cation selective (right). Reproduced with permission.^[^ ^116^ ^]^ Copyright 2014, Wiley‐VCH. (C) Silicon nitride (Si_3_N_4_) nanopore (left) and ICR ratios at different pH and salt gradient values (right). Reproduced with permission.^[^ ^90^ ^]^ Copyright 2019, American Chemical Society. (D) Asymmetric cone‐shaped carbon nitride (C_3_N_4_) nanotubes (left) and photocurrent responses with tip‐side irradiation (right). Reproduced with permission.^[^ ^124^ ^]^ Copyright 2019, Wiley‐VCH

To mimic biological ion channels embedded in plasma membranes to finely modulate ion transport, constructing artificial counterparts has been carried out in many studies. As a representative simplified nanochannel model, 1D nanochannels with various raw materials, controllable geometrical parameters, and selective surface modifications have been widely used for studying tunable ion transport. Here, we introduce 1D nanochannel‐structured membranes for ion transport based on their composition (e.g., organic and inorganic membranes) (Figures 4 and 5, respectively).

First, representative 1D nanochannel‐structured organic membranes with various geometrical parameters include PET, polyimide (PI), polycarbonate (PC), short cyclodextrin nanotubes (CDNTs), and covalent organic frameworks (COFs) are discussed. PET is usually used to prepare symmetric (e.g., cylindrical^[^ ^93^ ^]^ and cigar‐shaped^[^ ^94^, ^95^, ^96^ ^]^) and asymmetric (e.g., bullet‐,^[^ ^83^ ^]^ funnel‐,^[^ ^85^, ^97^ ^]^ hourglass‐,^[^ ^75^, ^98^ ^]^ and cone‐shaped^[^ ^24^, ^79^, ^99^, ^100^, ^101^, ^102^, ^103^ ^]^) nanochannels. For instance, PET membranes with cylindrical nanochannels can provide pathways for proton transport across a transmembrane concentration gradient. In one study, these membranes exhibited a continuous and stable current and were used for constructing respiration‐based biocells with superior performance.^[^ ^93^ ^]^ By fine adjustment of the symmetric geometry degree λ (i.e., the ratio of the tip‐side conical length to the base‐side conical length) and the surface charge on the inner walls, the funnel‐shaped nanochannels showed switchable ion transport properties between bidirectional rectification and ion gating (Figure 4A).^[^ ^85^ ^]^ In addition, Siwy et al. constructed an asymmetric cone‐shaped PI nanochannel with a large opening approximately 2.4 μm in diameter and an estimated small opening approximately 2 nm in diameter by a track‐etching method.^[^ ^104^ ^]^ The prepared nanochannel stably rectified the ion current owing to the asymmetric charge distribution at the nanochannel interface. In addition, asymmetric (e.g., cone‐^[^ ^105^, ^106^ ^]^ and hourglass‐shaped^[^ ^64^ ^]^) PC nanochannels showed stimulus‐responsive ion rectification owing to the change in surface charge. Taking advantage of the adsorption of multivalent ions (e.g., trivalent iron ions) on negatively charged hourglass‐shaped nanochannels with a 208 nm base diameter and a 0.91 nm tip diameter, the corresponding local charge was inverted and a bipolar junction was formed, leading to an ion current rectification (ICR) ratio higher than 650 (Figure 4B).^[^ ^64^ ^]^ Furthermore, the ICR ratio could be reversibly tuned between high and low through the adsorption and desorption of multivalent ions. CDNTs with controlled lengths, diameters, and number of cyclodextrins and selective chemical modifications were used as ion nanochannels and inserted into lipid bilayers to transport ions.^[^ ^107^, ^108^ ^]^ As shown by the *I*–*V* curves of the CDNTs (Figure 4C), their conductance was found to be 0.077 ± 0.005 nS.^[^ ^108^ ^]^ In addition, a COF with oligo(ethylene oxide) chains exhibited enhanced lithium‐ion transport through a vehicle mechanism in contrast to a COF without oligo(ethylene oxide) chains on the ordered 1D nanochannel walls (Figure 4D),^[^ ^109^ ^]^ which provides new inspiration for developing ion conductors.

Many inorganic materials such as anodic aluminum oxide (AAO), carbon nanotubes (CNTs), silica (SiO_2_), silicon nitride (Si_3_N_4_), and carbon nitride (C_3_N_4_) have been used to construct 1D nanochannel‐structured membranes. Common symmetric (i.e., cylinder‐shaped^[^ ^110^, ^111^ ^]^) and asymmetric (e.g., cone‐^[^ ^112^ ^]^ and hourglass‐shaped^[^ ^113^ ^]^) nanochannels can be fabricated by AAO. For instance, a cylindrical nanochannel‐structured AAO decorated with 3‐aminopropyltrimethoxy‐silane (APTMS) at the desired positions was constructed using a two‐step anodization method (Figure 5A).^[^ ^110^ ^]^ Because of the asymmetric charge distribution induced by the protonation or deprotonation of the modified amine and the intrinsic hydroxyl groups on the inner walls, the prepared AAO nanochannels exhibited pH‐modulated ion rectification. Inspired by the structure of biological ion channel proteins (i.e., KcsA), researchers designed a single‐walled CNT decorated with carbonyl oxygen atoms in a special arrangement along the inner wall for the selective separation of Na^+^ and K^+^ ions.^[^ ^114^ ^]^ The simulation results showed that the hydration structure of the ions in the confined nanochannel led to a remarkable sieving ability, which could be finely adjusted by changing the positions of the carbonyl oxygen atoms. Furthermore, various nanochannel structures have been found in SiO_2_ membranes, including conical^[^ ^115^ ^]^ and cylindrical (i.e., SBA‐15^[^ ^116^, ^117^, ^118^ ^]^) nanochannels and mesochannels with 3D cubic structures (i.e., SBA‐16^[^ ^119^, ^120^ ^]^). For instance, by adjusting the extent of positively charged acceptors (e.g., viologen) bound to a negatively charged donor (e.g., pyranine), the charge in the confined mesochannels (<10 nm) of silica films will invert, leading to controllable ion transport from anion‐selective to ambipolar to cation‐selective (Figure 5B).^[^ ^116^ ^]^ Moreover, the cylindrical nanochannel‐structured mesoporous silica showed surface charge‐governed proton transport at low proton concentrations.^[^ ^118^ ^]^ When a lower gate voltage (1 V) was applied to the membranes, the corresponding proton conduction was enhanced two‐ to fourfold. For example, the surface charge of cylindrical Si_3_N_4_ nanopores with a diameter of 10 nm and a length of 30 nm could be modulated through both the applied voltage and the salt gradient under a constant pH value, showing great application potential in physiological research (Figure 5C).^[^ ^90^ ^]^ In addition, the ion transport properties of cylindrical^[^ ^121^, ^122^, ^123^ ^]^ and conical^[^ ^124^ ^]^ C_3_N_4_ nanotubes were also investigated. For example, asymmetric cone‐shaped C_3_N_4_ nanotubes with a 70–80 nm base diameter and a 15–20 nm tip diameter were used to fabricate highly sensitive and stable ionic photodetectors, where the surface charge gradient along the nanochannels induced by unilateral illumination led to controlled ion transport (Figure 5D).^[^ ^124^ ^]^

According to the material composition, 1D nanochannel‐structured membranes with ion transport properties are mainly classified as either organic (e.g., PET, PI, PC, CDNTs, and COF) or inorganic (e.g., AAO, CNTs, SiO_2_, Si_3_N_4_, C_3_N_4_). Controlled ion transport is achieved through the precise mediation of geometrical parameters and selective surface modifications at specific positions. However, the raw materials used to construct 1D nanochannels are still limited. Very recently, Hu et al. performed excellent work on ionic conductive wood with aligned nanochannels. Positively charged artificial wood (i.e., cationic wood) consisting of cellulose nanofibers was used to transport ions through the aligned nanochannels between these nanofibers, which not only exhibited excellent mechanical properties (5.5‐ and 20‐times improvement in wet and dry conditions, respectively) but also dramatically enhanced ion conductance (25‐times improvement at a low KCl concentration).^[^ ^44^ ^]^ Furthermore, it is meaningful to achieve synchronous enhancement of the ability to transport ions from a single nanochannel to multiple nanochannels.^[^ ^125^, ^126^ ^]^ Thus, it is necessary to seek new materials to design 1D nanochannels with fine geometrical parameters (e.g., diameter, length, curvature, and density) and surface modifications and to promote their application in ion transport‐related areas.

### 3.2 2D Nanochannel‐structured membranes

**FIGURE 6:**
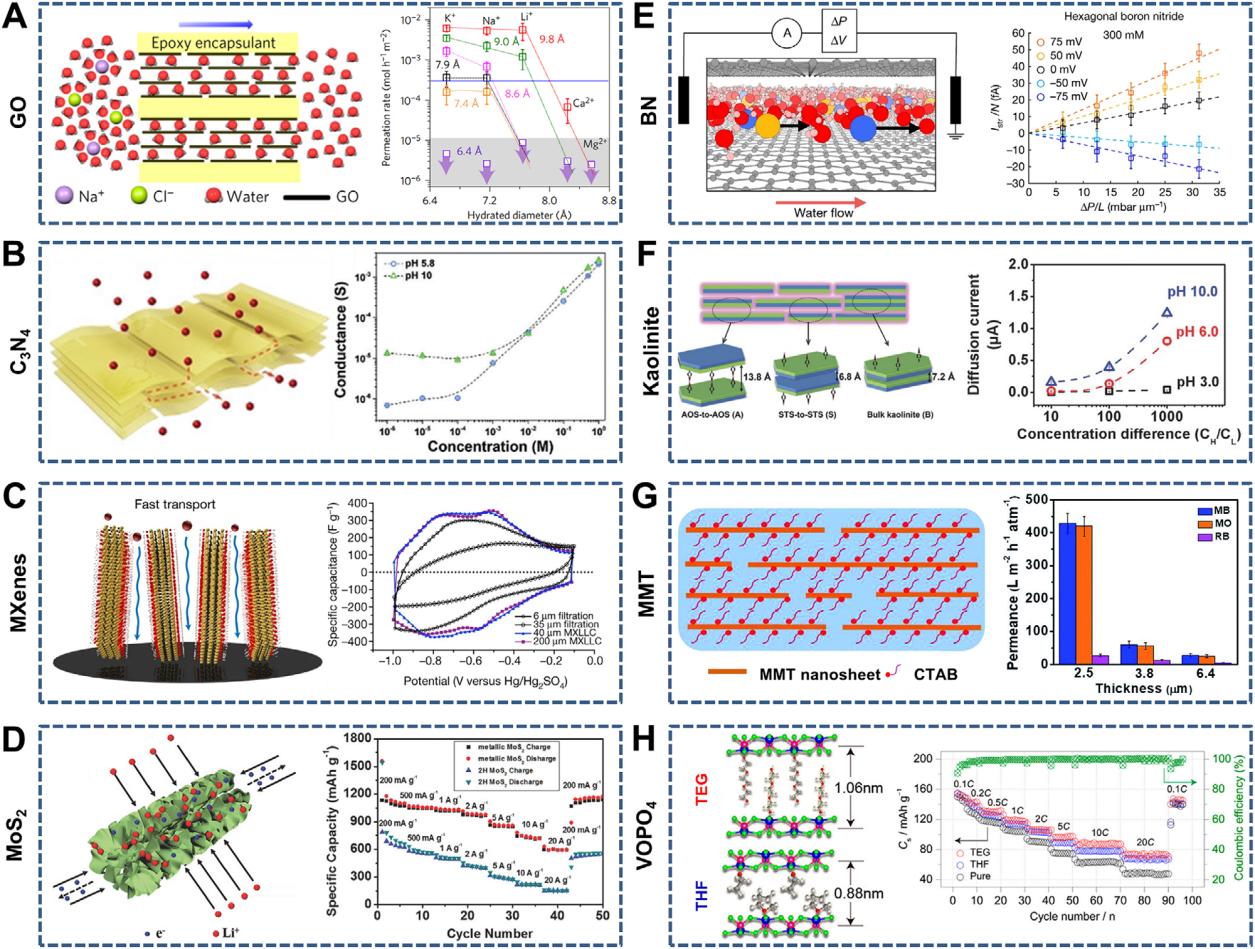
Representative 2D nanochannel‐structured inorganic membranes. (A) Graphene oxide (GO) under physical confinement (left) and permeation rates through GO with adjustable interlayer spacing (right). Reproduced with permission.^[^ ^61^ ^]^ Copyright 2017, Macmillan Publishers Limited. (B) C_3_N_4_ (left) and conductance‐salt concentration curves at different pH values (right). Reproduced with permission.^[^ ^134^ ^]^ Copyright 2018, Wiley‐VCH. (C) Vertically aligned MXenes (left) and cyclic voltammograms of MXenes with different arrangement forms (right). Reproduced with permission.^[^ ^40^ ^]^ Copyright 2018, Macmillan Publishers Limited. (D) Vertically aligned MoS_2_ on the AAO inner walls (left) and comparison of the rate performance between metallic MoS_2_ nanotubes and 2H MoS_2_ nanosheets (right). Reproduced with permission.^[^ ^142^ ^]^ Copyright 2018, Wiley‐VCH. (E) Boron nitride (BN) with angstrom‐scale channels (left) and pressure‐driven streaming current under various voltage. (Δ*P* and Δ*V* represent pressure and voltage, respectively) (right). Reproduced with permission.^[^ ^143^ ^]^ Copyright 2019, Macmillan Publishers Limited. (F) Kaolinite (left) and diffusion current–concentration difference curves at different pH values (right). Reproduced with permission.^[^ ^144^ ^]^ Copyright 2017, Wiley‐VCH. (G) MMT (left) and permeance toward to methylene blue (MB), methyl orange (MO), and rhodamine B (RB) (right). Reproduced with permission.^[^ ^146^ ^]^ Copyright 2019, The Royal Society of Chemistry. (H) Vanadyl phosphate (VOPO_4_) intercalated with triethylene glycol (TEG) and tetrahydrofuran (THF) (left) and rate performance of the pure, TEG‐, and THF‐intercalated VOPO_4_ (right). Reproduced with permission.^[^ ^147^ ^]^ Copyright 2017, American Chemical Society

2D nanochannel‐structured membranes with nanoscale interlayer spacing are mainly composed of inorganic nanosheets^[^ ^30^, ^127^ ^]^ such as GO, C_3_N_4_, MXenes, MoS_2_, boron nitride (BN), kaolinite, montmorillonite (MMT), and vanadyl phosphate (VOPO_4_). These membranes show controlled ion transport properties by adjusting the interlayer spacing between the restacked nanosheets by physical or chemical methods. Here, representative 2D nanochannel‐structured inorganic membranes made of various nanosheets are discussed (Figure 6).

Typical 2D nanochannel‐structured inorganic membranes made of various nanosheets (e.g., GO, C_3_N_4_, MXenes, MoS_2_, BN, kaolinite, MMT, and VOPO_4_) are presented as follows. Since the fabrication of 2D nanofluidic channels based on restacked GO nanosheets have been reported, many studies have focused on the use of 2D nanochannel‐structured membranes for controllable ion transport. Taking GO membranes as an example, both chemical (e.g., cross‐linking with intercalated molecules^[^ ^128^, ^129^, ^130^ ^]^) and physical (e.g., physical confinement^[^ ^61^, ^131^, ^132^ ^]^ and ion intercalation^[^ ^133^ ^]^) methods have been applied to mediate the interlayer spacing. When layered GO membranes are physically embedded in epoxy, their swelling is mechanically restricted (Figure 6A).^[^ ^61^ ^]^ Then, the interlayer spacing *d* can be finely tuned between 6.4 and 9.8 Å, exhibiting a smaller channel size than that of hydrated ions. Through this strategy, accurate ion sieving based on size selection can be achieved. Multilayered C_3_N_4_ membranes with controlled thicknesses from 140 nm to 1 μm showed surface‐charge‐controlled ion transport (Figure 6B).^[^ ^134^ ^]^ Anion intercalation can also be used to tune the interlayer spacing for selective permeation.^[^ ^135^ ^]^ For MXenes, both the interlayer spacing and the arrangement structure (e.g., vertically aligned and anti‐T MXenes) were controlled through chemical^[^ ^136^ ^]^ and physical^[^ ^39^, ^40^, ^137^, ^138^ ^]^ methods to investigate their ion transport properties. For instance, vertically aligned 2D titanium carbide (Ti_3_C_2_T*_x_*) MXene membranes enabled directional ion transport through fast transport pathways, which can be used as thickness‐independent capacitance for electrochemical energy storage (Figure 6C).^[^ ^40^ ^]^ This type of membrane also has great application potential in the fields of separation and catalysis. In addition, a typical layered Ti_3_C_2_ MXene membrane could be bent owing to its mechanical flexibility while maintaining the surface charge‐governed ion transport characteristics.^[^ ^139^ ^]^ MoS_2_ with expanded interlayer spacing provided a high‐efficiency electron/ion transport pathway for rapid Li^+^ and K^+^ transport.^[^ ^140^, ^141^ ^]^ Recently, Zhu et al. prepared vertically aligned metallic MoS_2_ on AAO inner walls (i.e., metallic MoS_2_ nanotubes) using a solvothermal method.^[^ ^142^ ^]^ As the electrode material in lithium‐ion batteries, the metallic MoS_2_ nanotubes exhibited remarkable rate performance (589 mA h g^−1^) at a high current density (20 A g^−1^) because of the fast electrolyte ion transport induced by the porous and aligned structure (Figure 6D). The nanofluidic channels between individual BN nanosheets for ion transport have also been studied in recent years. For instance, BN membranes with angstrom‐scale channels constructed through van der Waals assembly showed adjustable pressure‐driven ion transport under various voltages, which can contribute to understanding the underlying principles of mechanosensitive ion nanochannels in biological and artificial membranes (Figure 6E).^[^ ^143^ ^]^ Furthermore, Jiang et al. reported reconstituted kaolinite membranes with channels with sub‐nanometer (6.8 Å) and nanoscale (13.8 Å) widths, demonstrating obvious cation‐selective transport controlled by the surface charge (Figure 6F).^[^ ^144^ ^]^ Other 2D nanochannel‐structured membranes made of MMT^[^ ^145^, ^146^ ^]^ and VOPO_4_^[^ ^147^ ^]^ nanosheets have also attracted the attention of researchers. For example, lamellar MMT membranes (water permeance of 429 L m^−2^ h^−1^ atm^−1^, thickness of 2.5 μm) decorated with a cationic surfactant showed high separation efficiency for cationic and anionic dyes (Figure 6G).^[^ ^146^ ^]^ VOPO_4_ membranes have expanded interlayer spacing through controlled intercalation of organic molecules such as triethylene glycol (TEG) and tetrahydrofuran (THF), which can be used to improve sodium‐ion storage (Figure 6H).^[^ ^147^ ^]^

Representative 2D nanochannel‐structured inorganic membranes have been introduced based on restacked nanosheets, which include GO, C_3_N_4_, MXenes, MoS_2_, BN, kaolinite, MMT, and VOPO_4_. To clarify the regulation of the 2D nanochannel structure for ion transport, the corresponding strategies (e.g., physical or chemical methods) for adjusting the interlayer spacing and special arrangement of the nanosheets were discussed. It is meaningful to develop 2D nanochannel‐structured membranes using other layered inorganic and organic materials and to investigate the corresponding ion transport characteristics. For example, Hu et al. reported that multiple confined spacings (∼1 nm) consisting of graphite flakes and cellulose nanofibers exhibited higher cationic conductivity owing to abundant nanochannels and a negatively charged surface.^[^ ^148^ ^]^ Layered ionic liquids with nanoconfined nanochannels were constructed through complexation between surfactants and ionic liquids, showing temperature‐mediated ion transport along the parallel and normal directions.^[^ ^149^ ^]^ Recently, 2D nanochannel‐structured membranes with special surface and nanochannel arrangement structures have attracted the attention of researchers. For instance, anti‐T^[^ ^39^ ^]^ MXenes showed ion transport in the vertical direction because of the specific surface structure and vertically aligned^[^ ^40^ ^]^ structure, exhibiting rapid ion transport owing to the efficient ion transport pathways resulting from the arrangement structure. It is still a challenge to further fabricate 2D nanochannel‐structured membranes by mediating the surface and nanochannel arrangement structures, as well as by choosing novel raw materials.

### 3.3 3D Nanochannel‐structured membranes

**FIGURE 7:**
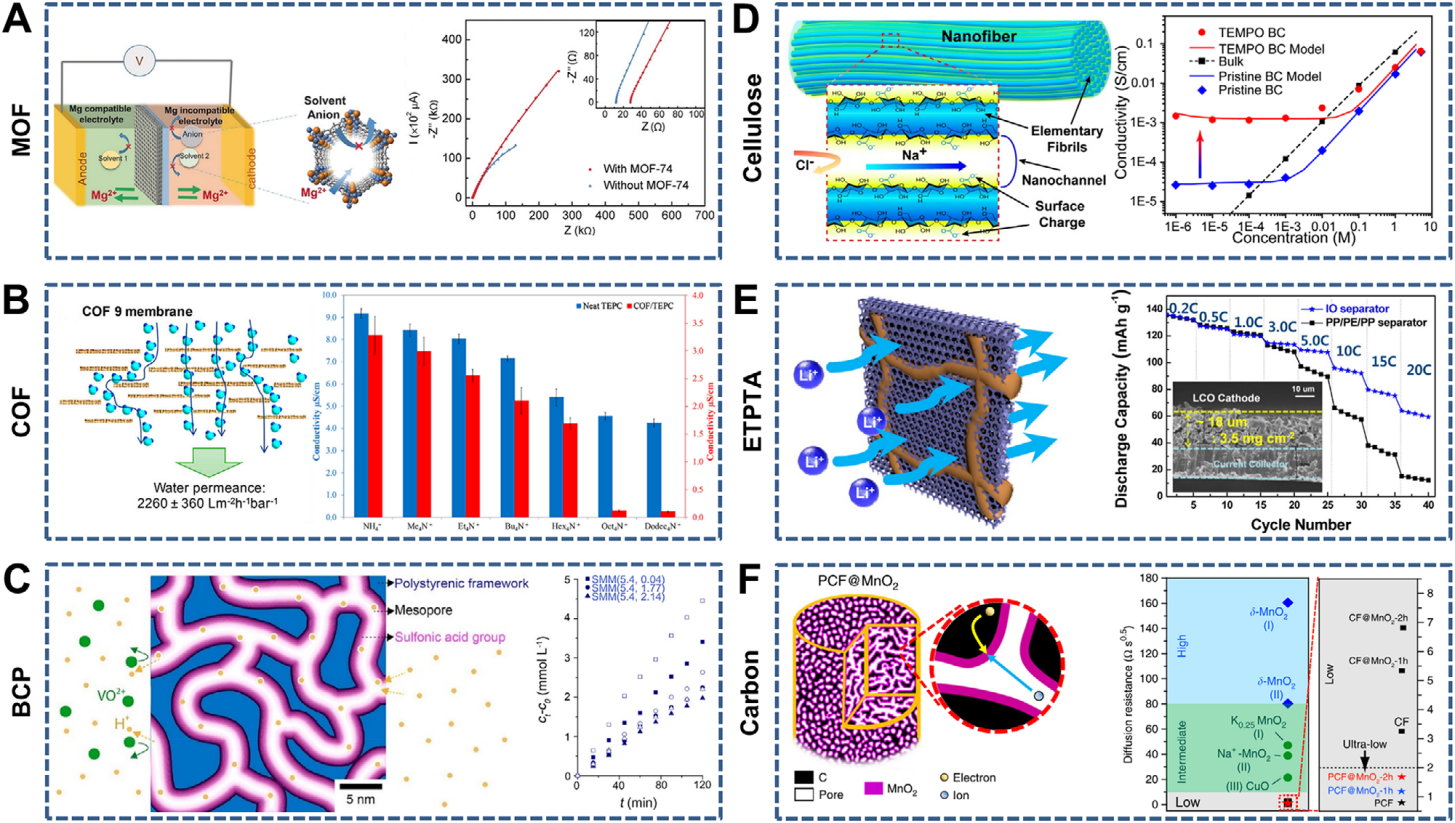
Representative 3D nanochannel‐structured membranes. (A) MOF membranes as the separators (left) and electrochemical impedance spectra obtained with and without MOF on Au‐coated Si wafers (right). Reproduced with permission.^[^ ^150^ ^]^ Copyright 2019, Wiley‐VCH. (B) Layered COF membranes with efficient water transport pathways (left) and cation transport through the membranes (right). Reproduced with permission.^[^ ^58^ ^]^ Copyright 2018, American Chemical Society. (C) Block copolymer (BCP) with continuous pore structure (left) and ion concentration–time curves across the membranes with different sulfonic acid content (right). Reproduced with permission.^[^ ^162^ ^]^ Copyright 2018, American Chemical Society. (D) Interconnected 3D nanochannels consisting of cellulose nanofibers (left) and ionic conductivity of the treated and untreated cellulose nanofibers (right). Reproduced with permission.^[^ ^45^ ^]^ Copyright 2018, American Chemical Society. (E) Ethoxylated trimethylolpropane triacrylate (ETPTA) with 3D inverse opal structure (left) and discharge C‐rate capability (right). Reproduced with permission.^[^ ^163^ ^]^ Copyright 2014, American Chemical Society. (F) Mesoporous carbon fibers (left) and ion diffusion resistance of the prepared carbon fiber electrodes (right). Reproduced under the terms of the Creative Commons CC BY license.^[^ ^166^ ^]^ Copyright 2019, The Author(s)

Representative 3D nanochannel‐structured membranes have been fabricated using various raw materials, including MOFs, COFs, conjugated microporous polymers (CMPs), block copolymers (BCPs), cellulose nanofibers, ethoxylated trimethylolpropane triacrylate (ETPTA), carbon fibers, and SiO_2_ nanoparticles. The common characteristic of these membranes is the continuous 3D nanochannels, which shorten the ion diffusion pathways and facilitate ion transport. Thus, we present the diversity of 3D nanochannel‐structured membranes with respect to the material compositions (Figure 7).

Here, typical 3D nanochannel‐structured membranes (e.g., MOFs, COFs, CMPs, BCPs, cellulose nanofibers, ETPTA, carbon fibers, and SiO_2_ nanoparticles) with tunable ion transport properties are discussed. As typical polycrystalline microporous framework membranes, MOFs commonly exhibit interconnected and well‐defined pores on the angstrom scale. Hence, MOFs are widely used for the selective transport of alkali metal ions and alkali earth metal ions with or without the introduction of polyelectrolytes (e.g., poly(sodium vinyl sulfonated‐*co*‐acrylic acid and polystyrene sulfonate).^[^ ^60^, ^150^, ^151^, ^152^, ^153^, ^154^ ^]^ For instance, uniform Mg‐MOF‐74 membranes were used as the separators for the selective transport of Mg^2+^ while prohibiting the transport of other solvents and counterions (Figure 7A).^[^ ^150^ ^]^ The corresponding ionic conductivity of this kind of MOF membrane with a thickness of 202 nm can reach approximately 3.17 × 10^−6^ S cm^−1^ at room temperature. In addition, MOFs modified with polystyrene sulfonate showed fast and ideal lithium‐ion selectivity owing to the size‐sieving effects and the affinity differences of different ions to the sulfonate groups.^[^ ^154^ ^]^ COF is another representative polycrystalline microporous material. Wang et al. investigated the Li^+^ transport in 3D (i.e., CD‐COF) and 1D (i.e., COF‐5, COF‐300, and EB‐COF) channel‐structured COFs after incorporating them with polyethylene glycol (PEG) in anionic, neutral, and cationic states.^[^ ^155^ ^]^ The results indicated enhanced ion conductivity of 1.78 × 10^−3^ S cm^−1^ at 120 °C in cationic COFs because the local motion of PEG chains in the channels promoted Li^+^ transport. Organic nanosheets (e.g., MOFs and COFs) can also be used for ion separation.^[^ ^31^, ^36^, ^156^, ^157^ ^]^ For example, a nanosheet‐stacked COF 9 membrane with carboxylate‐modified nanopores 2.8 nm in diameter was prepared by Hoberg et al., which exhibited high water flux (∼2260 L m^−2^ h^−1^ bar^−1^) and efficient cation selectivity (Figure 7B).^[^ ^58^ ^]^ CMP is an organic porous polymer that is characterized by both π‐conjugated skeletons and intrinsic nanopores, which can be applied in areas such as ion separation and detection.^[^ ^158^, ^159^, ^160^ ^]^ For example, CMP with carbazole groups (i.e., TPBCz‐CMP) was used to detect redox‐active ions through redox‐induced fluorescence quenching because the carbazole groups in the CMP nanopore walls were inclined to be oxidized.^[^ ^159^ ^]^ In addition, interconnected 3D nanochannels in BCPs can be constructed through the microphase separation method and used to modulate the transport of specific ions.^[^ ^161^, ^162^ ^]^ Seo et al. designed poly(styrene‐*co*‐divinylbenzene) (i.e., P(S‐*co*‐DVB)) membranes with 3D continuous mesopores and obtained controllable ion permeability by mediating the pore size and sulfonic acid content (Figure 7C).^[^ ^162^ ^]^ Very recently, Hu et al. constructed interconnected 3D nanochannels using cellulose nanofibers, which exhibited charge‐adjusted sodium‐ion transport (Figure 7D).^[^ ^45^ ^]^ After further chemical treatment, the cellulose nanofibers showed enhanced zeta potential (−45 mV) and ionic conductivity (1.0 × 10−^3^ S cm^−1^) in contrast to the untreated ones. A new class of battery separators was fabricated using ETPTA with a 3D inverse opal structure, which facilitated rapid ion transport (Figure 7E).^[^ ^163^ ^]^ The special 3D nanochannels endowed the separator with a high discharge C‐rate capability (60 mAh g^−1^ when cathode/anode = 3.5/1.7 mg cm^−2^) compared to common PP/PE/PP separators. Carbon (e.g., mesoporous membranes^[^ ^164^, ^165^ ^]^ and porous fibers^[^ ^166^, ^167^ ^]^) (Figure 7F) and SiO_2_ (e.g., self‐assembled nanoparticles^[^ ^168^, ^169^ ^]^) with 3D nanochannels are common inorganic materials for adjusting ion transport.

**FIGURE 8:**
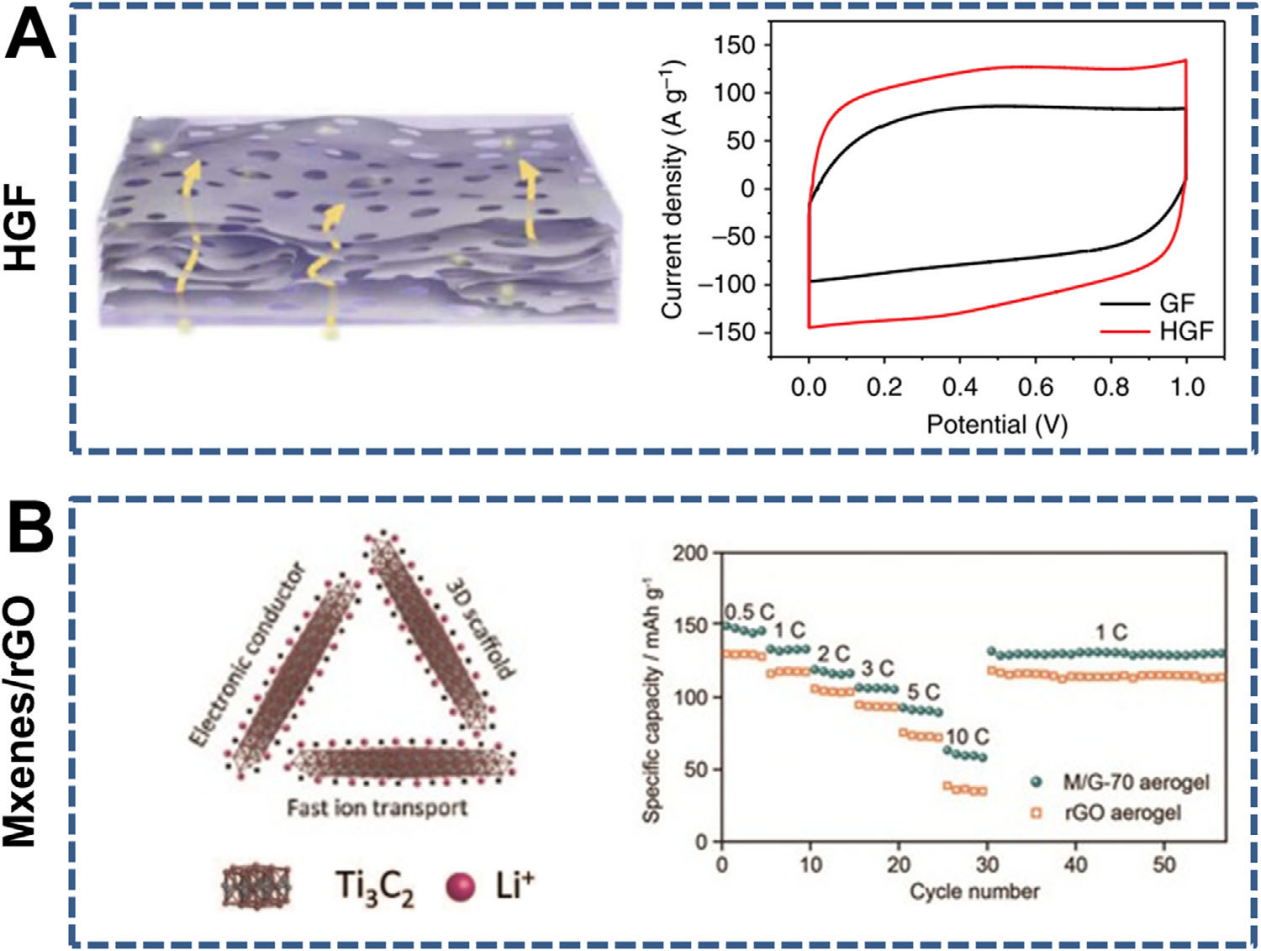
3D nanochannel‐structured membranes consisting of stacked porous nanosheets and hybrid nanosheets. (A) Holey graphene framework (HGF) (left) and CV curves of HGF and GF supercapacitor electrodes (right). Reproduced with permission.^[^ ^171^ ^]^ Copyright 2014, Macmillan Publishers Limited. (B) 3D porous MXene/rGO aerogels (left), rate and cycling performance with MXene/rGO aerogels and rGO aerogels as the anodes (right). Reproduced with permission.^[^ ^176^ ^]^ Copyright 2018, Wiley‐VCH

Inorganic nanosheets, including stacked porous nanosheets (e.g., holey graphene frameworks [HGFs],^[^ ^170^, ^171^ ^]^ porous carbon,^[^ ^172^, ^173^, ^174^ ^]^ and TaS_2_^[^ ^175^ ^]^) and hybrid nanosheets (e.g., MXene/rGO^[^ ^176^, ^177^ ^]^) have also been used to construct 3D nanochannels (Figure 8). Duan et al. designed a high‐performance supercapacitor electrode using 3D HGFs with hierarchical pores (Figure 8A).^[^ ^171^ ^]^ The CV and CD curves exhibited typical rectangular and triangular shapes, indicating efficient ion transport in the electrode. 3D porous MXene/rGO aerogels have good electron conductivity and fast Li^+^ transport capability. As anodes in fuel cells, they showed better rate and cycling performance than the rGO aerogel ones (Figure 8B).^[^ ^176^ ^]^

In this section, typical membranes with interconnected 3D nanochannels were summarized, such as MOFs, COFs, CMPs, BCPs, cellulose nanofibers, ETPTA, carbon fibers, and SiO_2_ nanoparticles. Inorganic nanosheets (e.g., stacked porous nanosheets and hybrid nanosheets) for developing 2D nanochannels can also be used to design 3D nanochannels, which provides inspiration for the novel construction of nanochannel‐structured membranes. Interestingly, natural plants can be used for ion transport after chemical treatment because of their abundant 3D channels. For instance, carbon membranes derived from natural wood were used as electrodes in metal–air batteries, which showed superior specific capacity owing to the hierarchical pores for fast ion transport.^[^ ^178^ ^]^ Therefore, in addition to materials with intrinsic pores, holey or hybrid layered materials and natural plants can also be used to construct 3D porous membranes.

### 3.4 Heterogeneous membranes with mixed‐dimensional nanochannels

**FIGURE 9:**
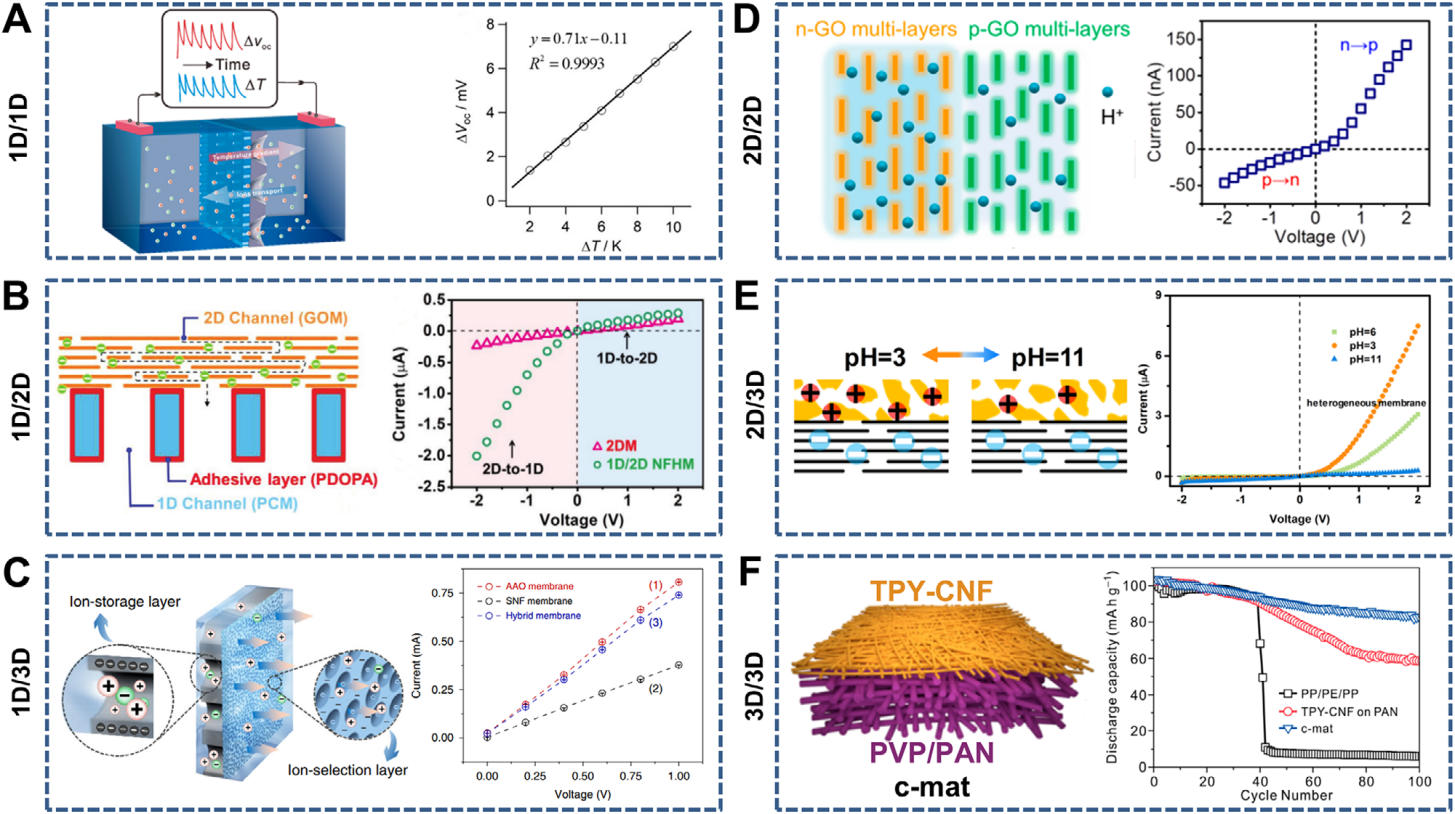
Heterogeneous membranes with mixed‐dimensional nanochannels. (A) 1D/1D heterogeneous membrane consisting of mesoporous SiO_2_ and PET with conical nanochannels (left) and open‐circuit potential Δ*V*_oc_ as a function of temperature difference Δ*T* (right). Reproduced with permission.^[^ ^188^ ^]^ Copyright 2019, American Chemical Society. (B) 1D/2D heterogeneous membrane made of PC with cylindrical nanopores and multilayered GO (left) and *I*–*V* curves of different ion transport directions (right). Reproduced with permission.^[^ ^196^ ^]^ Copyright 2019, Wiley‐VCH. (C) 1D/3D heterogeneous membrane consisting of AAO with cylindrical nanochannels and silk nanofibers (SNF) with abundant nanochannels (left) and *I*–*V* curves of the pure AAO, SNF, and heterogeneous membranes (right). Reproduced under the terms of the Creative Commons Attribution 4.0 International license.^[^ ^202^ ^]^ Copyright 2019, The Author(s). (D) 2D/2D heterogeneous membrane consisting of reconstructed negatively charged GO (n‐GO) and positively charged GO (p‐GO) (left) and *I*–*V* curves of the heterogeneous membrane (right). Reproduced with permission.^[^ ^206^ ^]^ Copyright 2019, American Chemical Society. (E) 2D/3D heterogeneous membrane composed of laminar GO and 3D porous polymer (i.e., PPSU‐Pyx) (left) and *I*–*V* curves of the heterogeneous membranes at different pH values. Reproduced with permission.^[^ ^208^ ^]^ Copyright 2017, American Chemical Society. (F) 3D/3D heterogeneous membrane consisting of nanoporous terpyridine‐modified cellulose nanofibers (TYP‐CNF) and macroporous polyvinylpyrrolidone/polyacrylonitrile (PVP/PAN) (left) and discharge capability‐cycle number curves of PP/PE/PP, TYP‐CNF on PAN, and TYP‐CNF/(PVP/PAN) (i.e., c‐mat) separators (right). Reproduced with permission.^[^ ^210^ ^]^ Copyright 2016, American Chemical Society

Owing to asymmetric factors such as asymmetric components, geometric parameters (e.g., shape and size), and interface properties (e.g., charge and wettability), heterogeneous membranes usually exhibit unique ion transport properties in contrast to intrinsic membranes. In this section, we present the typical heterogeneous membranes (i.e., 1D/1D, 1D/2D, 1D/3D, 2D/2D, 2D/3D, and 3D/3D) according to the combination forms of 1D, 2D, and 3D nanochannels (Figure 9).

#### 3.4.1 1D/1D heterogeneous membranes

Based on the material composition, 1D/1D heterogeneous membranes can be further divided into five categories: inorganic/inorganic (I/I), organic/metallic (O/M), inorganic/metallic (I/M), organic/inorganic (O/I), and organic/organic (O/O) heterogeneous membranes. I/I membranes (e.g., Al_2_O_3_/SiO_2_^[^ ^179^, ^180^, ^181^ ^]^ and CaWO_4_/MnO_2_^[^ ^68^ ^]^) are commonly composed of heterogeneous nanochannels with two types of oxide surfaces. Porous PC with Au nanoparticles (AuNPs) around pore entrances form an O/M membrane (i.e., PC/AuNPs^[^ ^182^ ^]^), whereas monolayer AuNPs assembled on AAO lead to the formation of an I/M membrane (i.e., AAO/AuNPs^[^ ^183^ ^]^). Nanochannel‐structured organic and inorganic layers are combined to construct O/I membranes (e.g., conducting polymer (CP)/AAO,^[^ ^184^, ^185^ ^]^ BCP/AAO,^[^ ^186^, ^187^ ^]^ PET/SiO_2_,^[^ ^188^ ^]^ and PI/AAO^[^ ^189^ ^]^). O/O heterogeneous membranes generally include two‐layer nanochannel‐structured polymers with different compositions (e.g., BCP/PET,^[^ ^190,191^ ^]^ PC/CP,^[^ ^99^, ^105^, ^106^ ^]^ and BCP/BCP^[^ ^192^, ^193^ ^]^), modifications (e.g., PET/PET^[^ ^194^ ^]^), and combinations (e.g., PET/PET^[^ ^195^ ^]^). Taking PET/SiO_2_ as an example, the 1D/1D heterogeneous membrane consisted of mesoporous SiO_2_ approximately 2.3 nm in diameter and 100 nm long and PET with conical nanochannels that had a 750–900 nm base diameter and a 10–15 nm tip diameter. Owing to the higher cationic selectivity of the SiO_2_ mesochannels, the membrane exhibited sensitive thermoelectric response (sensitivity of 0.71 mV K^−1^) (Figure 9A).^[^ ^188^ ^]^

#### 3.4.2 1D/2D heterogeneous membranes

Research on 1D/2D heterogeneous membranes (e.g., PC/GO,^[^ ^196^ ^]^ BCP/PET,^[^ ^197^ ^]^ and AAO/MMT^[^ ^198^ ^]^) is relatively scarce. However, an ampholytic polydopamine (PDOPA)‐modified PC with cylindrical nanochannels and negatively charged GO with 2D nanochannels were used to fabricate 1D/2D I/I heterogeneous membranes, which showed finely controlled preferential directions of ion transport under different driving forces such as electric field, concentration difference, and hydraulic pressure (Figure 9B).^[^ ^196^ ^]^ A kind of O/O heterogeneous membrane was developed by combining conical PET nanochannels with a 500 nm base diameter and a 15 nm tip diameter and BCP with an 18 nm lateral size and a thickness of 600 nm. The corresponding ion rectification and cation gating properties were able to be modulated by both pH and temperature stimuli.^[^ ^197^ ^]^

#### 3.4.3 1D/3D heterogeneous membranes

There are two main categories of 1D/3D heterogeneous membranes: I/I and O/I membranes. I/I membranes consisting of 1D nanochannel‐structured AAO and porous carbon (e.g., carbon/AAO^[^ ^199^ ^]^) or TiO_2_ (e.g., TiO_2_/AAO^[^ ^67^ ^]^) have been fabricated. O/I membranes have been constructed using 1D nanochannel‐structured AAO and interconnected 3D porous polymers such as MOFs (e.g., MOF/AAO^[^ ^153^, ^200^ ^]^), CPs (e.g., CP/AAO^[^ ^201^ ^]^), Nafion (e.g., Nafion/AAO^[^ ^112^ ^]^), and nanofibers (e.g., silk nanofibers (SNFs)/AAO^[^ ^202^ ^]^). For instance, Wen et al. designed a 1D/3D membrane consisting of amphoteric groups decorated with AAO with tunable cylindrical nanochannels and negatively charged SNFs with abundant nanochannels at approximately 20 nm (Figure 9C).^[^ ^202^ ^]^ As a result of the asymmetric geometric parameters and interface charge, the heterogeneous membrane exhibited enhanced ion transport and suppressed ion concentration polarization simultaneously.

#### 3.4.4 2D/2D heterogeneous membranes

2D/2D heterogeneous membranes are constructed by using one type of lamellar material with different modifications^[^ ^203^, ^204^, ^205^, ^206^ ^]^ or two types of lamellar materials.^[^ ^207^ ^]^ Guo et al. investigated the preferential directions of proton transport under the driving forces of electric field, concentration difference, and hydraulic pressure using a 2D/2D membrane consisting of reconstructed negatively charged GO (n‐GO) and positively charged GO (p‐GO) (Figure 9D).^[^ ^206^ ^]^ The results indicated that protons preferred to travel from the n‐GO side to the p‐GO side under the electric field while traveling in the reverse direction under other driving forces. A MoS_2_/WSe_2_ membrane showed a high ICR ratio (∼35) owing to the asymmetric characteristics (i.e., composition, interlayer spacing, and surface charge) in the separate MoS_2_ and WSe_2_ layers.^[^ ^207^ ^]^

#### 3.4.5 2D/3D heterogeneous membranes

A 2D/3D heterogeneous membrane can be developed by combining restacked nanosheets and continuous 3D porous materials. For example, Jiang et al. designed a typical 2D/3D heterogeneous membrane taking advantage of laminar GO with a negative charge and a 3D porous polymer (i.e., PPSU‐Pyx) with a tunable charge density (Figure 9E).^[^ ^208^ ^]^ The adjustable surface charge and wettability under the pH stimulus endowed the heterogeneous membrane with a reversible nano‐gating property.

#### 3.4.6 3D/3D heterogeneous membranes

It is feasible to prepare 3D/3D heterogeneous membranes on a large scale because of the simple fabrication methods.^[^ ^82^, ^209^, ^210^ ^]^ For instance, a 3D microscale porous hydrogel with a negative charge and nanoscale porous CP with a positive charge (e.g., Hydrogel/CP) were used to obtain 3D/3D heterogeneous membranes.^[^ ^82^ ^]^ The asymmetries in chemical composition, structure, and surface charge polarity in 3D/3D heterogeneous membranes can lead to ion rectification properties, which can be controlled by external stimuli (e.g., electricity and pH). An asymmetric pore‐structured separator consisting of nanoporous terpyridine‐modified cellulose nanofibers (TYP‐CNF) and macroporous polyvinylpyrrolidone/polyacrylonitrile (PVP/PAN) was designed after considering both the leakage current and ion transport rate, and it exhibited a high discharge rate capability (Figure 9F).^[^ ^210^ ^]^

As mentioned above, heterogeneous membranes with mixed‐dimensional nanochannels, that is, 1D/1D, 1D/2D 1D/3D, 2D/2D, 2D/3D, and 3D/3D membranes, generally show more controllable ion transport properties through the design of asymmetries in chemical composition, structure, and surface charge polarity and the induction of various external stimuli. It is still necessary to investigate the combination forms of different nanochannel‐structured materials for precisely tunable ion transport.

In this section, we introduced representative nanochannel‐structured membranes for ion transport based on nanochannel dimensions, which include single‐dimensional nanochannels (i.e., 1D, 2D, and 3D) and mixed‐dimensional nanochannels (i.e., 1D/1D, 1D/2D, 1D/3D, 2D/2D, 2D/3D, and 3D/3D). First, 1D nanochannel‐structured membranes were mainly classified into two categories according to the material composition: organic (e.g., PET, PI, PC, CDNTs, and COF) and inorganic (e.g., AAO, CNTs, SiO_2_, Si_3_N_4_, and C_3_N_4_) membranes. Second, representative 2D nanochannel‐structured membranes consisting of lamellar inorganic materials were discussed, including GO, C_3_N_4_, MXenes, MoS_2_, BN, kaolinite, MMT, and VOPO_4_. Then, typical membranes with continuous 3D nanochannels were reviewed, such as MOFs, COFs, CMPs, BCPs, cellulose nanofibers, ETPTA, carbon fibers, and SiO_2_ nanoparticles, as well as stacked porous nanosheets and hybrid nanosheets. Finally, we summarized the heterogeneous membranes with mixed‐dimensional nanochannels, that is, 1D/1D, 1D/2D 1D/3D, 2D/2D, 2D/3D, and 3D/3D membranes. It is believed that the abovementioned examples can guide the construction of nanochannel‐structured membranes to mediate ion transport by choosing the proper material compositions with single‐ and/or mixed‐dimensional nanochannels, different surface charge polarities, wettabilities, and external stimuli.

## 4 ULTRATHIN MEMBRANES

**FIGURE 10:**
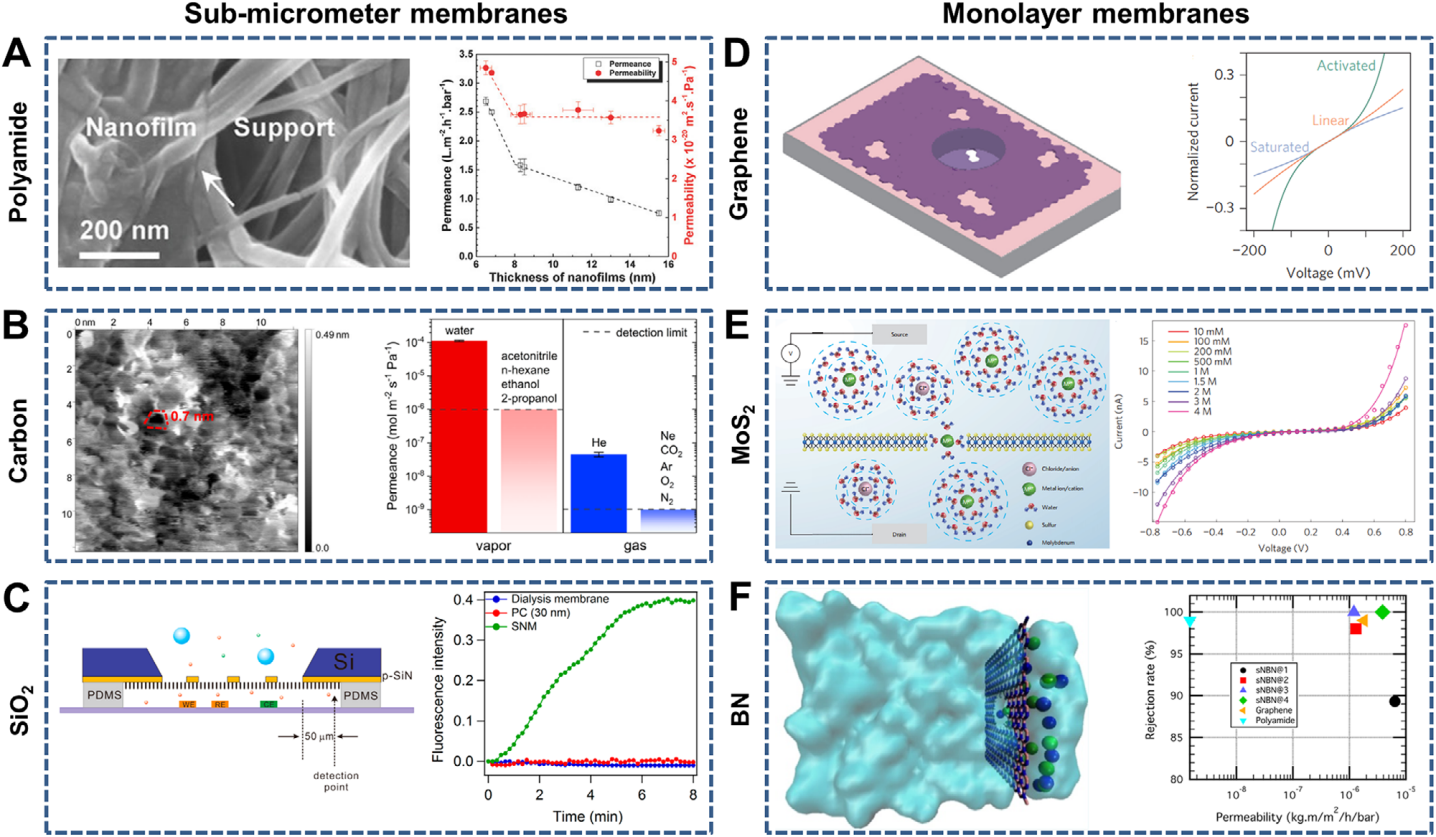
Representative ultrathin membranes (e.g., sub‐micrometer and monolayer membranes). Sub‐micrometer membranes include (A) ultrathin polyamide nanofilms (left) and water permeance and permeability versus thickness (right), (B) sub‐nanometer channel‐structured carbon membranes with thickness of ∼1.2 nm (left) and permeances of various vapors and gases (right), and (C) ultrathin SiO_2_ membranes with ordered and perpendicular nanochannels (left) and permeation flux curves of fluorescein anion across the SiO_2_ membrane, dialysis membrane and PC membrane (right). (A) Reproduced with permission.^[^ ^213^ ^]^ Copyright 2018, Wiley‐VCH. (B) Reproduced with permission.^[^ ^217^ ^]^ Copyright 2018, American Chemical Society. (C) Reproduced with permission.^[^ ^59^ ^]^ Copyright 2015, American Chemical Society. Monolayer membranes include (D) a single‐layer graphene membrane with isolated sub‐nanometer pores (left) and *I*–*V* curves with linear, activated, and saturated features (right), (E) a single‐layer MoS_2_ membrane with a sub‐nanometer pore for single‐ion transport (left) and *I*–*V* curves under varied ion concentrations (right), and (F) molecular dynamics simulations for monolayer BN membranes with sub‐nanometer pores (left) and rejection rate versus water permeability for different BN membranes (right). (D) Reproduced with permission.^[^ ^222^ ^]^ Copyright 2015, Macmillan Publishers Limited. (E) Reproduced with permission.^[^ ^224^ ^]^ Copyright 2016, Macmillan Publishers Limited. (F) Reproduced with permission.^[^ ^227^ ^]^ Copyright 2018, American Chemical Society

With decreasing membrane thickness, the mass transport resistance is significantly reduced, and the corresponding permeation flux can be enhanced.^[^ ^211^ ^]^ Thus, it is urgent to develop ultrathin membranes, especially sub‐micrometer and monolayer membranes, with superior mechanical and ion transport properties.^[^ ^35^, ^212^ ^]^ Ion transport pathways can be further decreased by introducing ordered nanopores or sub‐nanopores into the ultrathin membranes. In this section, we provide typical examples of ultrathin membranes, including sub‐micrometer (e.g., polyamide, carbon, SiO_2_) and monolayer (e.g., graphene, MoS_2_, and BN) membranes (Figure 10).

Typical examples of ultrathin membranes include sub‐micrometer (e.g., polyamide, carbon, SiO_2_) and monolayer (e.g., graphene, MoS_2_, and BN) membranes. Polyamide membranes are commonly used for desalination via reverse osmosis. To enhance membrane permeance, many studies have focused on decreasing the thickness.^[^ ^213^, ^214^, ^215^, ^216^ ^]^ For instance, transferable ultrathin polyamide nanofilms with thicknesses of less than 8 nm were prepared at the oil–water interface by interfacial polymerization.^[^ ^213^ ^]^ When the thickness was decreased to approximately 6 nm, the corresponding water permeance reached 2.7 L m^−2^ h^−1^ bar^−1^ (Figure 10A). Carbon nanomembranes with thicknesses of approximately 1.2 nm and channel diameters of approximately 0.7 nm exhibited both high water permeance (∼1.1 × 10^−4^ mol m^−2^ s^−1^ Pa^−1^) and good selectivity because the hydrogen‐bonded water inside the channel enlarged the sub‐nanopores and contributed to fast water permeation (Figure 10B).^[^ ^217^ ^]^ Ultrathin SiO_2_ membranes with ordered nanochannels are widely used for selective separation.^[^ ^59^, ^218^, ^219^ ^]^ For instance, Su et al. prepared SiO_2_ membranes (thickness of 10–120 nm) with vertically aligned nanochannels (pore size of ∼2.3 nm and porosity of 16.7%) that showed both high permeation flux and fine selectivity based on size and charge (Figure 10C).^[^ ^59^ ^]^ Monolayer membranes made of graphene, MoS_2_, and BN have also been applied for controllable ion transport. For single‐layer graphene, the 2D confined space^[^ ^220^ ^]^ between the graphene layer and the single‐crystal Pt(111) surface and the 1D single sub‐nanometer pore^[^ ^221^, ^222^, ^223^ ^]^ were both used to modulate ion transport. Karnik et al. studied the diverse ion transport behaviors through a single‐layer graphene membrane with isolated pores less than 2 nm in size, which may have been caused by ion dehydration and electrostatic interactions (Figure 10D).^[^ ^222^ ^]^ Recently, ion transport across single‐layer MoS_2_ with sub‐nanometer pores^[^ ^224^, ^225^ ^]^ and nanopores^[^ ^226^ ^]^ has also attracted the attention of researchers. For instance, a single‐layer MoS_2_ membrane (thickness of 0.65 nm) with sub‐nanometer pores (diameters of 0.6 nm) was used to study single‐ion transport behavior (Figure 10E).^[^ ^224^ ^]^ The measured *I*–*V* curves exhibited a striking nonlinear characteristic, with an apparent gap as a result of the synergistic effect of Coulomb blockade and dehydration. Ghoufi et al. reported the ultrahigh water permeability and ion rejection (∼100%) of monolayer BN membranes with sub‐nanometer pores through molecular dynamics simulations (Figure 10F).^[^ ^227^ ^]^ Most recently, Lozada‐Hidalgo et al. reported selective proton transport through monolayer BN membranes with defect‐free characteristics, showing potential applications for ion separation.^[^ ^228^ ^]^

In this section, representative ultrathin membranes with nano or sub‐nanochannels were introduced, which included sub‐micrometer (e.g., polyamide, carbon and SiO_2_,) and monolayer (e.g., graphene, MoS_2_, and BN) membranes. To achieve both high permeability and selectivity, novel materials must be screened to construct ultrathin membranes with vertically ordered channels to shorten the ion transport distance. For example, single‐layer MOFs and COFs with uniform pore sizes are ideal materials for efficient ion separation because of their short ion transport pathways.

## 5 SANDWICH‐LIKE MEMBRANES

**FIGURE 11:**
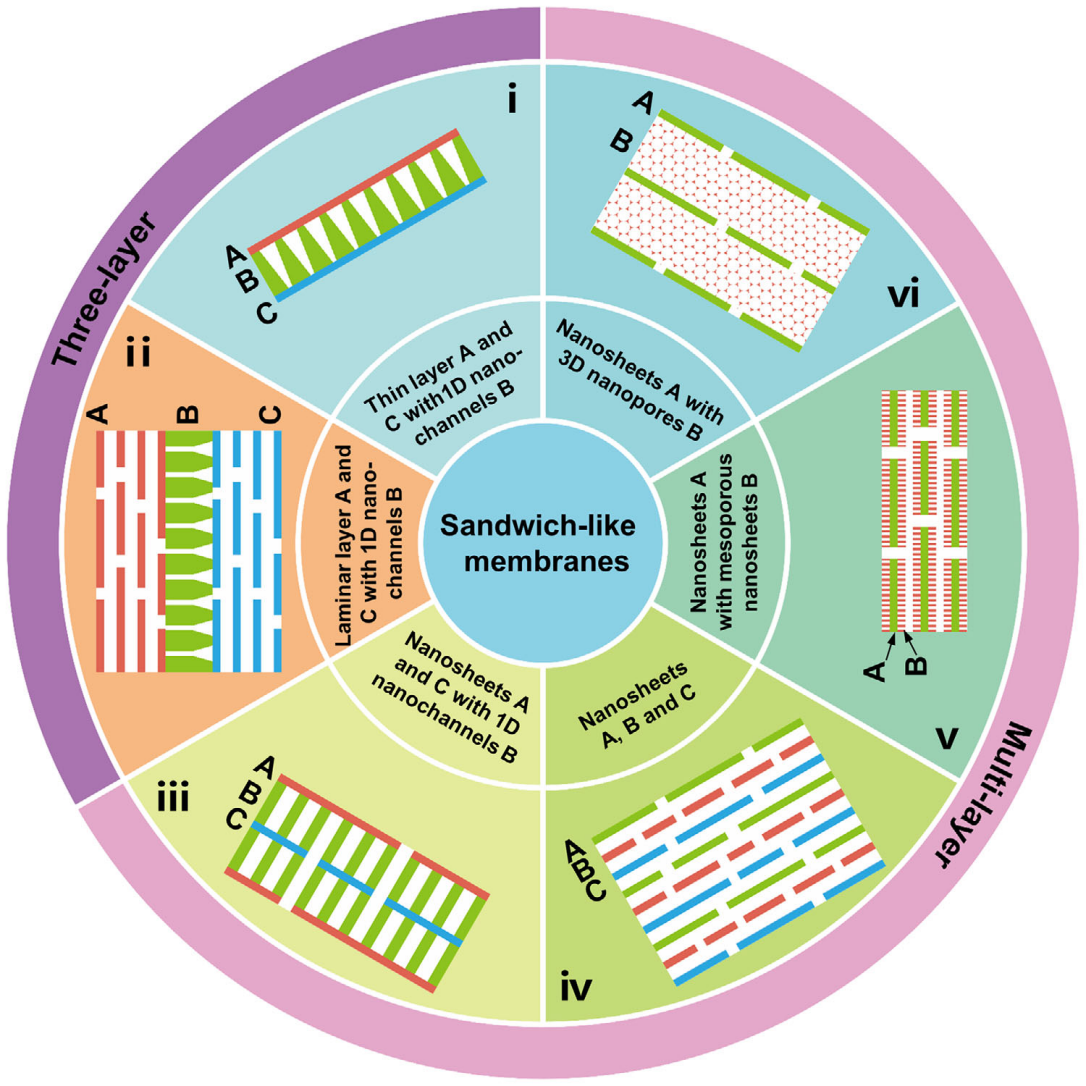
Various types of sandwich‐like membranes. Three‐layer sandwich‐like membranes, including (i) two thin layers A and C combined with 1D nanochannels B and (ii) two laminar layers A and C combined with 1D nanochannels B. Multilayer sandwich‐like membranes, including (iii) two kinds of nanosheets A and C combined with 1D nanochannels B, (iv) restacked by three kinds of nanosheets A, B, and C, (v) sandwich‐like nanosheets A decorated with mesoporous nanosheets B, and (vi) nanosheets A combined with 3D nanopores B

**FIGURE 12:**
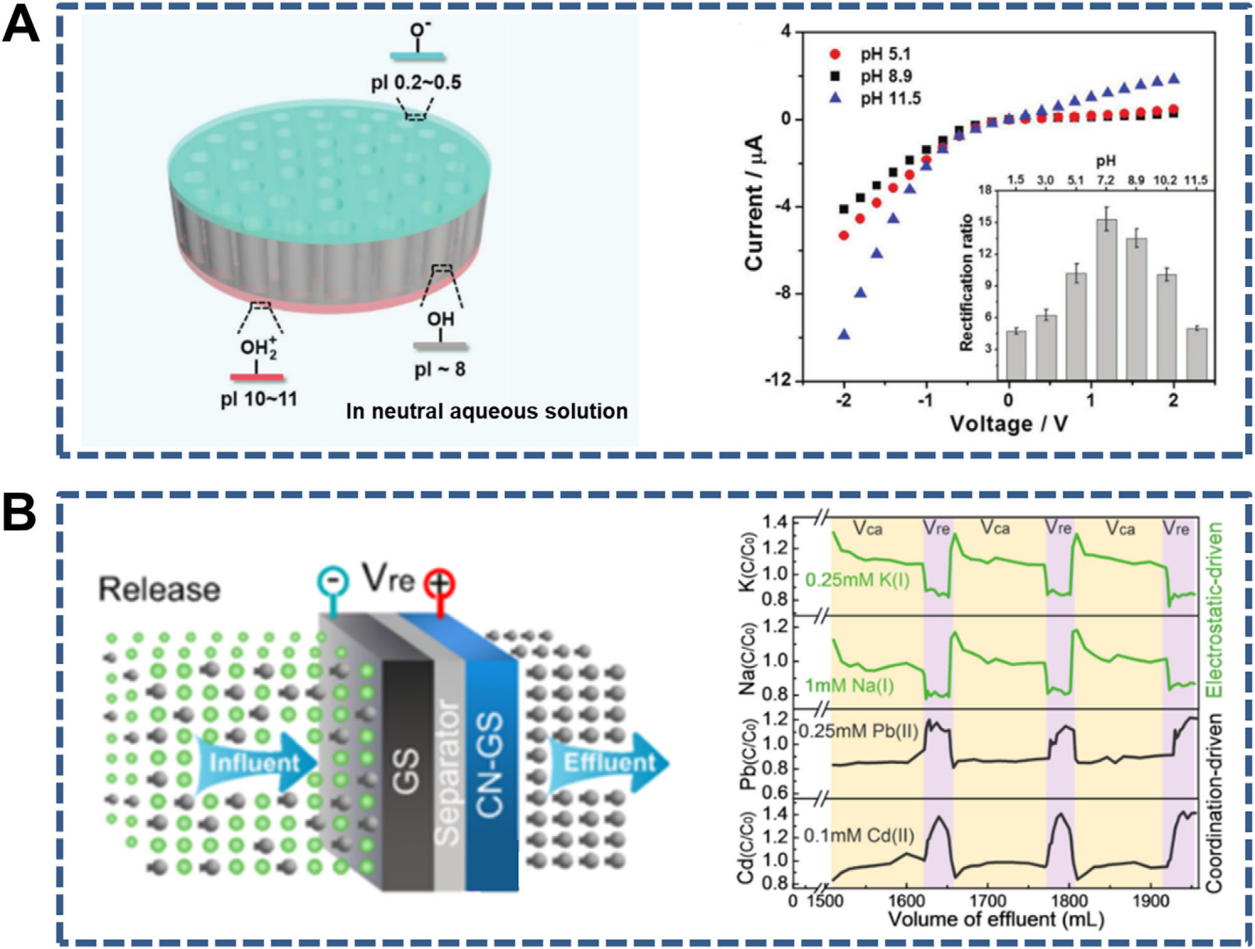
Three‐layer sandwich‐like membranes for ion transport. (A) WO_3_/AAO/NiO membranes (left) and *I*–*V* curves at varied pH values (right). Reproduced under the terms of the Creative Commons CC BY license.^[^ ^37^ ^]^ Copyright 2018, The Author(s). (B) Sandwich‐like membranes consisting of *N*‐functionalized graphene sheet hydrogel (GS) layer, 1D porous separator, and *N*‐functionalized GS (CN‐GS) layer (left) and variation of ion concentrations during the separation process (right). Reproduced with permission.^[^ ^38^ ^]^ Copyright 2015, American Chemical Society

**FIGURE 13:**
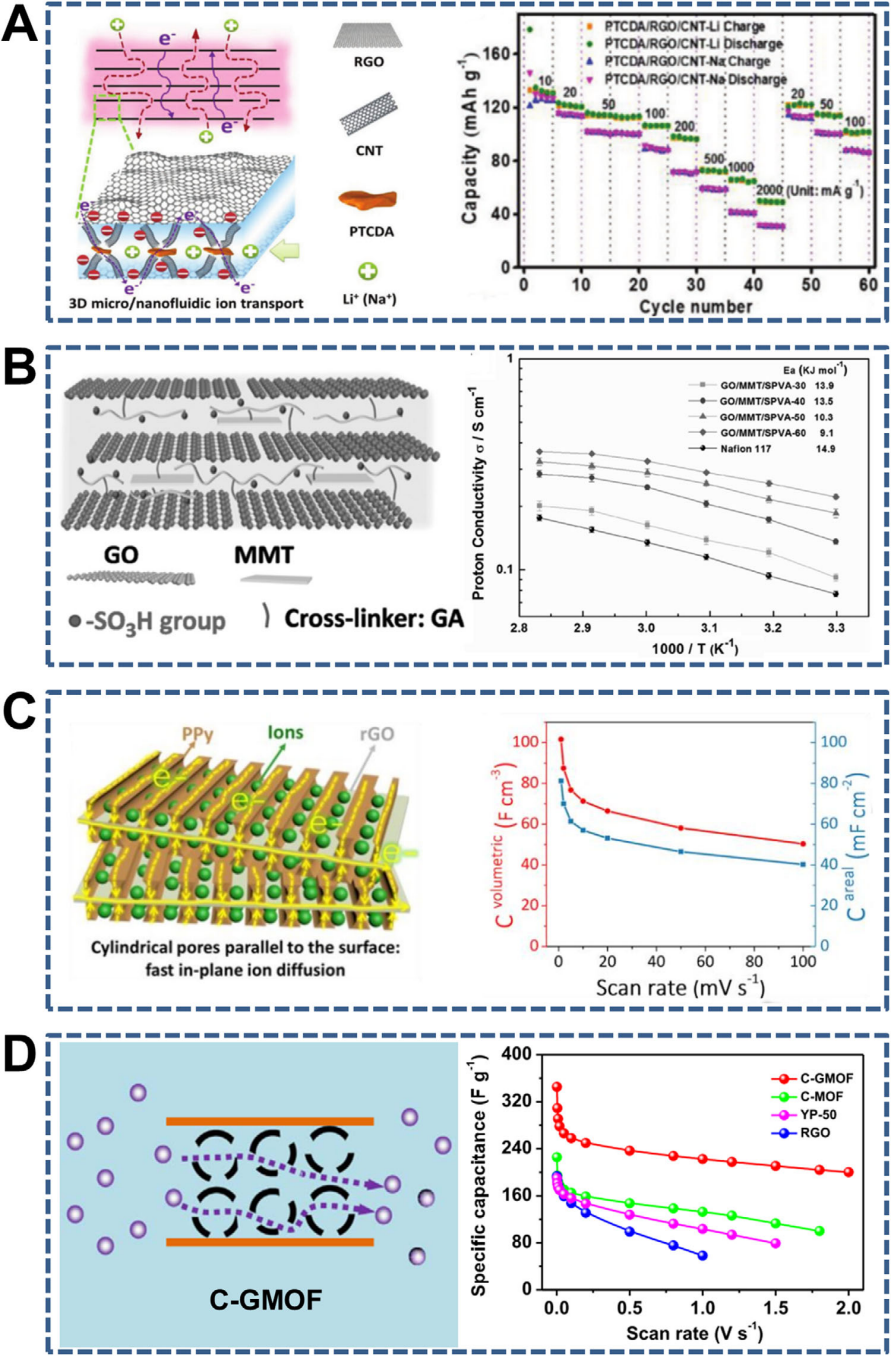
Multilayer sandwich‐like membranes for ion transport. (A) Sandwich‐like electrodes (PTCDA/rGO/CNT) composed of 3,4,9,10‐perylenetetracarboxylic dianhydride (PTCDA), rGO, and CNTs (left) and rate performance at varied current densities (right). Reproduced with permission.^[^ ^229^ ^]^ Copyright 2018, Wiley‐VCH. (B) Sandwich‐like composite membranes (GO/MMT/SPVA) consisting of GO, MMT, and sulfonated polyvinyl alcohol (SPVA) (left) and proton conductivity of the composite membranes (right). Reproduced with permission.^[^ ^230^ ^]^ Copyright 2017, Wiley‐VCH. (C) Sandwich‐like hybrid nanomembranes (PPy/rGO/PPy) composed of rGO decorated with polypyrrole (PPy) on both sides (left) and areal (blue) and volumetric (red) capacitance versus scan rate (right). Reproduced with permission.^[^ ^231^ ^]^ Copyright 2019, Wiley‐VCH. (D) Sandwich‐like membranes (i.e., C‐GMOF) made of graphene and mesoporous carbon (left) and specific capacitance versus scan rate (right). Reproduced with permission.^[^ ^232^ ^]^ Copyright 2016, Elsevier Ltd

To precisely tune ion transport behaviors, sandwich‐like membranes with more delicate designing of nanochannels and combination structures have been prepared. In this section, we introduce sandwich‐like membranes with different combination forms (Figure 11) gathered from recent research (Figures 12 and  13). Sandwich‐like membranes can be classified into two types: three‐layer sandwich‐like membranes comprising (i) two thin layers A and C combined with 1D nanochannels B and (ii) two laminar layers A and C combined with 1D nanochannels B, and multilayer sandwich‐like membranes comprising (iii) two kinds of nanosheets A and C combined with 1D nanochannels B, (iv) restacked by three kinds of nanosheets A, B and C, (v) sandwich‐like nanosheets A decorated with mesoporous nanosheets B, and (vi) nanosheets A combined with 3D nanopores B (Figure 11).

Recent studies on sandwich‐like membranes for ion transport are discussed from the perspective of three‐layer and multilayer sandwich‐like membranes. Through the combination of 1D nanochannel‐structured AAO and two layers of different inorganic oxides with thicknesses of several hundred nanometers and different isoelectric points, a typical three‐layer sandwich‐like WO_3_/AAO/NiO membrane was constructed that exhibited efficient modulation of the ionic transport owing to the opposite charges on the exterior surfaces (Figure 12A).^[^ ^37^ ^]^ Another example is a sandwich‐like electric double‐layer separation (EDLS) membrane consisting of an *N*‐functionalized graphene sheet hydrogel (GS) layer, a 1D porous separator, and an *N*‐functionalized GS (CN‐GS) layer (Figure 12B).^[^ ^38^ ^]^ The lamellar structure of the CN‐GS layer selectively allowed ions to be transported along the 2D nanochannels under the control of the surface potential. Compared with three‐layer sandwich‐like membranes, multilayer sandwich‐like membranes are more common in mediating ion transport. For instance, 3,4,9,10‐perylenetetracarboxylic dianhydride (PTCDA), rGO, and CNTs were used to construct sandwich‐like electrodes (PTCDA/rGO/CNTs) with interconnected micro/nano channels, which showed high ion diffusion coefficients and superior rate performances in lithium/sodium‐ion batteries (Figure 13A).^[^ ^229^ ^]^ Taking advantage of GO, MMT, and sulfonated polyvinyl alcohol (SPVA), Jiang et al. designed sandwich‐like composite membranes (GO/MMT/SPVA) with both high ionic conductivity (326 mS cm^−1^) and high mechanical properties (tensile strength of 250 MPa), which were owing to the continuous 2D nanochannels and special brick‐and‐mortar structure, respectively (Figure 13B).^[^ ^230^ ^]^ Very recently, sandwich‐like hybrid nanomembranes (PPy/rGO/PPy) were fabricated as supercapacitors with superior volumetric capacitance (102 F cm^−3^) using rGO decorated with polypyrrole (PPy) on both sides (Figure 13C).^[^ ^231^ ^]^ The cylindrical mesopores of PPy can facilitate rapid ionic diffusion in the plane. Fan et al. also constructed supercapacitors with superior specific capacitance of 345 F g^−1^ at 2 mV s^−1^ using sandwich‐like carbon with confined nanochannels between GO nanosheets (Figure 13D).^[^ ^232^ ^]^ In this system, the nanochannels enabled fast electrolyte ion transport while the GO nanosheets functioned as conductive and structural supports.

In this section, the reported sandwich‐like membranes were classified into two types (i.e., three‐layer and multilayer sandwich‐like membranes) based on their combination forms, and simplified models were also presented (Figure 11). More specifically, three‐layer sandwich‐like membranes include: (i) two thin layers A and C combined with 1D nanochannels B and (ii) two laminar layers A and C combined with 1D nanochannels B, whereas the multilayer ones include (iii) two kinds of nanosheets A and C combined with 1D nanochannels B, (iv) restacked by three kinds of nanosheets A, B and C, (v) sandwich‐like nanosheets A decorated with mesoporous nanosheets B, and (vi) nanosheets A combined with 3D nanopores B. Owing to the fine structural adjustment, these membranes can mediate ion transport with specific requirements. To date, there have been few studies on sandwich‐like membranes, in contrast to membranes with single‐ and mixed‐dimensional nanochannels. Therefore, it is meaningful to construct sandwich‐like membranes with more delicate structures and further investigate the corresponding ion transport properties.

## 6 STIMULUS‐RESPONSIVE NANOCHANNELS

**FIGURE 14:**
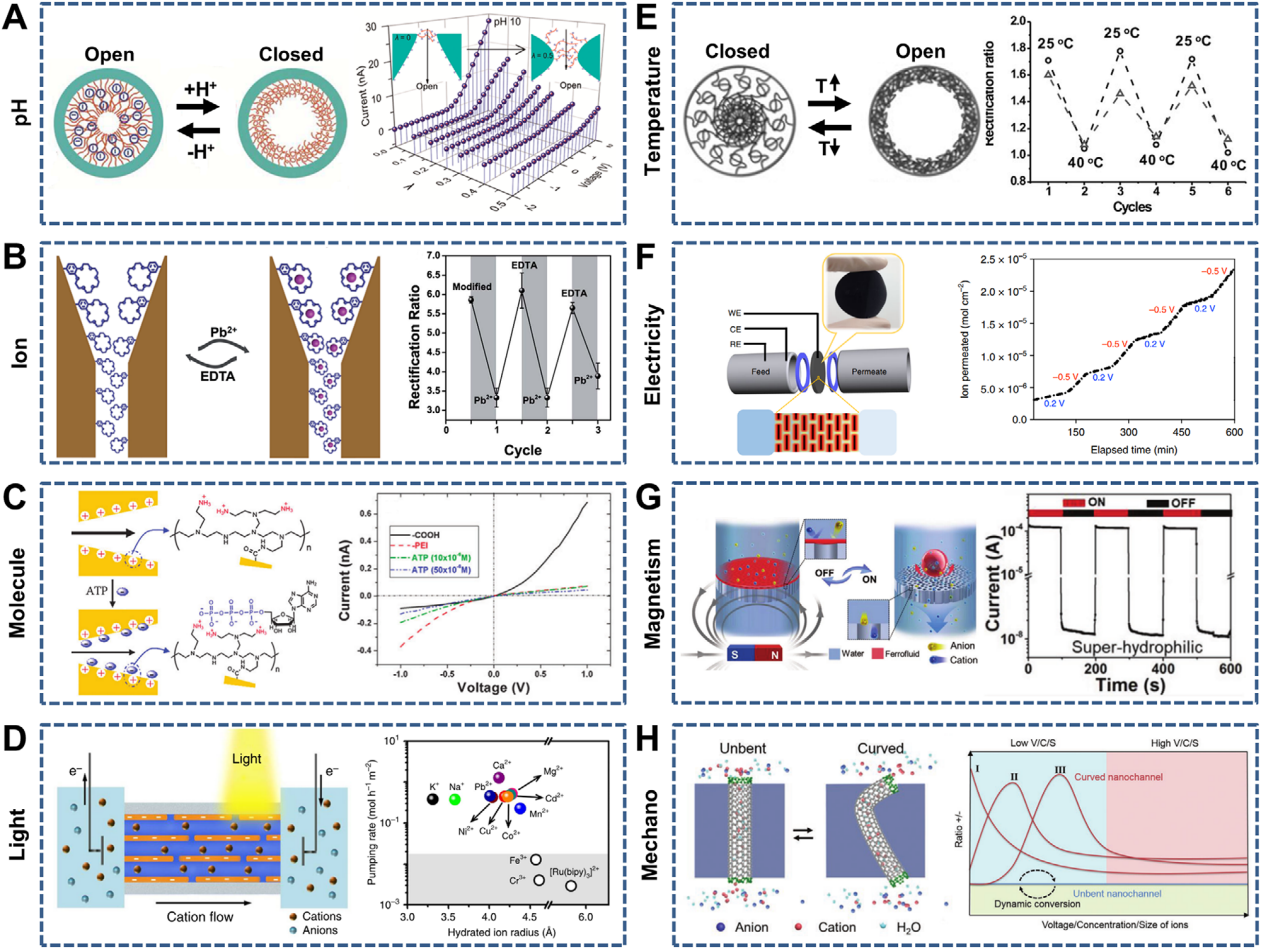
**S**ingle‐responsive nanochannels. (A) pH‐Responsive hourglass‐shaped PET nanochannels through adjusting the shape asymmetries and poly(acrylic acid) (PAA) locations (left) and *I*–*V* curves of the nanochannels modified with PAA on the small base sides at pH 10 (right). Reproduced with permission.^[^ ^75^ ^]^ Copyright 2015, American Chemical Society. (B) Ion‐responsive funnel‐shaped nanochannels modified with 18‐crown‐6 units (left) and reversibly controlled ion rectification after alternately adding Pb^2+^ and ethylenediamine tetraacetic acid (EDTA) (right). Reproduced with permission.^[^ ^235^ ^]^ Copyright 2018, The Royal Society of Chemistry. (C) Inversion of surface charge in single conical PET nanochannel modified with polyethylenimine (left) and *I*–*V* curves of the nanochannels before and after adding ATP (right). Reproduced with permission.^[^ ^238^ ^]^ Copyright 2010, The Royal Society of Chemistry. (D) Lamellar GO membranes with 2D nanochannels (left) and pumping rates of varied ions against the concentration gradient (right). Reproduced under the terms of the Creative Commons CC BY license.^[^ ^239^ ^]^ Copyright 2019, The Author(s). (E) Temperature‐responsive single conical PET nanochannel (left) and reversible switching of the rectifying state and the non‐rectifying state (right). Reproduced with permission.^[^ ^242^ ^]^ Copyright 2010, Wiley‐VCH. (F) Lamellar GO (left) and reversibly modulating ion transport with voltages of 0.2 and −0.5 V (right). Reproduced with permission.^[^ ^78^ ^]^ Copyright 2018, Macmillan Publishers Limited. (G) Magnetism‐responsive nanochannels consisting of the superhydrophilic AAO and magnetically controlled ferrofluid‐ (left) and magnetic‐modulated gating (right). Reproduced with permission.^[^ ^247^ ^]^ Copyright 2018, Wiley‐VCH. (H) Curved CNTs (left) and various ion transport behaviors (right). Reproduced with permission.^[^ ^249^ ^]^ Copyright 2019, Wiley‐VCH

**FIGURE 15:**
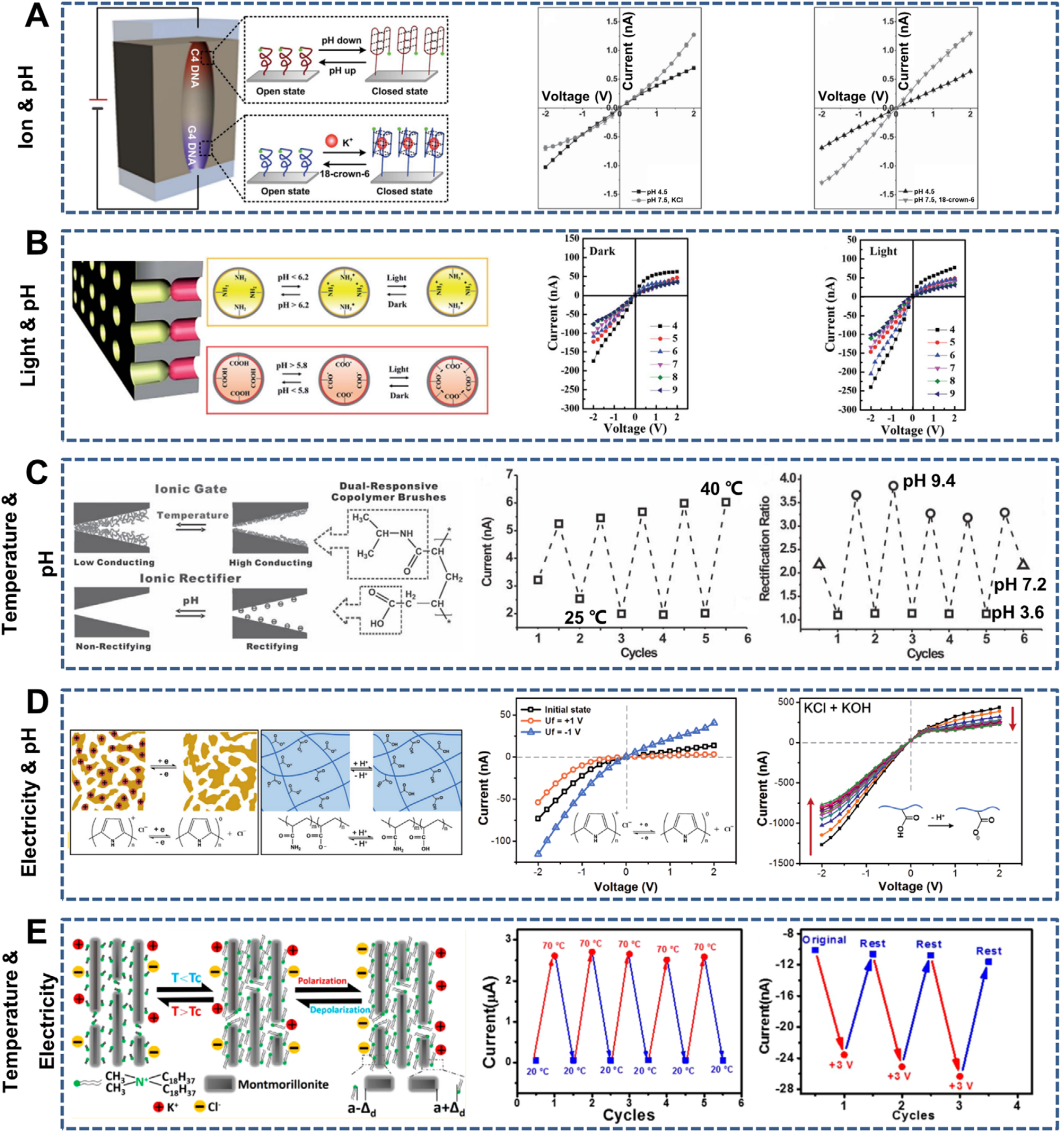
Dual‐responsive nanochannels. (A) K^+^ and pH‐responsive cigar‐shaped PET nanochannels (left). *I*–*V* curves of the nanochannels with pH 4.5 and pH 7.5, KCl (middle) and with pH 4.5, KCl and pH 7.5, 18‐crown‐6 (right). Reproduced with permission.^[^ ^80^ ^]^ Copyright 2014, Wiley‐VCH. (B) pH and light‐responsive hourglass‐shaped AAO nanochannels (left). *I*–*V* curves of the nanochannels at varied pH values in dark (middle) and light (right) states. Reproduced with permission.^[^ ^81^ ^]^ Copyright 2016, The Royal Society of Chemistry. (C) Temperature and pH‐responsive conical PI nanochannel (left). Temperature‐dependent ion conducting (middle) and pH‐dependent ion rectification (right). Reproduced with permission.^[^ ^253^ ^]^ Copyright 2010, Wiley‐VCH. (D) Electricity and pH‐responsive 3D/3D heterogeneous nanochannels (left). *I*–*V* curves at initial, oxidation and reduction states (middle) and *I*–*V* curves with adding 0.1 M KOH (right). Reproduced with permission.^[^ ^82^ ^]^ Copyright 2017, Wiley‐VCH. (E) Temperature and electricity‐responsive 2D MMT nanochannels (left). Ion current at 20 and 70 °C (middle) and at polarized and depolarized states (right). Reproduced with permission.^[^ ^145^ ^]^ Copyright 2017, American Chemical Society

Applying external stimuli to target nanochannels is a major strategy for achieving dynamically controllable ion transport. Therefore, it is necessary to investigate the corresponding responsiveness to various stimuli (e.g., pH, ions, molecules, light, temperature, electricity, magnetism, and mechano). Here, we introduce typical stimulus‐responsive nanochannels from two aspects, namely, single‐responsive and dual‐responsive nanochannels (Figures 14 and  15, respectively).

### 6.1 Single‐responsive nanochannels

Many studies have been carried out to study the ion transport properties in single‐responsive nanochannels under stimuli such as pH, ions, molecules, light, temperature, electricity, magnetism, and mechano. pH‐responsive nanochannels can be obtained by modifying pH‐responsive molecules, including both homogeneous modifications (e.g., lysine and histidine,^[^ ^233^ ^]^ poly 2‐(methacryloyloxy)ethyl phosphate (PMEP),^[^ ^234^ ^]^ histidine (His), and PDOPA^[^ ^83^ ^]^) and heterogeneous modifications (e.g., ethanediamine [EN],^[^ ^85^ ^]^ poly(acrylic acid) [PAA],^[^ ^75^, ^98^ ^]^ APTMS,^[^ ^110^ ^]^ and polyvinylpyridine [PVP]/PAA^[^ ^95^ ^]^]. For instance, Jiang et al. constructed pH‐responsive hourglass‐shaped PET nanochannels with adjustable shape asymmetries and pH‐responsive polyelectrolyte (PAA) locations (Figure 14A).^[^ ^75^ ^]^ The results indicated that the nanochannels modified with PAA on the small base sides at pH 10 showed improved asymmetric ion transport characteristics owing to the larger charge gradients. Ion‐responsive nanochannels are usually functionalized with molecules (e.g., 4′‐aminobenzo‐18‐crown‐6 (18‐crown‐6 units)^[^ ^70^, ^235^ ^]^ and oligo(aryl‐triazole)s^[^ ^236^ ^]^) and ions (e.g., Fe^3+[^ ^64^ ^]^ and Ce^3+[^ ^65^ ^]^], which can be responsive to cations (e.g., K^+^, Pb^2+^, Fe^3+^, and Ce^3+^) and anions (e.g., Cl^−^). Because of the recognition interaction between Pb^2+^ and 18‐crown‐6 units, the funnel‐shaped nanochannels modified with 18‐crown‐6 units exhibited reversibly controlled ion rectification after alternately adding Pb^2+^ and ethylenediamine tetraacetic acid (EDTA) (Figure 14B).^[^ ^235^ ^]^ With the introduction of external molecules, the microenvironment around the nanochannels began to change, showing the switching of surface charge through H_2_O_2_^[^ ^237^ ^]^ and ATP,^[^ ^238^ ^]^ the conformational transition of modified polymers caused by inositol phosphate (InsP),^[^ ^111^ ^]^ and the breakdown of the acid–base equilibrium led by fructose.^[^ ^106^ ^]^ For example, Ensinger et al. reported that a molecule‐responsive single conical PET nanochannel showed inverse rectification after adding ATP as a result of the switching of the surface charge from positive to negative (Figure 14C).^[^ ^238^ ^]^ Light‐responsive nanochannels can be achieved using materials with intrinsic properties (e.g., GO,^[^ ^239^ ^]^ C_3_N_4_,^[^ ^121^, ^122^, ^124^ ^]^ and MXene^[^ ^139^ ^]^) or modified photo‐responsive molecules (e.g., spiropyran,^[^ ^203^, ^240^ ^]^ *cis*‐bis‐(4,4′‐dicarboxy‐2,2′‐bipyridine) dithiocyanato ruthenium(II) (N3)/spiropyran,^[^ ^113^ ^]^ photoacid (HA)/photobase (BOH),^[^ ^204^ ^]^ pillararene (P6A)/azobenzene (AZO),^[^ ^24^ ^]^ and GO/AZO‐DNA^[^ ^76^ ^]^). Very recently, Guo et al. discovered ion transport against the concentration gradient in 2D nanochannels of restacked lamellar GO membranes owing to the redistribution of the interface charge caused by asymmetric light illumination (Figure 14D).^[^ ^239^ ^]^ Because of the trans‐nanochannel diffusion potential generated by temperature change^[^ ^188^ ^]^ or the phase transition of the modified temperature‐responsive polymers, such as polystyrene‐(ethylene‐butylene)‐polystyrene (SEBS)^[^ ^241^ ^]^ and poly(*N*‐isopropylacrylamide) (PNIPAM)^[^ ^242^ ^]^), the prepared nanochannels showed temperature‐responsive ion transport. For example, a single conical PET nanochannel decorated with PNIPAM brushes exhibited temperature‐responsive switching behavior between the rectifying state and the non‐rectifying state owing to the phase transition of PNIPAM (Figure 14E).^[^ ^242^ ^]^ Electrically responsive nanochannels can be fabricated by using conducting materials with controllable surface potentials (e.g., layered graphene,^[^ ^78^, ^205^, ^243^, ^244^ ^]^ MXenes,^[^ ^245^ ^]^ mesoporous carbon,^[^ ^164^ ^]^ and nanoporous silicon^[^ ^246^ ^]^) or by modifying CPs with redox properties.^[^ ^185^, ^201^ ^]^ Li et al. reported that low voltage (under 0.5 V) improved the ion diffusion rates up to 4–7 times under nanoconfinement (below 2 nm) in lamellar GO (Figure 14F).^[^ ^78^ ^]^ This kind of rapid ion transport induced by a low surface potential can provide a basis for investigating ion transport in biological systems. In addition, magnetic‐responsive nanochannels were obtained through the combination of superhydrophilic AAO and a magnetically controlled ferrofluid, which showed a high gating ratio of 10,000 owing to the synergistic effect of both the magnetic force and the interfacial tension (Figure 14G).^[^ ^247^ ^]^ Mechanosensitive nanochannels have also attracted the attention of researchers, including CNTs,^[^ ^248^, ^249^ ^]^ graphene crown ethers,^[^ ^250^, ^251^ ^]^ and single‐layer MoS_2_ with sub‐nanometer pores.^[^ ^252^ ^]^ Most recently, Hou et al. reported a dynamic nanochannel system composed of CNTs embedded in PDMS (Figure 14H).^[^ ^249^ ^]^ Under external forces, the curvature of the CNTs could be dynamically changed, showing adjustable ion rectification in real time.

### 6.2 Dual‐responsive nanochannels

Ion transport across the nanochannels is usually simultaneously affected by different factors, and it is necessary to investigate the ion transport properties under dual and multiple stimuli. Here, we present the research on dual‐responsive nanochannels, including ions and pH, light and pH, temperature and pH, electricity and pH, and temperature‐ and electricity‐responsive nanochannels (Figure 15). In 2014, Jiang et al. designed K^+^‐ and pH‐responsive cigar‐shaped PET nanochannels through the heterogeneous modification of C‐quadruplex (C4) and G‐quadruplex (G4) DNA molecules on the top and bottom tips, respectively (Figure 15A).^[^ ^80^ ^]^ Under the influence of K^+^ and H^+^ concentrations, the nanochannels opened and closed alternately or simultaneously through the conformational transition of the C4 and G4 DNA molecules. Hourglass‐shaped AAO nanochannels decorated with both di‐tetrabutylammonium *cis*‐bis(isothiocyanato)bis(2,2′‐bipyr‐idyl‐4,4′‐dicarboxylato) ruthenium(II) (N719) and (3‐aminopropyl)triethoxysilane (APTES) also exhibited dual‐responsive ion transport owing to the redistribution of surface charge caused by pH and light (Figure 15B).^[^ ^81^ ^]^ The conical PI nanochannels showed dual‐responsive properties after decorating with temperature‐ and pH‐responsive poly(*N*‐isopropyl acrylamide‐*co*‐acrylic acid) [P(NIPAAm‐*co*‐AAc)] (Figure 15C).^[^ ^253^ ^]^ More specifically, the temperature‐induced conformational transition of P(NIPAAm‐*co*‐AAc) led to the opening and closing of the nanochannels, showing reversible switching between high and low ion conduction. The surface charge on the asymmetric nanochannel walls, adjusted by the pH, resulted in pH‐dependent ion rectification. In addition, electricity and pH‐responsive 3D/3D heterogeneous membranes consisting of an electricity‐responsive PPy layer with nanopores and a pH‐responsive poly(acrylamide‐*co*‐acrylic acid) (P(AAm‐*co*‐AA)) hydrogel layer with micropores (Figure 15D) were fabricated.^[^ ^82^ ^]^ These membranes showed dual‐responsive ion rectification because of the asymmetric chemical composition, structure, and surface charge induced by electrical and pH stimuli. Furthermore, 2D MMT intercalated with a quaternary ammonium bilayer showed temperature‐ and electricity‐responsive ion transport owing to the adjustments of the temperature‐dependent phase state of the bilayers and the electrically dependent surface polarity (Figure 15E).^[^ ^145^ ^]^

Common stimulus‐responsive nanochannels, such as single‐responsive (e.g., pH, ions, molecules, light, temperature, electricity, magnetism, and mechano) and dual‐responsive (e.g., ions and pH, light and pH, temperature and pH, electricity and pH, and temperature and electricity) nanochannels have been summarized in this section. Through precisely adjustable stimuli, the ion transport characteristics can be tuned dynamically and reversibly, which can establish the basis for further investigation of the sophisticated ion transport behaviors under multiple stimuli in both artificial and biological systems.

## 7 CONSTRUCTION METHODS OF NANOCHANNEL‐STRUCTURED MEMBRANES

The common methods for constructing nanochannel‐structured membranes with specific structures (e.g., nanochannel, ultrathin, and sandwich‐like structures) have been introduced in previous reviews^[^ ^27^, ^28^, ^29^, ^33^, ^36^, ^74^, ^156^, ^254^, ^255^ ^]^. Therefore, we will briefly list the typical fabrication approaches for designing nanochannel‐structured membranes using top‐down and bottom‐up approaches in this section (Table 1).

**TABLE 1:** Construction methods of nanochannel‐structured membranes with specific structures for controlling ion transport

Methods		Feature	Ref.
Top‐down approach	Track etching	Symmetric (e.g., cylindrical^[^ ^93^, ^256^ ^]^ and cigar‐shaped^[^ ^94^, ^95^ ^]^) and asymmetric (e.g., cone‐,^[^ ^24^, ^99^, ^104^, ^257^ ^]^ funnel‐,^[^ ^97^ ^]^ hourglass‐,^[^ ^75^, ^98^ ^]^ and bullet‐shaped^[^ ^83^, ^84^ ^]^) nanochannels	^[^ ^24^, ^75^, ^83^, ^84^, ^93^, ^94^, ^95^, ^97^, ^98^, ^99^, ^104^, ^256^, ^257^ ^]^
	Anodization	AAO with cone^[^ ^112^ ^]^ and hourglass‐shaped nanochannels,^[^ ^113^ ^]^ TiO_2_ nanotubes^[^ ^258^ ^]^	^[^ ^112^, ^113^, ^258^ ^]^
	Metal nanoparticle‐assisted etching	SiO_2_ with conical nanopores^[^ ^115^ ^]^	^[^ ^115^ ^]^
	Reactive ion etching	Si_3_N_4_ with low‐aspect‐ratio nanopores^[^ ^259^, ^260^ ^]^	^[^ ^259^, ^260^ ^]^
	Electron irradiation	Single‐layer MoS_2_ with a single sub‐nanometer pore^[^ ^224^ ^]^	^[^ ^224^ ^]^
	Chemical delignification	Aligned nanochannels consisting of cellulose nanofibers^[^ ^43^, ^44^, ^46^, ^307^ ^]^	^[^ ^43^, ^44^, ^46^, ^307^ ^]^
	Pyrolysis	Porous carbon^[^ ^172^, ^173^, ^174^, ^288^ ^]^	^[^ ^172^, ^173^, ^174^, ^288^ ^]^
Bottom‐up approach	Chemical vapor deposition (CVD)	Single‐layer graphene with nanopores^[^ ^261^ ^]^ or a single sub‐nanometer pore^[^ ^221^, ^222^, ^223^ ^]^	^[^ ^221^, ^222^, ^223^, ^261^ ^]^
	Atomic layer deposition (ALD)	Heterogeneous Al_2_O_3_/SiO_2_ nanochannels^[^ ^181^ ^]^	^[^ ^181^ ^]^
	Magnetron sputtering	Sandwich‐like WO_3_/AAO/NiO^[^ ^37^ ^]^	^[^ ^37^ ^]^
	Vacuum filtration	Layered membranes (e.g., GO,^[^ ^61^, ^262^ ^]^ C_3_N_4_,^[^ ^135^ ^]^ MXene,^[^ ^136^, ^139^ ^]^ BN,^[^ ^263^ ^]^ and kaolinite^[^ ^144^ ^]^), I/I (e.g., CaWO_4_/MnO_2_,^[^ ^68^ ^]^ GO/AAO,^[^ ^196^ ^]^ and GO/GO^[^ ^206^, ^264^, ^265^, ^266^ ^]^), O/I (e.g., SNF/AAO^[^ ^202^ ^]^ and PPSU‐Pyx/GO^[^ ^208^ ^]^), O/O heterogeneous membranes (e.g., TYP‐CNF/(PVP/PAN)^[^ ^210^ ^]^)	^[^ ^61^, ^68^, ^135^, ^136^, ^139^, ^144^, ^196^, ^202^, ^206^, ^208^, ^210^, ^262^, ^263^, ^264^, ^265^, ^266^ ^]^
	Coating	3D mesoporous carbon^[^ ^165^ ^]^, I/I (e.g., TiO_2_/AAO^[^ ^67^ ^]^), O/I (e.g., BCP/AAO,^[^ ^186^ ^]^ PI/AAO,^[^ ^189^ ^]^ and MOF/AAO^[^ ^153^ ^]^), O/O (e.g., BCP/BCP,^[^ ^192^, ^193^ ^]^ BCP/PET,^[^ ^190^, ^191^, ^197^ ^]^ and PET/CP^[^ ^99^ ^]^) and O/M heterogeneous membranes (e.g., PC/AuNPs^[^ ^267^ ^]^)	^[^ ^67^, ^99^, ^153^, ^165^, ^186^, ^189^, ^190^, ^191^, ^192^, ^193^, ^197^, ^267^ ^]^
	Spinning	Highly aligned GO gel fibers^[^ ^131^ ^]^ and Ti_3_C_2_T*_x_* fibers^[^ ^340^ ^]^ through wet spinning, porous carbon^[^ ^166^, ^167^ ^]^ and TiO_2_ fibers^[^ ^268^ ^]^ through electrospinning	^[^ ^131^, ^166^, ^167^, ^268^, ^340^ ^]^
	Assembly	AuNPs with cone‐like nanochannels,^[^ ^269^ ^]^ HGF,^[^ ^171^ ^]^ SiO_2_ nanoparticles with 3D nanochannels,^[^ ^169^ ^]^ and 2D angstrom‐scale channels composed of graphite and BN^[^ ^143^ ^]^	^[^ ^143^, ^169^, ^171^, ^269^ ^]^
	Vapor deposition polymerization (VDP)	C_3_N_4_ with 1D (e.g., conical^[^ ^124^ ^]^ and cylindrical^[^ ^121^, ^122^ ^]^ nanotubes) and 2D (e.g., layered C_3_N_4_^[^ ^134^ ^]^) nanochannels	^[^ ^121^, ^122^, ^124^, ^134^ ^]^
	Interfacial polymerization	Polyamide^[^ ^213^, ^214^, ^270^ ^]^ through chemical‐polymerization, CMF,^[^ ^158^, ^159^ ^]^ O/O (e.g., PC/CP^[^ ^105^, ^106^ ^]^ and hydrogel/CP^[^ ^82^ ^]^) and O/I heterogeneous membranes (e.g., CP/AAO^[^ ^184^, ^185^, ^201^ ^]^) through electro‐polymerization	^[^ ^82^, ^105^, ^106^, ^158^, ^159^, ^184^, ^185^, ^201^, ^213^, ^214^, ^270^ ^]^
	Mechanical shearing	Vertically aligned MXenes^[^ ^40^ ^]^	^[^ ^40^ ^]^
	Stöber solution growth	SiO_2_ with perpendicular nanochannels^[^ ^59^, ^188^, ^218^, ^219^, ^271^ ^]^	^[^ ^59^, ^188^, ^218^, ^219^, ^271^ ^]^
	Micelle template	CPs with ordered mesopores^[^ ^231^, ^272^, ^273^ ^]^	^[^ ^231^, ^272^, ^273^ ^]^

Top‐down approaches are mainly used to induce nanochannels in the membranes, taking advantage of various etching methods such as track etching, anodization, metal nanoparticle‐assisted etching, and reactive ion etching. The track‐etching method is usually used to fabricate symmetric (e.g., cylindrical‐^[^ ^93^, ^256^ ^]^ and cigar‐shaped nanochannels^[^ ^94^, ^95^ ^]^) and asymmetric 1D nanochannels (e.g., cone‐,^[^ ^24^, ^99^, ^104^, ^257^ ^]^ funnel‐,^[^ ^97^ ^]^ hourglass‐,^[^ ^75^, ^98^ ^]^ and bullet‐shaped nanochannels^[^ ^83^, ^84^ ^]^) in polymer membranes (e.g., PET, PC, and PI). The anodization method was used to develop AAO with cone‐^[^ ^112^ ^]^ and hourglass‐shaped nanochannels^[^ ^113^ ^]^ and TiO_2_ nanotubes.^[^ ^258^ ^]^ SiO_2_ with conical nanopores,^[^ ^115^ ^]^ Si_3_N_4_ with low‐aspect‐ratio nanopores,^[^ ^259^, ^260^ ^]^ and single‐layer MoS_2_ with a single sub‐nanoscale pore^[^ ^224^ ^]^ can be obtained through metal nanoparticle‐assisted etching, reactive ion etching, and electron irradiation methods, respectively. Chemical delignification and pyrolysis treatments can be used to construct 3D nanochannels in cellulose nanofibers and porous carbon, respectively.

Bottom‐up approaches include different deposition methods (e.g., chemical vapor deposition [CVD], atomic layer deposition [ALD], and magnetron sputtering), vacuum filtration, coating, spinning, assembly, and various polymerization methods (e.g., vapor deposition polymerization (VDP) and interfacial polymerization). For instance, CVD, ALD, and magnetron sputtering methods were used to prepare single‐layer graphene with nanopores^[^ ^261^ ^]^ or a single sub‐nanometer pore,^[^ ^221^, ^222^, ^223^ ^]^ heterogeneous Al_2_O_3_/SiO_2_ nanochannels,^[^ ^181^ ^]^ and sandwich‐like WO_3_/AAO/NiO,^[^ ^37^ ^]^ respectively. The vacuum filtration approach can be used to construct layered materials (e.g., GO,^[^ ^61^, ^262^ ^]^ C_3_N_4_,^[^ ^135^ ^]^ MXene,^[^ ^136^, ^139^ ^]^ BN,^[^ ^263^ ^]^ and kaolinite^[^ ^144^ ^]^), I/I (e.g., CaWO_4_/MnO_2_,^[^ ^68^ ^]^ GO/AAO,^[^ ^196^ ^]^ and GO/GO^[^ ^206^, ^264^, ^265^, ^266^ ^]^), O/I (e.g., SNF/AAO^[^ ^202^ ^]^ and PPSU‐Pyx/GO^[^ ^208^ ^]^), and O/O heterogeneous membranes (e.g., TYP‐CNF/(PVP/PAN)^[^ ^210^ ^]^). By using the coating method, 3D mesoporous carbon,^[^ ^165^ ^]^ I/I (e.g., TiO_2_/AAO^[^ ^67^ ^]^), O/I (e.g., BCP/AAO,^[^ ^186^ ^]^ PI/AAO,^[^ ^189^ ^]^ and MOF/AAO^[^ ^153^ ^]^), O/O (e.g., BCP/BCP,^[^ ^192^, ^193^ ^]^ BCP/PET,^[^ ^190^, ^191^, ^197^ ^]^ and PET/CP^[^ ^99^ ^]^), and O/M heterogeneous membranes (e.g., PC/AuNPs^[^ ^267^ ^]^) can be obtained. The spinning technique is generally applied to develop nanochannel‐structured fibers, including highly aligned GO gel fibers^[^ ^131^ ^]^ through wet spinning and porous carbon^[^ ^166^, ^167^ ^]^ and TiO_2_ fibers^[^ ^268^ ^]^ through electrospinning. The assembly method is also widely used in designing nanochannel‐structured membranes such as AuNPs with cone‐like nanochannels,^[^ ^269^ ^]^ HGF,^[^ ^171^ ^]^ SiO_2_ nanoparticles with 3D nanochannels,^[^ ^169^ ^]^ and 2D angstrom‐scale channels composed of graphite and BN.^[^ ^143^ ^]^ In addition, C_3_N_4_ with 1D (e.g., conical^[^ ^124^ ^]^ and cylindrical nanotubes^[^ ^121^, ^122^ ^]^) and 2D nanochannels (e.g., layered C_3_N_4_^[^ ^134^ ^]^) can be developed using a VDP approach. Polyamide,^[^ ^213^, ^214^, ^270^ ^]^ CMF,^[^ ^158^, ^159^ ^]^ O/O (e.g., PC/CP^[^ ^105^, ^106^ ^]^ and hydrogel/CP^[^ ^82^ ^]^), and O/I heterogeneous membranes (e.g., CP/AAO^[^ ^184^, ^185^, ^201^ ^]^) are usually obtained by an interfacial polymerization method. Other methods such as mechanical shearing, Stöber solution growth, and micelle templates have been used to construct MXenes with a vertical arrangement,^[^ ^40^ ^]^ SiO_2_ with perpendicular nanochannels,^[^ ^59^, ^188^, ^218^, ^219^, ^271^ ^]^ and CPs with ordered mesopores,^[^ ^231^, ^272^, ^273^ ^]^ respectively.

In this section, representative approaches for constructing nanochannels were briefly listed in terms of top‐down and bottom‐up approaches. Top‐down approaches, including various etching methods (e.g., track etching, anodization, metal nanoparticle‐assisted etching, and reactive ion etching) and chemical delignification and pyrolysis, are used to fabricate 1D and 3D nanochannels, respectively. Bottom‐up approaches, including different deposition methods (e.g., CVD, ALD, and magnetron sputtering), vacuum filtration, coating, spinning, assembly, and various polymerization methods (e.g., VDP and interfacial polymerization) are mainly used to develop various layered 2D, heterogeneous, and sandwich‐like membranes. Natural plants also provide nanochannels for ion transport. For instance, the natural ion channels in grass stems were applied to construct an electron battery after immersion in saturated salt solutions, which provides new inspiration for designing artificial membranes with nanochannels.^[^ ^41^ ^]^ The nanochannels in positively charged cellulose nanofibers consisting of chemically treated wood enabled rapid chloride ion transport.^[^ ^44^ ^]^ The 3D printing approach is being used more often to obtain membranes with multiscale structures to mediate ion transport for application in energy storage.^[^ ^138^, ^274^, ^275^, ^276^ ^]^ For example, multidimensional MXenes with internal nanochannels and external microstructures were fabricated using the 3D printing method. These multidimensional MXenes worked as high‐performance supercapacitors owing to the abundant ion transport pathways.^[^ ^138^ ^]^ Hu et al. further designed macroscale BN rods composed of vertically aligned BN nanosheets, thus exhibiting the potential of using a 3D printing approach for constructing multiscale structures.^[^ ^274^ ^]^ Superwettable interfaces (e.g., superhydrophilic and superhydrophobic interfaces) also provide efficient platforms for constructing nanochannel‐structured membranes (e.g., mixed‐dimensional nanochannels, ultrathin, and sandwich‐like membranes).^[^ ^47^, ^48^, ^277^, ^278^, ^279^ ^]^ For instance, taking advantage of the superspreading of liquids on superhydrophilic interfaces,^[^ ^49^, ^50^, ^51^ ^]^ Jiang et al. obtained membranes with controllable thickness and low roughness, which could be used to prepare uniformly ultrathin MOF and COF membranes and corresponding heterogeneous and sandwich‐like membranes. In addition, triphase interface‐mediated epitaxial growth on superhydrophobic surfaces may be used for designing these membranes efficiently because of the controlled growth rates in the lateral direction.^[^ ^52^ ^]^ With the development of preparation technology, nanochannel‐structured membranes with single‐ or mixed‐dimensional nanochannels and even specific combinations can be more precisely obtained for modulating ion transport.

## 8 APPLICATIONS OF MEMBRANES WITH UNIQUE STRUCTURES

**FIGURE 16:**
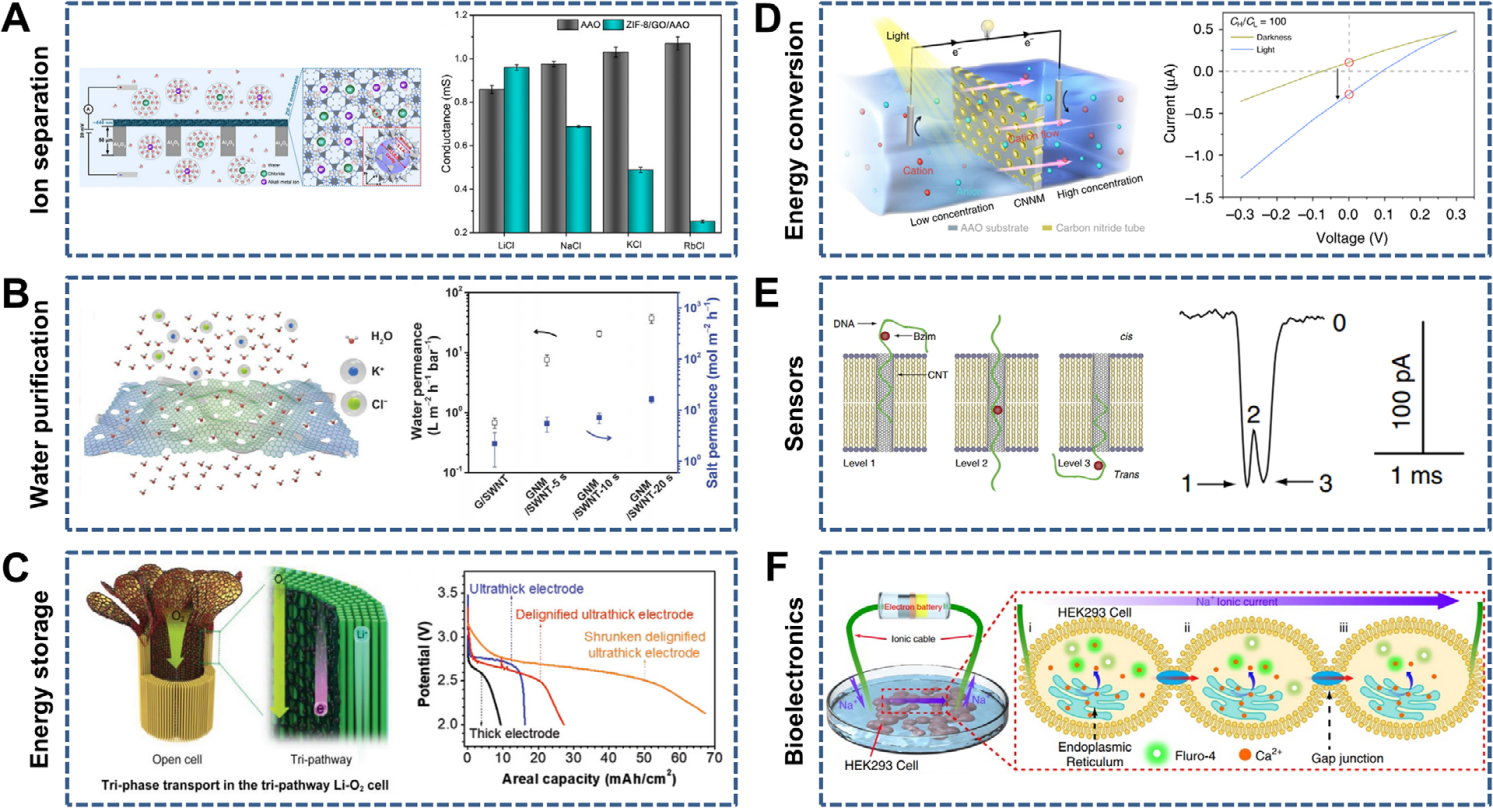
Applications of membranes with unique structures. (A) Ion transport through MOFs (left) and ion conductance of different electrolyte solutions with and without MOFs (right). Reproduced under the terms of the Creative Commons Attribution NonCommercial license.^[^ ^60^ ^]^ Copyright 2018, The Author(s). (B) Single‐layer graphene nanomesh on single‐walled CNTs (GNM/SWNT) (left) and water and salt permeance of GNM/SWNT (right). Reproduced with permission.^[^ ^287^ ^]^ Copyright 2019, American Association for the Advancement of Science. (C) Tri‐pathway lithium–oxygen batteries (left) and discharge curves of wood electrodes (right). Reproduced with permission.^[^ ^43^ ^]^ Copyright 2019, Wiley‐VCH. (D) C_3_N_4_ nanotubes for photoelectric conversion (left) and *I*–*V* curves in dark and light states (right). Reproduced under the terms of the Creative Commons CC BY license.^[^ ^121^ ^]^ Copyright 2019, The Author(s). (E) Short single‐wall CNTs for DNA2‐Bzim translocation (left) and current signature (right). Reproduced under the terms of the Creative Commons CC‐BY‐NC‐SA license.^[^ ^310^ ^]^ Copyright 2013, The Author(s). (F) Electron batteries with natural ionic cables (i.e., grass stems) for transporting ions to interact with the targeted biosystems. Reproduced under the terms of the Creative Commons CC BY license.^[^ ^41^ ^]^ Copyright 2017, The Author(s)

The ultimate goal of constructing nanochannel‐structured membranes with controllable ion transport properties is to promote corresponding applications in various fields such as ion separation, water purification, energy storage and conversion, sensors, and bioelectronics. Hence, we discuss the representative applications of these membranes with unique structures (e.g., nanochannel, ultrathin, and sandwich‐like structures) in this section (Figure 16).

### 8.1 Ion separation

Owing to the introduction of nanochannel structures in the desired membranes, selective ion separation can be achieved by mediating the nanochannel shape and size, charge, wettability, and specific recognition of the nanochannel walls. Membranes such as 1D CNTs^[^ ^114^, ^280^ ^]^ and polyhydrazide nanotubes,^[^ ^281^ ^]^ 2D layered GO,^[^ ^61^, ^282^, ^283^ ^]^ MOFs,^[^ ^60^, ^154^, ^284^ ^]^ and COFs^[^ ^58^ ^]^ consisting of interconnected and uniform pores and ultrathin^[^ ^285^ ^]^ and sandwich‐like structures^[^ ^38^ ^]^ have been used for controllable ion sieving. For instance, stacked graphene and GO membranes with sub‐nanometer interlayer spacing showed selective transport of alkali and alkaline earth cations across the membrane owing to the synergistic effect of cation–π interactions and ion dehydration.^[^ ^282^ ^]^ Recently, Wang et al. reported the rapid selective transport of alkali metal cations (selectivity of ∼4.6, for LiCl/RbCl) through MOFs (e.g., ZIF‐8) with uniform nanopores as a result of the ion selectivity of angstrom‐scale windows and the rapid ion conduction through the nanoscale cavities in the pores (Figure 16A).^[^ ^60^ ^]^ Reconstituted nanoporous COF membranes with carboxylate‐modified pore walls (diameter of 2.8 nm) exhibited excellent water permeance (∼2260 L m^−2^ h^−1^ bar^−1^) and cation selectivity.^[^ ^58^ ^]^ Notably, novel artificial water nanochannels with diameters of approximately 3 Å and a cluster structure were made of unimolecular peptide‐appended hybrid[4]arene.^[^ ^286^ ^]^ Combined with the water–wire pathways in the matrix, these artificial water nanochannels exhibited remarkable permselectivity over water/salt, which provides inspiration for constructing the next generation of selective separation membranes.

### 8.2 Water purification

Water purification is also a fundamental application of nanochannel‐structured membranes (e.g., polyamide with nanoscale Turing structures,^[^ ^270^ ^]^ graphene nanomesh,^[^ ^287^ ^]^ and hierarchical porous carbon^[^ ^288^ ^]^). Through interfacial polymerization, Gao et al. prepared polyamide membranes (thickness of ∼20 nm) with high water permeability sites provided by special Turing structures that showed superior water purification performance with a high water flux of 125 L m^−2^ h^−1^.^[^ ^270^ ^]^ Most recently, a single‐layer graphene nanomesh with high‐density sub‐nanoscale pores (size of 0.63 nm and density of ∼1.0 × 10^12^ cm^−2^) was constructed on single‐walled CNTs on a large scale (GNM/SWNT) (Figure 16B).^[^ ^287^ ^]^ The GNM/SWNT simultaneously exhibited high mechanical strength (modulus of 5–10 GPa), high water permeance (20.6 L m^−2^ h^−1^ bar^−1^), and a high salt rejection ratio (85.2%–93.4%). Hydrophilic microporous membranes consisting of sub‐nanometer pores (2–8 Å), hydrophilic functional groups (e.g., ionizable groups), and tunable thickness (∼350 nm) were designed for water purification because of their selective ion permeation.^[^ ^289^ ^]^ Taking advantage of the coordination between phytic acid and metal ions (e.g., Fe^3+^), an ultrathin membrane with the thickness of approximately 8 nm, denser nanopores, and a superhydrophilic surface was used for purifying water, showing high water permeance of 109.8 L m^−2^ h^−1^ bar^−1^.^[^ ^290^ ^]^

### 8.3 Energy storage

Membranes with 1D, 2D, and interconnected 3D nanochannels can be used for electrochemical energy storage owing to their efficient ion transport pathways. Very recently, independent tri‐pathway lithium–oxygen batteries were constructed using natural wood membranes after delignification and a CNT/Ru nanoparticle modification process (Figure 16C).^[^ ^43^ ^]^ The treated wood membranes consisted of cellulose nanofibers with abundant aligned nanochannels, which facilitated Li^+^ ion transport, whereas the CNT coating and open channels allowed transport of electrons and O_2_ gas, respectively. This unique structure contributed to the membranes’ high performance, such as ultrahigh areal capacity (67.2 mAh cm^−2^). Furthermore, horizontally aligned CNTs decorated with poly(3‐methylthiophene) were designed to enhance ion transport. The fabricated CNTs were used as supercapacitor electrodes and showed high areal capacitance (3.1 F cm^−2^) at a current density of 5 mA cm^−2^.^[^ ^291^ ^]^ Layered materials (e.g., MXenes,^[^ ^40^, ^292^ ^]^ MoS_2_,^[^ ^140^, ^141^ ^]^ and VOPO_4_^[^ ^147^ ^]^), especially with vertically aligned structures, have been used for electrochemical energy storage.^[^ ^293^, ^294^ ^]^ For example, vertically aligned MXenes showed fast electrolyte ion transport owing to their efficient pathways, which were used to construct high‐performance electrochemical devices.^[^ ^40^ ^]^ Significantly, interconnected 3D nanochannel‐structured materials, including porous carbon,^[^ ^42^, ^167^, ^172^, ^174^, ^178^, ^295^, ^296^, ^297^ ^]^ titanium dioxide,^[^ ^298^ ^]^ cellulose nanofibers,^[^ ^43^ ^]^ rGO,^[^ ^129^ ^]^ HGF,^[^ ^171^, ^299^ ^]^ GO/MXene,^[^ ^300^ ^]^ cellulose/MXene,^[^ ^301^ ^]^ and sandwich‐like PTCDA/rGO/CNT,^[^ ^229^ ^]^ are also typically used to develop electrochemical devices.^[^ ^32^, ^302^ ^]^ For example, Duan et al. reported a robust high‐performance electrode made of 3D hierarchical porous carbon/metal oxide materials, taking advantage of the accumulation of selective metal ions.^[^ ^42^ ^]^ The micro/nano channels decorated with metal oxide nanoparticles simultaneously endowed the materials with fast ion transport and mechanical stability.

### 8.4 Energy conversion

Taking advantage of adjustable ionic currents under various external stimuli, nanochannel‐structured membranes can also be used for energy conversion, including photoelectric conversion (e.g., PET with conical nanochannels,^[^ ^257^ ^]^ C_3_N_4_ nanotubes,^[^ ^121^ ^]^ TPPS nanofibers/AAO,^[^ ^303^ ^]^ and laminar WS_2_^[^ ^304^ ^]^), pressure gradient power conversion (e.g., layered BN^[^ ^263^ ^]^), thermoelectric conversion (e.g., heterogeneous nanochannel‐structured PET/SiO_2_^[^ ^188^ ^]^), and salinity gradient power generation (e.g., layered MXenes^[^ ^305^, ^306^ ^]^ and cellulose nanofibers^[^ ^307^ ^]^). Recently, Antonietti et al. reported light‐driven ion transport against a concentration gradient across C_3_N_4_ nanotubes (external diameter of ∼90 nm and inner diameter of ∼30 nm) as a result of surface charge redistribution (Figure 16D).^[^ ^121^ ^]^ When the C_3_N_4_ nanotubes were used for photoelectric conversion, the corresponding open‐circuit voltage and current density reached 550 mV and 2.4 μA cm^−2^, respectively. Electricity can be generated with an output power of 0.64 mW m^−2^ through the conversion from hydraulic pressure, taking advantage of layered BN membranes with 2D nanochannels. Furthermore, Su et al. reported the rapid thermoelectric responsiveness (sensitivity of 0.71 mV K^−1^) of heterogeneous nanochannel‐structured PET/SiO_2_ membranes, which will promote the research of thermoelectric conversion.^[^ ^188^ ^]^

### 8.5 Sensors

Nanochannel‐structured membranes have already shown great application potential in sensing external ions,^[^ ^159^, ^308^, ^309^ ^]^ molecules,^[^ ^310^, ^311^, ^312^, ^313^, ^314^, ^315^ ^]^ and stimuli.^[^ ^45^, ^241^ ^]^ For example, Wu et al. constructed nanochannels characterized by enhanced ion transport and selective detection of modified 5‐hydroxymethylcytosine in single‐stranded DNA (i.e., DNA2‐Bzim) through embedded short single‐wall CNTs in a lipid bilayer (Figure 16E).^[^ ^310^ ^]^ Asymmetric glass nanoslits (width of 70 nm) with a triangular structure were also applied for the sensitive and selective detection of troponin T.^[^ ^311^ ^]^ Temperature‐sensing PC nanochannels 50 nm in diameter were obtained by decorating a wax composite with reversible temperature‐controlled expansion and contraction, which could be used to detect the body temperature within a narrow range.^[^ ^241^ ^]^ Cellulose nanofibers with 3D interconnected nanochannels (1–2 nm) showed rapid sodium‐ion transport and could be used to construct ultrasensitive humidity sensors.^[^ ^45^ ^]^ A reversible and sensitive microsensor was prepared to monitor the real‐time pH change in a living rat brain, successfully taking advantage of transient ion transport, which provides a new approach for in vivo neurochemical recording with high spatiotemporal resolution.^[^ ^316^ ^]^

### 8.6 Bioelectronics

In nature, ion transport is ubiquitous in biological activities, such as the transmission of nerve signals and the functioning of enzymes. Significantly, the gradually rising field of bioelectronics provides an efficient way to further study real‐time ion transport in biological systems. For example, organic electrochemical transistors (OECTs) consisting of gate, source, and drain electrodes and CP layers showed large transconductance, high volumetric capacitance, long‐term mechanical stability, and good biocompatibility. They are therefore considered promising bioelectronics that can be used to stimulate nerves and record real‐time signals.^[^ ^317^, ^318^, ^319^, ^320^, ^321^, ^322^, ^323^ ^]^ This corresponding process involves both the normal ion transport from the electrolyte solutions to the CP layers and horizontal electron transport between the source and drain electrodes along the CP layers simultaneously. The representative CP layer is made of poly(3,4‐ethyle‐nedioxythiophene) doped with poly(styrenesulfonate) (PEDOT:PSS) because of its high electrical conductivity and good biocompatibility. After inducing uniform nanopores, the normal ion transport from the electrolyte solution to the PEDOT:PSS layer can be further enhanced.^[^ ^324^ ^]^ Recently, Hu et al. proposed the concept of an electron battery through which ions can be transported in external ionic cables to interact with the targeted biosystems (Figure 16F).^[^ ^41^ ^]^ More specifically, the authors constructed ionic cables by immersing grass stems in saturated salt solutions. The cables showed both stable ionic conductivity and superior mechanical strength because the interconnected channels in the stems were maintained for transporting ions in different deformed states. Furthermore, a calcium wave was observed after stimulating single‐layer living cells with a controlled ionic current provided by the electron battery. In addition, bio‐inspired synapses developed using nanochannels connected with immiscible ionic liquids and KCl solutions demonstrated voltage‐mediated movement of the interfaces, showing promising applications in neuromorphic devices.^[^ ^325^ ^]^

Most recently, Jiang et al. presented controlled ion transport related to biosystems (i.e., quantum‐confined ion superfluid^[^ ^54^, ^55^, ^56^, ^57^ ^]^), which will open a new path for studying controlled ion transport across the nanochannel‐structured membranes in both biological systems and artificial counterparts. The corresponding process is enthalpy‐driven ordered fluid in confined nanochannels without energy loss, which can explain the phenomena of nerve signal transmission. In detail, Na^+^ enter into the cell when the Na^+^ channels open, then K^+^ exit from the cell when the K^+^ channels open, leading to the rapid ion transport to achieve ultrafast neural signal transmission. This process can also be simplified by the model of the movement of multiple–single pendulums with close contact. What is more, two main approaches for investigating the quantum‐confined ion superfluid were clarified, including THz response for detecting the ions in biological nanochannels and artificial counterparts remotely.^[^ ^55^ ^]^ Taking advantage of no energy loss and highly ordered during the transport process in the confined nanochannels, quantum‐confined superfluid‐based catalysis with low reaction barrier, superior efficiency and selectivity was proposed.^[^ ^56^ ^]^ Thus, it is believed that advanced bioelectronics with ultrafast signal transmission and nanoreactors with high efficiency can be prepared with the development of membranes with unique structures.

In this section, the typical applications of nanochannel‐structured membranes were discussed in detail, including ion separation, water purification, energy storage and conversion, sensors, and bioelectronics. It is believed that membranes constructed with fine structures (e.g., nanochannels, ultrathin, and sandwich‐like structures) will pave the way for promoting corresponding applications in various fields. For instance, research on bioelectronics is gradually emerging as a hot topic as the next stage of studying controlled ion transport through nanochannel‐structured membranes. Significantly, the concept of quantum‐confined ion superfluids proposed in recent years will also promote the development of nanochannel‐structured membranes with controllable ion transport properties in both biological and artificial systems.^[^ ^54^, ^55^, ^56^, ^57^, ^326^, ^327^, ^328^ ^]^

## 9 CONCLUSION AND PERSPECTIVE

In nature, the common biological activities (e.g., uptake of ionic mineral elements, conversion of biological energy, and transmission of nerve signals) are strongly associated with the controllable transport of specific ions mediated by biological ion channel proteins across the plasma membranes, which have attracted the attention of researchers. Therefore, diverse artificial membranes with unique structures have been constructed to investigate finely tuned ion transport in artificial and biological systems, showing wide application prospects from industrial production to biological interfaces. Herein, we highlight representative nanochannel‐structured membranes with controllable ion transport characteristics from the point of view of membrane structures (e.g., nanochannel and combination structures). First, we introduced the controllability of ion transport (e.g., ion selectivity, ion gating, ion rectification, and ion storage). We then presented nanochannel‐structured membranes with single‐ and mixed‐dimensional nanochannels. Third, we discussed typical ultrathin and sandwich‐like membranes. Then, we summarized the stimulus‐responsive nanochannels in detail. We briefly listed the construction methods of nanochannel‐structured membranes and reviewed their broad applications in different fields.

The primary aims to construct membranes with different structures, and the advantages and disadvantages were discussed. The initial aims to construct 1D nanochannel‐structured membranes is to investigate the fundamental principles of ion transport taking advantage of the simplified nanochannels and precise controllability for experimental measurements and analysis in theory. However, the construction of these 1D nanochannels requires sophisticated instruments and is rather expensive and time‐consuming, which hinders the practical applications.^[^ ^27^, ^74^ ^]^ Whereas, 2D nanochannel‐structured membranes composed of stacked lamellar materials show several advantages, including facile and scalable fabrication methods (e.g., vacuum filtration), easy chemical modification in bulk solutions, adjustable channel size.^[^ ^29^ ^]^ Moreover, these membranes can transport ions vertically and horizontally, showing potential in preparing nanofluidic devices on chips. 3D nanochannel‐structured membranes have no strict limitations for raw materials and usually can be obtained in large scale via simple fabrication approaches.^[^ ^82^ ^]^ Thus, they have drawn great attention for the simplicity. Inspired by the junction channels between the adjacent cells, heterogeneous membranes are readily constructed through the simple hybridization of homogeneous membranes with different dimensions, which exhibit novel functions that differ from those of the single materials.^[^ ^33^ ^]^ However, the bulk 3D nanochannel‐structured membranes are usually thick, which can increase ion transport resistance and reduce permeation flux significantly. It is urgently required to design ultrathin membranes covered with straight and short pathways.^[^ ^36^ ^]^ Therefore, it is still a great challenge to prepare free‐standing ultrathin membranes with suitable stability and optimized permeability and selectivity. Compared to the low efficiency of modification on the inner surfaces of the targeted nanochannels, sandwich‐like membranes consisting of layers with mediated charges on the exterior surfaces of the middle nanochannels were successfully constructed via simple magnetron sputtering or electrochemical polymerization.^[^ ^37^, ^329^, ^330^ ^]^ The corresponding ion transport properties can also be finely controlled via the change of the charges on outside layers. In the practical applications, we can choose the suitable membranes with specific structures according to the controllability of ion transport and the feasibility of operation.

There is still a long way to go to construct nanochannel‐structured membranes for precisely adjusted ion transport. It is necessary to discover new raw materials to fabricate the desired membranes with specific nanochannels and search for new modifying molecules to endow the nanochannels with specific recognition and/or sensitive stimuli responsiveness. For instance, molecular machines^[^ ^331^, ^332^, ^333^ ^]^ can be further induced into nanochannel‐structured membranes to mediate ion transport properties. It is also urgently required to achieve fine membrane structures (e.g., nanochannel, ultrathin, and sandwich‐like structures) to provide efficient ion transport pathways. First, the basic geometric parameters of single‐dimensional nanochannels, including the nanochannel size, shape, and density, need to be mediated accurately and repeatedly through physical and/or chemical methods. For example, a single‐layer MoS_2_ membrane with sub‐nanometer pores^[^ ^224^ ^]^ and BN with angstrom‐scale channels^[^ ^143^ ^]^ were used to investigate the single‐ion transport behavior and voltage‐controlled ion transport, respectively. Second, mixed‐dimensional heterogeneous membranes and sandwich‐like membranes with clear and suitable combination interfaces usually allow for more precise adjustment toward ion transport, resulting in higher requirements for the construction of these membranes. Taking advantage of triphase interface‐mediated epitaxial growth on superhydrophobic surfaces,^[^ ^52^, ^334^ ^]^ these kinds of multiple nanochannel‐structured membranes can be developed owing to the controlled growth rates in the lateral direction. Third, to address the trade‐off between permeability and selectivity, ultrathin membranes with vertically arranged nanochannels (e.g., single‐layer MOFs and COFs) are ideal candidates. Through the superspreading of liquids on superwettable interfaces, membranes with controllable thickness and low roughness have been obtained,^[^ ^49^, ^50^, ^51^ ^]^ which may provide inspiration for developing uniformly ultrathin MOF and COF membranes and the corresponding heterogeneous and sandwich‐like membranes. Finally, combining external structures (e.g., geometric structures^[^ ^335^, ^336^, ^337^ ^]^ and surface macro/micro/nano structures^[^ ^39^, ^274^, ^291^ ^]^) and internal structures (e.g., nanochannel arrangement^[^ ^40^, ^294^, ^338^ ^]^) is another feasible strategy for controlling ion transport. For instance, GO membranes with asymmetric geometric structures such as non‐uniform thickness,^[^ ^335^ ^]^ Kirigami shapes,^[^ ^336^ ^]^ partial bilayers,^[^ ^337^ ^]^ anti‐T,^[^ ^39^ ^]^ and vertically aligned^[^ ^40^ ^]^ MXenes with special surface and arrangement structures showed more controllable ion transport. Recently, Hu et al. designed macroscale BN rods consisting of vertically aligned BN nanosheets through a 3D printing technique, which can be used to investigate the potential role of multiscale structures in mediating ion transport.^[^ ^274^ ^]^ Interestingly, various micro/nanostructures on biological surfaces can also be considered in the development of nanochannel‐structured membranes.^[^ ^339^ ^]^ Furthermore, natural nanochannels in organisms can be used to transport ions after special treatment. For instance, cellulose nanofibers with abundant aligned nanochannels^[^ ^44^ ^]^ in cationic wood showed rapid chloride ion transport, and hierarchical porous carbon^[^ ^178^, ^296^ ^]^ derived from natural wood functioned as high‐performance electrode materials because of their efficient ion transport pathways. In addition, natural ionic cables made of grass stems^[^ ^41^ ^]^ can transport ions to interact with biosystems in different deformed states. Thus, much effort is required to precisely construct membranes with unique nanochannels and sandwich‐like structures as well as with ultrathin thickness to mediate ion transport, which will promote applications in various fields such as ion separation, water purification, energy storage and conversion, sensors, and bioelectronics.

Notably, the concept of quantum‐confined ion superfluid was proposed for the explanation of ultrafast neural signal transmission in life system, such as the transformation of biological information in smell, vision, and audition.^[^ ^54^ ^]^ It is suggested that conventional action potential principle cannot explain the phenomena of nerve signal transmission, because large amount of energy was required for ion diffusion. On the contrary, the corresponding process is enthalpy‐driven ordered fluid in confined nanochannels without energy loss. For instance, Na^+^ enter into the cell with the open of Na^+^ channels, then K^+^ exit from the cell with the open of K^+^ channels, resulting in rapid ion transport through the membrane to achieve ultrafast neural signal transmission. This process is similar to the fast movement of multiple–single pendulums with close contact. Moreover, two approaches were given to further explore the quantum‐confined ion superfluid.^[^ ^55^ ^]^ First, THz response is a feasible way to detect the ions in the confined nanochannels in life system. Second, artificial counterparts can be adapted to investigate the THz response remotely through the controlled design of membrane structure. Beneficial from the quantum‐confined superfluid, mass (e.g., ions and molecules) transport in the confined nanochannels is a quantum way, showing no energy loss and is highly ordered. In this context, quantum‐confined superfluid‐based catalysis was also put forward to achieve reduced reaction barrier, superior efficiency, and selectivity under non‐harsh conditions.^[^ ^56^ ^]^ With the development of membranes with optimized structure, not only fundamental understanding of ultrafast signal transmission in biological system can be promoted, but also highly efficient information transformation and chemical reactions in artificial counterparts can be achieved.

## CONFLICT OF INTEREST

Lei Jiang is a member of the *Exploration* editorial board. The authors declare no conflict of interest.

## References

[1] K. Abe , K. Irie , H. Nakanishi , H. Suzuki , Y. Fujiyoshi , Nature 2018, 556, 214. 29618813 10.1038/s41586-018-0003-8

[2] Y. Cao , Y. Pan , H. Huang , X. Jin , E. J. Levin , B. Kloss , M. Zhou , Nature 2013, 496, 317. 23598339 10.1038/nature12056 PMC3726529

[3] C. M. Suomivuori , A. P. Gamiz‐Hernandez , D. Sundholm , V. R. I. Kaila , Proc. Natl. Acad. Sci. U. S. A. 2017, 114, 7043. 28611220 10.1073/pnas.1703625114 PMC5502629

[4] A. Guskov , N. Nordin , A. Reynaud , H. Engman , A. K. Lundback , A. J. Jong , T. Cornvik , T. Phua , S. Eshaghi , Proc. Natl. Acad. Sci. U. S. A. 2012, 109, 18459. 23091000 10.1073/pnas.1210076109 PMC3494898

[5] L. L. McGoldrick , A. K. Singh , K. Saotome , M. V. Yelshanskaya , E. C. Twomey , R. A. Grassucci , A. I. Sobolevsky , Nature 2018, 553, 233. 29258289 10.1038/nature25182 PMC5854407

[6] F. Ren , B. L. Logeman , X. Zhang , Y. Liu , D. J. Thiele , P. Yuan , Nat. Commun. 2019, 10, 1386. 30918258 10.1038/s41467-019-09376-7 PMC6437178

[7] E. Park , E. B. C. Ampbell , R. MacKinnon , Nature 2017, 541, 500. 28002411 10.1038/nature20812 PMC5576512

[8] B. K. Czyzewski , D. N. Wang , Nature 2012, 483, 494. 22407320 10.1038/nature10881 PMC3711795

[9] K. Kim , S. K. Kwon , S. H. Jun , J. S. Cha , H. Kim , W. Lee , J. F. Kim , H. S. Cho , Nat. Commun. 2016, 7, 12677. 27554809 10.1038/ncomms12677 PMC4999514

[10] X. J. Chen , D. G. Liu , D. H. Zhou , Y. B. Si , D. Xu , C. W. Stamatkin , M. K. Ghozayel , M. S. Ripsch , A. G. Obukhov , F. A. White , S. O. Meroueh , Proc. Natl. Acad. Sci. U. S. A. 2018, 115, E10566. 30355767 10.1073/pnas.1813157115 PMC6233087

[11] G. Maksaev , E. S. Haswell , Proc. Natl. Acad. Sci. U. S. A. 2012, 109, 19015. 23112188 10.1073/pnas.1213931109 PMC3503204

[12] S. Dang , S. Feng , J. Tien , C. J. Peters , D. Bulkley , M. Lolicato , J. Zhao , K. Zuberbuhler , W. Ye , L. Qi , T. Chen , C. S. Craik , Y. N. Jan , D. L. Minor Jr. , Y. Cheng , L. Y. Jan , Nature 2017, 552, 426. 29236684 10.1038/nature25024 PMC5750132

[13] Q. Z. Yang , P. Kuzyk , I. Antonov , C. J. Bostwick , A. B. Kohn , L. L. Moroz , R. D. Hawkins , Proc. Natl. Acad. Sci. U. S. A. 2015, 112, 16030. 26668355 10.1073/pnas.1501731113 PMC4702976

[14] C. Speziale , L. S. Manni , C. Manatschal , E. M. Landau , R. Mezzenga , Proc. Natl. Acad. Sci. U. S. A. 2016, 113, 7491. 27313210 10.1073/pnas.1603965113 PMC4941478

[15] M. Malvezzi , M. Chalat , R. Janjusevic , A. Picollo , H. Terashima , A. K. Menon , A. Accardi , Nat. Commun. 2013, 4, 2367. 23996062 10.1038/ncomms3367 PMC3970400

[16] M. Queralt‐Martin , M. L. Lopez , M. Aguilella‐Arzo , V. M. Aguilella , A. Alcaraz , Nano Lett. 2018, 18, 6604. 30178677 10.1021/acs.nanolett.8b03235 PMC6242701

[17] B. Cressiot , S. J. Greive , W. Si , T. C. Pascoa , M. Mojtabavi , M. Chechik , H. T. Jenkins , X. G. Lu , K. Zhang , A. Alcsimentiev , A. A. Antson , M. Wanunu , ACS Nano 2017, 11, 11931. 29120602 10.1021/acsnano.7b06980 PMC5963890

[18] S. Borsley , S. L. Cockroft , ACS Nano 2018, 12, 786. 29244946 10.1021/acsnano.7b08105

[19] O. B. Tarun , M. Y. Eremchev , A. Radenovic , S. Roke , Nano Lett. 2019, 19, 7608. 31580677 10.1021/acs.nanolett.9b02024

[20] S. Shekar , C. C. Chien , A. Hartel , P. Ong , O. B. Clarke , A. Marks , M. Drndic , K. L. Shepard , Nano Lett. 2019, 19, 1090. 30601669 10.1021/acs.nanolett.8b04388 PMC6904930

[21] G. Grauwels , H. Valkenier , A. P. Davis , I. Jabin , K. Bartik , Angew. Chem. Int. Ed. 2019, 58, 6921. 10.1002/anie.201900818 30925004

[22] Y. C. Yao , A. Taqieddin , M. A. Alibakhshi , M. Wanunu , N. R. Aluru , A. Noy , ACS Nano 2019, 13, 12851. 31682401 10.1021/acsnano.9b05118

[23] S. M. N. Uddin , S. Laokroekkiat , M. A. Rashed , S. Mizuno , K. Ono , M. Ishizaki , K. Kanaizuka , M. Kurihara , Y. Nagao , T. Hamada , Chem. Commun. 2019, 56, 1046. 10.1039/c9cc06153c 31868183

[24] Y. Sun , J. Ma , F. Zhang , F. Zhu , Y. Mei , L. Liu , D. Tian , H. Li , Nat. Commun. 2017, 8, 260. 28811463 10.1038/s41467-017-00330-z PMC5558008

[25] K. Raidongia , J. Huang , J. Am. Chem. Soc. 2012, 134, 16528. 22998077 10.1021/ja308167f

[26] X. Li , H. Zhang , P. Wang , J. Hou , J. Lu , C. D. Easton , X. Zhang , M. R. Hill , A. W. Thornton , J. Z. Liu , B. D. Freeman , A. J. Hill , L. Jiang , H. Wang , Nat. Commun. 2019, 10, 2490. 31186413 10.1038/s41467-019-10420-9 PMC6560108

[27] H. C. Zhang , Y. Tian , L. Jiang , Nano Today 2016, 11, 61.

[28] Z. P. Zhu , D. Y. Wang , Y. Tian , L. Jiang , J. Am. Chem. Soc. 2019, 141, 8658. 31063693 10.1021/jacs.9b00086

[29] J. Gao , Y. Feng , W. Guo , L. Jiang , Chem. Soc. Rev. 2017, 46, 5400. 28722059 10.1039/c7cs00369b

[30] Y. Kang , Y. Xia , H. T. Wang , X. W. Zhang , Adv. Funct. Mater. 2019, 29, 1902014.

[31] S. Kim , H. Wang , Y. M. Lee , Angew. Chem. Int. Ed. 2019, 58, 17512. 10.1002/anie.201814349 PMC6900107 30811730

[32] H. T. Sun , J. Zhu , D. Baumann , L. L. Peng , Y. X. Xu , I. Shakir , Y. Huang , X. F. Duan , Nat. Rev. Mater. 2019, 4, 45.

[33] Z. Zhang , L. P. Wen , L. Jiang , Chem. Soc. Rev. 2018, 47, 322. 29300401 10.1039/c7cs00688h

[34] R. Li , X. Fan , Z. Liu , J. Zhai , Adv. Mater. 2017, 29, 1702983. 10.1002/adma.201702983 28833604

[35] D. Pakulski , W. Czepa , S. D. Buffa , A. Ciesielski , P. Samorì , Adv. Funct. Mater. 2019, 30, 1902394.

[36] C. Zhang , B.‐H. Wu , M.‐Q. Ma , Z. Wang , Z.‐K. Xu , Chem. Soc. Rev. 2019, 48, 3811. 31179451 10.1039/c9cs00322c

[37] Q. Q. Zhang , Q. R. Liu , J. X. Kang , Q. J. Huang , Z. Y. Liu , X. G. Diao , J. Zhai , Adv. Sci. 2018, 5, 1800163. 10.1002/advs.201800163 PMC6145424 30250783

[38] Q. Ji , X. An , H. Liu , L. Guo , J. Qu , ACS Nano 2015, 9, 10922. 26481603 10.1021/acsnano.5b04027

[39] M. Lu , W. J. Han , H. J. Li , H. B. Li , B. S. Zhang , W. Zhang , W. T. Zheng , Adv. Mater. Interfaces 2019, 6, 1900160.

[40] Y. Xia , T. S. Mathis , M. Q. Zhao , B. Anasori , A. Dang , Z. Zhou , H. Cho , Y. Gogotsi , S. Yang , Nature 2018, 557, 409. 29769673 10.1038/s41586-018-0109-z

[41] C. Wang , K. K. Fu , J. Dai , S. D. Lacey , Y. Yao , G. Pastel , L. Xu , J. Zhang , L. Hu , Nat. Commun. 2017, 8, 15609. 28737174 10.1038/ncomms15609 PMC5527283

[42] J. Zhu , Y. Shan , T. Wang , H. Sun , Z. Zhao , L. Mei , Z. Fan , Z. Xu , I. Shakir , Y. Huang , B. Lu , X. Duan , Nat. Commun. 2016, 7, 13432. 27853174 10.1038/ncomms13432 PMC5118540

[43] C. J. Chen , S. M. Xu , Y. D. Kuang , W. T. Gan , J. W. Song , G. G. Chen , G. Pastel , B. Y. Liu , Y. J. Li , H. Huang , L. B. Hu , Adv. Energy Mater. 2019, 9, 1802964.

[44] G. G. Chen , T. Li , C. J. Chen , C. W. Wang , Y. Liu , W. Q. Kong , D. P. Liu , B. Jiang , S. M. He , Y. D. Kuang , L. B. Hu , Adv. Funct. Mater. 2019, 29, 1902772.

[45] C. W. Wang , S. Wang , G. Chen , W. Q. Kong , W. W. Ping , J. Q. Dai , G. Pastel , H. Xie , S. M. He , S. Das , L. B. Hu , Chem. Mater. 2018, 30, 7707.

[46] W. Kong , C. Wang , C. Jia , Y. Kuang , G. Pastel , C. Chen , G. Chen , S. He , H. Huang , J. Zhang , S. Wang , L. Hu , Adv. Mater. 2018, 30, 1801934. 10.1002/adma.201801934 30101467

[47] M. J. Liu , S. T. Wang , L. Jiang , Nat. Rev. Mater. 2017, 2, 17036.

[48] Y. Wu , J. Feng , H. Gao , X. Feng , L. Jiang , Adv. Mater. 2019, 31, 1800718. 10.1002/adma.201800718 30592333

[49] P. C. Zhang , F. L. Zhang , C. Q. Zhao , S. T. Wang , M. J. Liu , L. Jiang , Angew. Chem. Int. Ed. 2016, 55, 3615. 10.1002/anie.201510291 26880685

[50] Z. Zhu , Y. Tian , Y. Chen , Z. Gu , S. Wang , L. Jiang , Angew. Chem. Int. Ed. 2017, 56, 5720. 10.1002/anie.201700039 28370912

[51] Z. Zhu , S. Zheng , S. Peng , Y. Zhao , Y. Tian , Adv. Mater. 2017, 29, 1703120. 10.1002/adma.201703120 29024052

[52] Y. P. Chen , J. X. Meng , Z. P. Zhu , F. L. Zhang , L. Y. Wang , Z. Gu , L. Jiang , S. T. Wang , Adv. Funct. Mater. 2018, 28, 1800240.

[53] Y. Chen , Z. Zhu , X. Jiang , L. Jiang , Adv. Sci. 2021, 8, 2100949. 10.1002/advs.202100949 PMC8425917 34245121

[54] X. Q. Zhang , L. Jiang , Nano Res. 2019, 12, 1219.

[55] X. Q. Zhang , M. Antonietti , L. Jiang , Sci. China Mater. 2020, 63, 167.

[56] Y. W. Hao , X. Q. Zhang , L. Jiang , Nanoscale Horiz. 2019, 4, 1029.

[57] X. Zhang , H. Liu , L. Jiang , Adv. Mater. 2019, 31, 1804508. 10.1002/adma.201804508 30345614

[58] V. A. Kuehl , J. Yin , P. H. H. Duong , B. Mastorovich , B. Newell , K. D. Li‐Oakey , B. A. Parkinson , J. O. Hoberg , J. Am. Chem. Soc. 2018, 140, 18200. 30512941 10.1021/jacs.8b11482

[59] X. Lin , Q. Yang , L. Ding , B. Su , ACS Nano 2015, 9, 11266. 26458217 10.1021/acsnano.5b04887

[60] H. Zhang , J. Hou , Y. Hu , P. Wang , R. Ou , L. Jiang , J. Z. Liu , B. D. Freeman , A. J. Hill , H. Wang , Sci. Adv. 2018, 4, eaaq0066. 29487910 10.1126/sciadv.aaq0066 PMC5817922

[61] J. Abraham , K. S. Vasu , C. D. Williams , K. Gopinadhan , Y. Su , C. T. Cherian , J. Dix , E. Prestat , S. J. Haigh , I. V. Grigorieva , P. Carbone , A. K. Geim , R. R. Nair , Nat. Nanotechnol. 2017, 12, 546. 28369049 10.1038/nnano.2017.21

[62] A. Esfandiar , B. Radha , F. C. Wang , Q. Yang , S. Hu , S. Garaj , R. R. Nair , A. K. Geim , K. Gopinadhan , Science 2017, 358, 511. 29074772 10.1126/science.aan5275

[63] C. Lian , H. P. Su , C. Z. Li , H. L. Liu , J. Z. Wu , ACS Nano 2019, 13, 8185. 31251573 10.1021/acsnano.9b03303

[64] Z. Q. Li , Y. Wang , Z. Q. Wu , M. Y. Wu , X. H. Xia , J. Phys. Chem. C 2019, 123, 13687.

[65] X. M. Wang , Y. Chen , Z. Y. Meng , Q. Q. Zhang , J. Zhai , J. Phys. Chem. C 2018, 122, 24863.

[66] H. C. Yang , Y. Xie , J. Hou , A. K. Cheetham , V. Chen , S. B. Darling , Adv. Mater. 2018, 30, 1801495. 10.1002/adma.201801495 30028547

[67] Q. Q. Zhang , Z. Y. Hu , Z. Y. Liu , J. Zhai , L. Jiang , Adv. Funct. Mater. 2014, 24, 424.

[68] J. Zhang , Y. Yang , Z. Zhang , P. Wang , X. Wang , Adv. Mater. 2014, 26, 1071. 24282127 10.1002/adma.201304270

[69] X. Hou , W. Guo , F. Xia , F. Q. Nie , H. Dong , Y. Tian , L. P. Wen , L. Wang , L. X. Cao , Y. Yang , J. M. Xue , Y. L. Song , Y. G. Wang , D. S. Liu , L. Jiang , J. Am. Chem. Soc. 2009, 131, 7800. 19435350 10.1021/ja901574c

[70] G. Perez‐Mitta , A. G. Albesa , W. Knoll , C. Trautmann , M. E. Toimil‐Molares , O. Azzaroni , Nanoscale 2015, 7, 15594. 26365392 10.1039/c5nr04645a

[71] Q. Liu , L. P. Wen , K. Xiao , H. Lu , Z. Zhang , G. H. Xie , X. Y. Kong , Z. S. Bo , L. Jiang , Adv. Mater. 2016, 28, 3181. 26917448 10.1002/adma.201505250

[72] Q. Liu , K. Xiao , L. P. Wen , Y. Dong , G. H. Xie , Z. Zhang , Z. S. Bo , L. Jiang , ACS Nano 2014, 8, 12292. 25482729 10.1021/nn506257c

[73] B. V. V. S. P. Kumar , K. P. Sonu , K. V. Rao , S. Sampath , S. J. George , M. Eswaramoorthy , ACS Appl. Mater. Interfaces 2018, 10, 23458. 29975507 10.1021/acsami.8b07098

[74] X. Hou , W. Guo , L. Jiang , Chem. Soc. Rev. 2011, 40, 2385. 21308139 10.1039/c0cs00053a

[75] H. Zhang , Y. Tian , J. Hou , X. Hou , G. Hou , R. Ou , H. Wang , L. Jiang , ACS Nano 2015, 9, 12264. 26474219 10.1021/acsnano.5b05542

[76] L. Shi , C. L. Mu , T. Gao , W. X. Chai , A. Z. Sheng , T. S. Chen , J. Yang , X. L. Zhu , G. X. Li , J. Am. Chem. Soc. 2019, 141, 8239. 31050413 10.1021/jacs.9b01759

[77] J. X. Quan , F. Zhu , M. K. Dhinakaran , Y. Y. Yang , R. P. Johnson , H. B. Li , Angew. Chem. Int. Ed. 2021, 60, 2892. 10.1002/anie.202012984 33145896

[78] C. Cheng , G. Jiang , G. P. Simon , J. Z. Liu , D. Li , Nat. Nanotechnol. 2018, 13, 685. 29967459 10.1038/s41565-018-0181-4

[79] G. Hou , D. Wang , K. Xiao , H. Zhang , S. Zheng , P. Li , Y. Tian , L. Jiang , Small 2018, 14, 1703369. 10.1002/smll.201703369 29399965

[80] M. Y. Liu , H. C. Zhang , K. Li , L. P. Heng , S. T. Wang , Y. Tian , L. Jiang , Adv. Funct. Mater. 2015, 25, 421.

[81] Z. W. Wang , X. Fan , Q. Q. Wang , S. N. Hou , H. M. Wang , J. Zhai , X. M. Meng , RSC Adv. 2016, 6, 63652.

[82] B. Bao , J. Hao , X. Bian , X. Zhu , K. Xiao , J. Liao , J. Zhou , Y. Zhou , L. Jiang , Adv. Mater. 2017, 29, 1702926. 10.1002/adma.201702926 29024293

[83] C. Zhao , J. Lu , J. Hou , X. Y. Li , J. Wang , L. Jiang , H. T. Wang , H. C. Zhang , Adv. Funct. Mater. 2019, 29, 1806416.

[84] J. Wang , J. Hou , H. C. Zhang , Y. Tian , L. Jiang , ACS Appl. Mater. Interfaces 2018, 10, 2033. 29266925 10.1021/acsami.7b16539

[85] K. Xiao , L. Chen , Z. Zhang , G. Xie , P. Li , X. Y. Kong , L. Wen , L. Jiang , Angew. Chem. Int. Ed. 2017, 56, 8168. 10.1002/anie.201704137 28544087

[86] E. Madrid , Y. Y. Rong , M. Carta , N. B. McKeown , R. Malpass‐Evans , G. A. Attard , T. J. Clarke , S. H. Taylor , Y. T. Long , F. Marken , Angew. Chem. Int. Ed. 2014, 53, 10751. 10.1002/anie.201405755 25113137

[87] T.‐W. Lin , J.‐P. Hsu , C.‐Y. Lin , S. Tseng , J. Phys. Chem. C 2019, 123, 12437.

[88] W. J. Lan , D. A. Holden , H. S. White , J. Am. Chem. Soc. 2011, 133, 13300. 21800889 10.1021/ja205773a

[89] L. X. Cao , W. Guo , Y. G. Wang , L. Jiang , Langmuir 2012, 28, 2194. 22148901 10.1021/la203837q

[90] C. Y. Lin , E. T. Acar , J. W. Polster , K. Lin , J. P. Hsu , Z. S. Siwy , ACS Nano 2019, 13, 9868. 31348640 10.1021/acsnano.9b01357

[91] S. Park , G. Yossifon , Nanoscale 2018, 10, 11633. 29896609 10.1039/c8nr02389a

[92] L. Peng , Z. Fang , Y. Zhu , C. Yan , G. Yu , Adv. Energy Mater. 2018, 8, 1702179.

[93] Q. Zhang , X. Li , Y. Chen , Q. Zhang , H. Liu , J. Zhai , X. Yang , Adv. Mater. 2017, 29, 1606871. 10.1002/adma.201606871 28436059

[94] M. Ali , P. Ramirez , H. Q. Nguyen , S. Nasir , J. Cervera , S. Mafe , W. Ensinger , ACS Nano 2012, 6, 3631. 22458890 10.1021/nn3010119

[95] H. Zhang , X. Hou , L. Zeng , F. Yang , L. Li , D. Yan , Y. Tian , L. Jiang , J. Am. Chem. Soc. 2013, 135, 16102. 23773031 10.1021/ja4037669

[96] J. Wang , R. Fang , J. Hou , H. Zhang , Y. Tian , H. Wang , L. Jiang , ACS Nano 2017, 11, 3022. 28226213 10.1021/acsnano.6b08727

[97] K. Xiao , G. Xie , Z. Zhang , X. Y. Kong , Q. Liu , P. Li , L. Wen , L. Jiang , Adv. Mater. 2016, 28, 3345. 26928676 10.1002/adma.201505842

[98] H. C. Zhang , X. Hou , J. Hou , L. Zeng , Y. Tian , L. Li , L. Jiang , Adv. Funct. Mater. 2015, 25, 1102.

[99] G. Perez‐Mitta , W. A. Marmisolle , C. Trautmann , M. E. Toimil‐Molares , O. Azzaroni , Adv. Mater. 2017, 29, 1700972. 10.1002/adma.201700972 28516507

[100] K. Xiao , K. Wu , L. Chen , X. Y. Kong , Y. Zhang , L. Wen , L. Jiang , Angew. Chem. Int. Ed. 2018, 57, 151. 10.1002/anie.201708695 29139188

[101] Y. Wu , G. Yang , M. Lin , X. Kong , L. Mi , S. Liu , G. Chen , Y. Tian , L. Jiang , Angew. Chem. Int. Ed. 2019, 58, 12481. 10.1002/anie.201906360 31317609

[102] Y. Wu , D. Wang , I. Willner , Y. Tian , L. Jiang , Angew. Chem. Int. Ed. 2018, 57, 7790. 10.1002/anie.201803222 29687555

[103] Y. N. Li , G. H. Du , G. B. Mao , J. L. Guo , J. Zhao , R. Q. Wu , W. J. Liu , ACS Appl. Mater. Interfaces 2019, 11, 38055. 31553570 10.1021/acsami.9b13088

[104] Z. Siwy , D. Dobrev , R. Neumann , C. Trautmann , K. Voss , Appl. Phys. A 2003, 76, 781.

[105] G. Perez‐Mitta , W. A. Marmisolle , C. Trautmann , M. E. Toimil‐Molares , O. Azzaroni , J. Am. Chem. Soc. 2015, 137, 15382. 26587977 10.1021/jacs.5b10692

[106] G. Perez‐Mitta , W. A. Marmisolle , L. Burr , M. E. Toimil‐Molares , C. Trautmann , O. Azzaroni , Small 2018, 14, 1703144. 10.1002/smll.201703144 29399954

[107] H. Mamad‐Hemouch , L. Bacri , C. Huin , C. Przybylski , B. Thiebot , G. Patriarche , N. Jarroux , J. Pelta , Nanoscale 2018, 10, 15303. 30069556 10.1039/c8nr02623h

[108] H. Mamad‐Hemouch , H. Ramoul , M. A. Taha , L. Bacri , C. Huin , C. Przybylski , A. Oukhaled , B. Thiebot , G. Patriarche , N. Jarroux , J. Pelta , Nano Lett. 2015, 15, 7748. 26471761 10.1021/acs.nanolett.5b03938

[109] Q. Xu , S. Tao , Q. Jiang , D. Jiang , J. Am. Chem. Soc. 2018, 140, 7429. 29807426 10.1021/jacs.8b03814

[110] C. Y. Li , F. X. Ma , Z. Q. Wu , H. L. Gao , W. T. Shao , K. Wang , X. H. Xia , Adv. Funct. Mater. 2013, 23, 3836.

[111] Q. Lu , Q. Tang , Z. Chen , S. Zhao , G. Qing , T. Sun , ACS Appl. Mater. Interfaces 2017, 9, 32554. 28871777 10.1021/acsami.7b09992

[112] J. Jung , J. Kim , H. S. Lee , I. S. Kang , K. Choi , ACS Nano 2019, 13, 10761. 31482707 10.1021/acsnano.9b05570

[113] D. Zhang , Q. Wang , X. Fan , M. Zhang , J. Zhai , L. Jiang , Adv. Mater. 2018, 30, 1804862. 10.1002/adma.201804862 30284330

[114] X. Gong , J. Li , K. Xu , J. Wang , H. Yang , J. Am. Chem. Soc. 2010, 132, 1873. 20102186 10.1021/ja905753p

[115] T. James , Y. V. Kalinin , C. C. Chan , J. S. Randhawa , M. Gaevski , D. H. Gracias , Nano Lett. 2012, 12, 3437. 22725714 10.1021/nl300673r PMC3491980

[116] B. V. Kumar , K. V. Rao , S. Sampath , S. J. George , M. Eswaramoorthy , Angew. Chem. Int. Ed. 2014, 53, 13073. 10.1002/anie.201406448 25256699

[117] J. Hwang , S. Kataoka , A. Endo , H. Daiguji , Lab Chip 2016, 16, 3824. 27714018 10.1039/c6lc00844e

[118] R. Fan , S. Huh , R. Yan , J. Arnold , P. Yang , Nat. Mater. 2008, 7, 303. 18297076 10.1038/nmat2127

[119] H. Daiguji , J. Hwang , A. Takahashi , S. Kataoka , A. Endo , Langmuir 2012, 28, 3671. 22242888 10.1021/la204477h

[120] J. Hwang , H. Daiguji , Langmuir 2013, 29, 2406. 23368973 10.1021/la304423p

[121] K. Xiao , L. Chen , R. Chen , T. Heil , S. D. C. Lemus , F. Fan , L. Wen , L. Jiang , M. Antonietti , Nat. Commun. 2019, 10, 74. 30622279 10.1038/s41467-018-08029-5 PMC6325115

[122] K. Xiao , B. Kumru , L. Chen , L. Jiang , B. Schmidt , M. Antonietti , Beilstein J. Nanotechnol. 2019, 10, 1316. 31293868 10.3762/bjnano.10.130 PMC6604718

[123] K. Xiao , L. Chen , L. Jiang , M. Antonietti , Nano Energy 2020, 67, 104230.

[124] K. Xiao , B. Tu , L. Chen , T. Heil , L. Wen , L. Jiang , M. Antonietti , Angew. Chem. Int. Ed. 2019, 58, 12574. 10.1002/anie.201907833 PMC6790565 31294908

[125] J. Gao , X. L. Liu , Y. N. Jiang , L. P. Ding , L. Jiang , W. Guo , Small 2019, 15, 1804279. 10.1002/smll.201804279 30653272

[126] Z. Zhang , X. Huang , Y. Qian , W. Chen , L. Wen , L. Jiang , Adv. Mater. 2019, 32, 1904351. 10.1002/adma.201904351 31793736

[127] J. Zhang , Z. Li , K. Zhan , R. Sun , Z. Sheng , M. Wang , S. Wang , X. Hou , Electrophoresis 2019, 40, 2029. 30968445 10.1002/elps.201800529

[128] S. Kim , J. Choi , C. Choi , J. Heo , D. W. Kim , J. Y. Lee , Y. T. Hong , H. T. Jung , H. T. Kim , Nano Lett. 2018, 18, 3962. 29723474 10.1021/acs.nanolett.8b01429

[129] H. Banda , S. Perie , B. Daffos , P. L. Taberna , L. Dubois , O. Crosnier , P. Simon , D. Lee , G. De Paepe , F. Duclairoir , ACS Nano 2019, 13, 1443. 30642165 10.1021/acsnano.8b07102 PMC6961951

[130] J. C. Liu , L. J. Yu , G. C. Yue , N. Wang , Z. M. Cui , L. L. Hou , J. H. Li , Q. Z. Li , A. Karton , Q. F. Cheng , L. Jiang , Y. Zhao , Adv. Funct. Mater. 2019, 29, 1808501.

[131] H. Park , K. H. Lee , Y. B. Kim , S. B. Ambade , S. H. Noh , W. Eom , J. Y. Hwang , W. J. Lee , J. Huang , T. H. Han , Sci. Adv. 2018, 4, eaau2104. 30406202 10.1126/sciadv.aau2104 PMC6214641

[132] Y. Liang , F. Zhao , Z. H. Cheng , Q. H. Zhou , H. B. Shao , L. Jiang , L. T. Qu , Nano Energy 2017, 32, 329.

[133] L. Chen , G. Shi , J. Shen , B. Peng , B. Zhang , Y. Wang , F. Bian , J. Wang , D. Li , Z. Qian , G. Xu , G. Liu , J. Zeng , L. Zhang , Y. Yang , G. Zhou , M. Wu , W. Jin , J. Li , H. Fang , Nature 2017, 550, 380. 28992630 10.1038/nature24044

[134] K. Xiao , P. Giusto , L. Wen , L. Jiang , M. Antonietti , Angew. Chem. Int. Ed. 2018, 57, 10123. 10.1002/anie.201804299 29939454

[135] Y. Wang , N. N. Wu , Y. Wang , H. Ma , J. X. Zhang , L. L. Xu , M. K. Albolkany , B. Liu , Nat. Commun. 2019, 10, 2500. 31175298 10.1038/s41467-019-10381-z PMC6555826

[136] Z. Lu , Y. Wei , J. Deng , L. Ding , Z. K. Li , H. Wang , ACS Nano 2019, 13, 10535. 31480834 10.1021/acsnano.9b04612

[137] C. H. Yang , Y. Tang , Y. P. Tian , Y. Y. Luo , Y. C. He , X. T. Yin , W. X. Que , Adv. Funct. Mater. 2018, 28, 1705487.

[138] W. Yang , J. Yang , J. J. Byun , F. P. Moissinac , J. Xu , S. J. Haigh , M. Domingos , M. A. Bissett , R. A. W. Dryfe , S. Barg , Adv. Mater. 2019, 31, 1902725. 10.1002/adma.201902725 31343084

[139] J. Lao , R. Lv , J. Gao , A. Wang , J. Wu , J. Luo , ACS Nano 2018, 12, 12464. 30495925 10.1021/acsnano.8b06708

[140] T. Xiang , Q. Fang , H. Xie , C. Wu , C. Wang , Y. Zhou , D. Liu , S. Chen , A. Khalil , S. Tao , Q. Liu , L. Song , Nanoscale 2017, 9, 6975. 28524923 10.1039/c7nr02003a

[141] Y. Xu , F. Bahmani , M. Zhou , Y. L. Li , C. L. Zhang , F. Liang , S. H. Kazemi , U. Kaiser , G. W. Meng , Y. Lei , Nanoscale Horiz. 2019, 4, 202. 32254157 10.1039/c8nh00305j

[142] Y. C. Jiao , A. Mukhopadhyay , Y. Ma , L. Yang , A. M. Hafez , H. L. Zhu , Adv. Energy Mater. 2018, 8, 1702779.

[143] T. Mouterde , A. Keerthi , A. R. Poggioli , S. A. Dar , A. Siria , A. K. Geim , L. Bocquet , B. Radha , Nature 2019, 567, 87. 30842639 10.1038/s41586-019-0961-5

[144] H. Cheng , Y. Zhou , Y. Feng , W. Geng , Q. Liu , W. Guo , L. Jiang , Adv. Mater. 2017, 29, 1700177. 10.1002/adma.201700177 28397411

[145] T. L. Xiao , Q. Q. Liu , Q. Q. Zhang , Z. Y. Liu , J. Zhai , J. Phys. Chem. C 2017, 121, 18954.

[146] Y. Zhou , H. Ding , A. T. Smith , X. H. Jia , S. Chen , L. Liu , S. E. Chavez , Z. L. Hou , J. J. Liu , H. F. Cheng , Q. F. Liu , L. Y. Sun , J. Mater. Chem. A 2019, 7, 14089.

[147] L. Peng , Y. Zhu , X. Peng , Z. Fang , W. Chu , Y. Wang , Y. Xie , Y. Li , J. J. Cha , G. Yu , Nano Lett. 2017, 17, 6273. 28873318 10.1021/acs.nanolett.7b02958

[148] Y. B. Zhou , C. J. Chen , X. Zhang , D. P. Liu , L. S. Xu , J. Q. Dai , S. C. Liou , Y. L. Wang , C. Li , H. Xie , Q. Y. Wu , B. Foster , T. Li , R. M. Briber , L. B. Hu , J. Am. Chem. Soc. 2019, 141, 17830. 31647658 10.1021/jacs.9b09009

[149] T. Cherian , D. R. Nunes , T. G. Dane , J. Jacquemin , U. Vainio , T. T. T. Myllymaki , J. V. I. Timonen , N. Houbenov , M. Marechal , P. Rannou , O. Ikkala , Adv. Funct. Mater. 2019, 29, 1905054.

[150] J. Luo , Y. Li , H. Zhang , A. Wang , W. S. Lo , Q. Dong , N. Wong , C. Povinelli , Y. Shao , S. Chereddy , S. Wunder , U. Mohanty , C. K. Tsung , D. Wang , Angew. Chem. Int. Ed. 2019, 58, 15313. 10.1002/anie.201908706 31478284

[151] L. Gao , K. Y. Chan , C. V. Li , L. Xie , J. F. Olorunyomi , Nano Lett. 2019, 19, 4990. 31322897 10.1021/acs.nanolett.9b01211

[152] Y. Guo , M. Sun , H. Liang , W. Ying , X. Zeng , Y. Ying , S. Zhou , C. Liang , Z. Lin , X. Peng , ACS Appl. Mater. Interfaces 2018, 10, 30451. 30117730 10.1021/acsami.8b11042

[153] R. R. Li , J. Q. Jiang , Q. Q. Liu , Z. Q. Xie , J. Zhai , Nano Energy 2018, 53, 643.

[154] Y. Guo , Y. Ying , Y. Mao , X. Peng , B. Chen , Angew. Chem. Int. Ed. 2016, 55, 15120. 10.1002/anie.201607329 27805300

[155] Z. Guo , Y. Zhang , Y. Dong , J. Li , S. Li , P. Shao , X. Feng , B. Wang , J. Am. Chem. Soc. 2019, 141, 1923. 30657664 10.1021/jacs.8b13551

[156] J. Hou , H. Zhang , G. P. Simon , H. Wang , Adv. Mater. 2020, 32, 1902009. 10.1002/adma.201902009 31273835

[157] R. Dong , P. Han , H. Arora , M. Ballabio , M. Karakus , Z. Zhang , C. Shekhar , P. Adler , P. S. Petkov , A. Erbe , S. C. B. Mannsfeld , C. Felser , T. Heine , M. Bonn , X. Feng , E. Canovas , Nat. Mater. 2018, 17, 1027. 30323335 10.1038/s41563-018-0189-z

[158] X. He , H. Sin , B. Liang , Z. A. Ghazi , A. M. Khattak , N. A. Khan , H. R. Alanagh , L. S. Li , X. Q. Lu , Z. Y. Tang , Adv. Funct. Mater. 2019, 29, 1900134.

[159] C. Gu , N. Huang , J. Gao , F. Xu , Y. Xu , D. Jiang , Angew. Chem. Int. Ed. 2014, 53, 4850. 10.1002/anie.201402141 24692401

[160] Q. Q. Liu , Z. Tang , M. D. Wu , Z. H. Zhou , Polym. Int. 2014, 63, 381.

[161] Y. He , Y. Yang , X. X. Ji , Q. C. Zhang , T. Jiang , H. C. Shi , S. F. Luan , R. K. Y. Li , D. Shi , J. Membr. Sci. 2019, 572, 358.

[162] C. Jeon , J. J. Han , M. Seo , ACS Appl. Mater. Interfaces 2018, 10, 40854. 30384592 10.1021/acsami.8b14712

[163] J. H. Kim , J. H. Kim , K. H. Choi , H. K. Yu , J. H. Kim , J. S. Lee , S. Y. Lee , Nano Lett. 2014, 14, 4438. 24979037 10.1021/nl5014037

[164] S. P. Surwade , S. H. Chai , J. P. Choi , X. Wang , J. S. Lee , I. V. Vlassiouk , S. M. Mahurin , S. Dai , Langmuir 2014, 30, 3606. 24655006 10.1021/la404669m

[165] D. Feng , Y. Y. Lv , Z. X. Wu , Y. Q. Dou , L. Han , Z. K. Sun , Y. Y. Xia , G. F. Zheng , D. Y. Zhao , J. Am. Chem. Soc. 2011, 133, 15148. 21854032 10.1021/ja2056227

[166] T. Liu , Z. Zhou , Y. Guo , D. Guo , G. Liu , Nat. Commun. 2019, 10, 675. 30737399 10.1038/s41467-019-08644-w PMC6368586

[167] Y. Sun , R. B. Sills , X. Hu , Z. W. Seh , X. Xiao , H. Xu , W. Luo , H. Jin , Y. Xin , T. Li , Z. Zhang , J. Zhou , W. Cai , Y. Huang , Y. Cui , Nano Lett. 2015, 15, 3899. 26011653 10.1021/acs.nanolett.5b00738

[168] Y. Eygeris , E. V. White , Q. Wang , J. E. Carpenter , M. Grunwald , I. Zharov , ACS Appl. Mater. Interfaces 2019, 11, 3407. 30589251 10.1021/acsami.8b17483

[169] E. Choi , K. Kwon , D. Kim , J. Park , Lab Chip 2015, 15, 512. 25407418 10.1039/c4lc00949e

[170] C. Buelke , A. Alshami , J. Casler , Y. Lin , M. Hickner , I. H. Aljundi , J. Membr. Sci. 2019, 588, 117195.

[171] Y. Xu , Z. Lin , X. Zhong , X. Huang , N. O. Weiss , Y. Huang , X. Duan , Nat. Commun. 2014, 5, 4554. 25105994 10.1038/ncomms5554

[172] K. Jayaramulu , D. P. Dubal , B. Nagar , V. Ranc , O. Tomanec , M. Petr , K. K. R. Datta , R. Zboril , P. Gomez‐Romero , R. A. Fischer , Adv. Mater. 2018, 30, 1705789. 10.1002/adma.201705789 29516561

[173] B. Ding , J. Wang , Y. Wang , Z. Chang , G. Pang , H. Dou , X. Zhang , Nanoscale 2016, 8, 11136. 27181616 10.1039/c6nr02155g

[174] X. M. Fan , C. Yu , J. Yang , Z. Ling , C. Hu , M. D. Zhang , J. S. Qiu , Adv. Energy Mater. 2015, 5, 1401761.

[175] J. Wu , J. Peng , Z. Yu , Y. Zhou , Y. Guo , Z. Li , Y. Lin , K. Ruan , C. Wu , Y. Xie , J. Am. Chem. Soc. 2018, 140, 493. 29202228 10.1021/jacs.7b11915

[176] X. Zhang , R. Lv , A. Wang , W. Guo , X. Liu , J. Luo , Angew. Chem. Int. Ed. 2018, 57, 15028. 10.1002/anie.201808714 30199139

[177] Z. Ma , X. Zhou , W. Deng , D. Lei , Z. Liu , ACS Appl. Mater. Interfaces 2018, 10, 3634. 29297670 10.1021/acsami.7b17386

[178] X. W. Peng , L. Zhang , Z. X. Chen , L. X. Zhong , D. K. Zhao , X. Chi , X. X. Zhao , L. G. Li , X. H. Lu , K. Leng , C. B. Liu , W. Liu , W. Tang , K. P. Loh , Adv. Mater. 2019, 31, 1900341.

[179] S. Wu , F. Wildhaber , O. Vazquez‐Mena , A. Bertsch , J. Brugger , P. Renaud , Nanoscale 2012, 4, 5718. 22885910 10.1039/c2nr31243c

[180] L. J. Cheng , L. J. Guo , ACS Nano 2009, 3, 575. 19220010 10.1021/nn8007542

[181] R. X. Yan , W. J. Liang , R. Fan , P. D. Yang , Nano Lett. 2009, 9, 3820. 19603791 10.1021/nl9020123

[182] L. J. Small , D. R. Wheeler , E. D. Spoerke , Nanoscale 2015, 7, 16909. 26411335 10.1039/c5nr02939b

[183] J. Cai , W. Ma , L. Xu , C. Hao , M. Sun , X. Wu , F. M. Colombari , A. F. de Moura , M. C. Silva , E. B. Carneiro‐Neto , E. Chaves Pereira , H. Kuang , C. Xu , Angew. Chem. Int. Ed. 2019, 58, 17418. 10.1002/anie.201909447 31603286

[184] Q. Q. Zhang , Z. Y. Liu , K. F. Wang , J. Zhai , Adv. Funct. Mater. 2015, 25, 2091.

[185] Q. Q. Zhang , Z. Zhang , H. J. Zhou , Z. Q. Xie , L. P. Wen , Z. Y. Liu , J. Zhai , X. G. Diao , Nano Res. 2017, 10, 3715.

[186] X. Sui , Z. Zhang , Z. Zhang , Z. Wang , C. Li , H. Yuan , L. Gao , L. Wen , X. Fan , L. Yang , X. Zhang , L. Jiang , Angew. Chem. Int. Ed. 2016, 55, 13056. 10.1002/anie.201606469 27651002

[187] Z. Zhang , X. Y. Kong , K. Xiao , G. Xie , Q. Liu , Y. Tian , H. Zhang , J. Ma , L. Wen , L. Jiang , Adv. Mater. 2016, 28, 144. 26551055 10.1002/adma.201503668

[188] K. Chen , L. Yao , B. Su , J. Am. Chem. Soc. 2019, 141, 8608. 31067855 10.1021/jacs.9b03569

[189] K. Han , L. Heng , L. Wen , L. Jiang , Nanoscale 2016, 8, 12318. 27270836 10.1039/c6nr02506d

[190] Z. Zhang , P. Li , X. Y. Kong , G. Xie , Y. Qian , Z. Wang , Y. Tian , L. Wen , L. Jiang , J. Am. Chem. Soc. 2018, 140, 1083. 29261309 10.1021/jacs.7b11472

[191] Z. Zhang , X. Y. Kong , K. Xiao , Q. Liu , G. Xie , P. Li , J. Ma , Y. Tian , L. Wen , L. Jiang , J. Am. Chem. Soc. 2015, 137, 14765. 26535954 10.1021/jacs.5b09918

[192] Z. Zhang , X. Sui , P. Li , G. Xie , X. Y. Kong , K. Xiao , L. Gao , L. Wen , L. Jiang , J. Am. Chem. Soc. 2017, 139, 8905. 28602079 10.1021/jacs.7b02794

[193] L. Wen , K. Xiao , A. V. Sainath , M. Komura , X. Y. Kong , G. Xie , Z. Zhang , Y. Tian , T. Iyoda , L. Jiang , Adv. Mater. 2016, 28, 757. 26630640 10.1002/adma.201504960

[194] Z. Meng , Y. Chen , X. Li , Y. Xu , J. Zhai , ACS Appl. Mater. Interfaces 2015, 7, 7709. 25806828 10.1021/acsami.5b00647

[195] L. Zeng , Z. Yang , H. Zhang , X. Hou , Y. Tian , F. Yang , J. Zhou , L. Li , L. Jiang , Small 2014, 10, 793. 24031024 10.1002/smll.201301647

[196] D. Y. Ji , Q. Wen , L. X. Cao , Q. Kang , S. J. Lin , X. P. Zhang , L. Jiang , W. Guo , Adv. Mater. Technol. 2019, 4, 1800742.

[197] Z. Zhang , G. Xie , K. Xiao , X. Y. Kong , P. Li , Y. Tian , L. Wen , L. Jiang , Adv. Mater. 2016, 28, 9613. 27629083 10.1002/adma.201602758

[198] T. L. Xiao , J. Ma , J. Q. Jiang , M. K. Gan , B. X. Lu , R. F. Luo , Q. Q. Liu , Q. Q. Zhang , Z. Y. Liu , J. Zhai , Chem.‐Eur. J. 2019, 25, 12795.31376182

[199] J. Gao , W. Guo , D. Feng , H. Wang , D. Zhao , L. Jiang , J. Am. Chem. Soc. 2014, 136, 12265. 25137214 10.1021/ja503692z

[200] C. Wang , F. F. Liu , Z. Tan , Y. M. Chen , W. C. Hu , X. H. Xia , Adv. Funct. Mater. 2019, 30, 1908804.

[201] Q. Zhang , J. Kang , Z. Xie , X. Diao , Z. Liu , J. Zhai , Adv. Mater. 2018, 30, 1703323. 10.1002/adma.201703323 29215141

[202] W. Xin , Z. Zhang , X. Huang , Y. Hu , T. Zhou , C. Zhu , X. Y. Kong , L. Jiang , L. Wen , Nat. Commun. 2019, 10, 3876. 31462636 10.1038/s41467-019-11792-8 PMC6713777

[203] L. Wang , Y. Feng , Y. Zhou , M. Jia , G. Wang , W. Guo , L. Jiang , Chem. Sci. 2017, 8, 4381. 28660062 10.1039/c7sc00153c PMC5472846

[204] L. L. Wang , Q. Wen , P. Jia , M. J. Jia , D. N. Lu , X. M. Sun , L. Jiang , W. Guo , Adv. Mater. 2019, 31, 1903029. 10.1002/adma.201903029 31339197

[205] W. W. Fei , M. M. Xue , H. Qiu , W. L. Guo , Nanoscale 2019, 11, 1313. 30604817 10.1039/c8nr07557c

[206] X. Zhang , Q. Wen , L. Wang , L. Ding , J. Yang , D. Ji , Y. Zhang , L. Jiang , W. Guo , ACS Nano 2019, 13, 4238. 30865824 10.1021/acsnano.8b09285

[207] Y. P. Feng , L. P. Ding , D. Y. Ji , L. L. Wang , W. Guo , Chin. Chem. Lett. 2018, 29, 892.

[208] X. Zhu , Y. Zhou , J. Hao , B. Bao , X. Bian , X. Jiang , J. Pang , H. Zhang , Z. Jiang , L. Jiang , ACS Nano 2017, 11, 10816. 29039923 10.1021/acsnano.7b03576

[209] Z. Zhang , L. He , C. Zhu , Y. Qian , L. Wen , L. Jiang , Nat. Commun. 2020, 11, 875. 32054863 10.1038/s41467-020-14674-6 PMC7018769

[210] J. H. Kim , M. Gu , H. Lee do , J. H. Kim , Y. S. Oh , S. H. Min , B. S. Kim , S. Y. Lee , Nano Lett. 2016, 16, 5533. 27383666 10.1021/acs.nanolett.6b02069

[211] H. B. Park , J. Kamcev , L. M. Robeson , M. Elimelech , B. D. Freeman , Science 2017, 356, 307. 28619885 10.1126/science.aab0530

[212] Q. Zhang , P. S. Cao , Y. Cheng , S. S. Yang , Y. D. Yin , T. Y. Lv , Z. Y. Gu , Adv. Funct. Mater. 2020, 30, 2004854.

[213] Z. Jiang , S. Karan , A. G. Livingston , Adv. Mater. 2018, 30, 1705973. 10.1002/adma.201705973 29484724

[214] S. Gao , Y. Zhu , Y. Gong , Z. Wang , W. Fang , J. Jin , ACS Nano 2019, 13, 5278. 31017384 10.1021/acsnano.8b09761

[215] M. Y. Wu , J. Q. Yuan , H. Wu , Y. L. Su , H. Yang , X. D. You , R. N. Zhang , X. Y. He , N. A. Khan , R. Kasher , Z. Y. Jiang , J. Membrane Sci. 2019, 576, 131.

[216] J. Yuan , M. Wu , H. Wu , Y. Liu , X. You , R. Zhang , Y. Su , H. Yang , J. Shen , Z. Jiang , J. Mater. Chem. A 2019, 7, 25641.

[217] Y. Yang , P. Dementyev , N. Biere , D. Emmrich , P. Stohmann , R. Korzetz , X. Zhang , A. Beyer , S. Koch , D. Anselmetti , A. Golzhauser , ACS Nano 2018, 12, 4695. 29741359 10.1021/acsnano.8b01266

[218] W. H. Wu , Q. Yang , B. Su , J. Membr. Sci. 2018, 558, 86.

[219] Q. Yang , B. Su , Y. F. Wang , W. H. Wu , Electrophoresis 2019, 40, 2149. 30916400 10.1002/elps.201800533

[220] Y. Fu , A. V. Rudnev , G. K. H. Wiberg , M. Arenz , Angew. Chem. Int. Ed. 2017, 56, 12883. 10.1002/anie.201705952 28763143

[221] H. Yao , J. Zeng , P. Zhai , Z. Li , Y. Cheng , J. Liu , D. Mo , J. Duan , L. Wang , Y. Sun , J. Liu , ACS Appl. Mater. Interfaces 2017, 9, 11000. 28262018 10.1021/acsami.6b16736

[222] T. Jain , B. C. Rasera , R. J. Guerrero , M. S. Boutilier , S. C. O'Hern , J. C. Idrobo , R. Karnik , Nat. Nanotechnol. 2015, 10, 1053. 26436566 10.1038/nnano.2015.222

[223] S. Sahu , M. Di Ventra , M. Zwolak , Nano Lett. 2017, 17, 4719. 28678508 10.1021/acs.nanolett.7b01399 PMC5614503

[224] J. Feng , K. Liu , M. Graf , D. Dumcenco , A. Kis , M. Di Ventra , A. Radenovic , Nat. Mater. 2016, 15, 850. 27019385 10.1038/nmat4607

[225] J. P. Thiruraman , K. Fujisawa , G. Danda , P. M. Das , T. Zhang , A. Bolotsky , N. Perea‐Lopez , A. Nicolai , P. Senet , M. Terrones , M. Drndic , Nano Lett. 2018, 18, 1651. 29464959 10.1021/acs.nanolett.7b04526

[226] P. M. Das , J. P. Thiruraman , Y. C. Chou , G. Danda , M. Drndic , Nano Lett. 2019, 19, 392. 30532980 10.1021/acs.nanolett.8b04155

[227] X. Davoy , A. Gelle , J. C. Lebreton , H. Tabuteau , A. Soldera , A. Szymczyk , A. Ghoufi , ACS Omega 2018, 3, 6305. 31458812 10.1021/acsomega.8b01076 PMC6644522

[228] L. Mogg , S. Zhang , G. P. Hao , K. Gopinadhan , D. Barry , B. L. Liu , H. M. Cheng , A. K. Geim , M. Lozada‐Hidalgo , Nat. Commun. 2019, 10, 4243. 31534140 10.1038/s41467-019-12314-2 PMC6751181

[229] G. Y. Zhou , Y. E. Miao , Z. X. Wei , L. L. Mo , F. L. Lai , Y. Wu , J. M. Ma , T. X. Liu , Adv. Funct. Mater. 2018, 28, 1804629.

[230] G. He , M. Xu , J. Zhao , S. Jiang , S. Wang , Z. Li , X. He , T. Huang , M. Cao , H. Wu , M. D. Guiver , Z. Jiang , Adv. Mater. 2017, 29, 1605898. 10.1002/adma.201605898 28585367

[231] H. Tian , J. Q. Qin , D. Hou , Q. Li , C. Li , Z. S. Wu , Y. Y. Mai , Angew. Chem. Int. Ed. 2019, 58, 10173. 10.1002/anie.201903684 31140216

[232] L. Wang , T. Wei , L. Z. Sheng , L. L. Jiang , X. L. Wu , Q. H. Zhou , B. Yuan , J. M. Yue , Z. Liu , Z. J. Fan , Nano Energy 2016, 30, 84.

[233] M. Ali , P. Ramirez , S. Mafe , R. Neumann , W. Ensinger , ACS Nano 2009, 3, 603. 19222230 10.1021/nn900039f

[234] B. Yameen , M. Ali , R. Neumann , W. Ensinger , W. Knoll , O. Azzaroni , Chem. Commun. 2010, 46, 1908. 10.1039/b920870d 20198249

[235] Y. C. Qian , Z. Zhang , W. Tian , L. P. Wen , L. Jiang , Faraday Discuss. 2018, 210, 101. 29972197 10.1039/c8fd00025e

[236] S. Chen , S. Zhang , C. Bao , C. Wang , Q. Lin , L. Zhu , Chem. Commun. 2016, 52, 13132. 10.1039/c6cc07792g 27761537

[237] M. Ali , I. Ahmed , S. Nasir , P. Ramirez , C. M. Niemeyer , S. Mafe , W. Ensinger , ACS Appl. Mater. Interfaces 2015, 7, 19541. 26310320 10.1021/acsami.5b06015

[238] M. Ali , Q. H. Nguyen , R. Neumann , W. Ensinger , Chem. Commun. 2010, 46, 6690. 10.1039/c0cc01632b 20737089

[239] J. Yang , X. Hu , X. Kong , P. Jia , D. Ji , D. Quan , L. Wang , Q. Wen , D. Lu , J. Wu , L. Jiang , W. Guo , Nat. Commun. 2019, 10, 1171. 30862778 10.1038/s41467-019-09178-x PMC6414642

[240] Y. L. Xu , B. X. Lu , L. L. Fu , J. Zhai , Electrochim. Acta 2019, 316, 266.

[241] K. Y. Chun , W. Choi , S. C. Roh , C. S. Han , Nanoscale 2015, 7, 12427. 26130272 10.1039/c5nr02743h

[242] W. Guo , H. W. Xia , F. Xia , X. Hou , L. X. Cao , L. Wang , J. M. Xue , G. Z. Zhang , Y. L. Song , D. B. Zhu , Y. G. Wang , L. Jiang , ChemPhysChem 2010, 11, 859. 20140936 10.1002/cphc.200900989

[243] J. Yang , W. Zhu , X. Zhang , F. Chen , L. Jiang , New J. Chem. 2019, 43, 7190.

[244] Y. Xue , Y. Xia , S. Yang , Y. Alsaid , K. Y. Fong , Y. Wang , X. Zhang , Science 2021, 372, 501. 33926952 10.1126/science.abb5144

[245] Y. Q. Wang , H. C. Zhang , Y. Kang , Y. L. Zhu , G. P. Simon , H. T. Wang , ACS Nano 2019, 13, 11793. 31526000 10.1021/acsnano.9b05758

[246] N. Van Toan , N. Inomata , M. Toda , T. Ono , Nanotechnology 2018, 29, 195301. 29473829 10.1088/1361-6528/aab1d3

[247] D. Wang , S. Zheng , H. Liu , J. Tang , W. Miao , H. Wang , Y. Tian , H. Yang , L. Jiang , Adv. Mater. 2019, 31, 1805953. 10.1002/adma.201805953 30549326

[248] L. J. Lang , Z. S. Zhang , J. W. Shen , X. Y. Liu , J. Phys. Chem. C 2017, 121, 19512.

[249] M. Wang , H. Meng , D. Wang , Y. Yin , P. Stroeve , Y. Zhang , Z. Sheng , B. Chen , K. Zhan , X. Hou , Adv. Mater. 2019, 31, 1805130. 10.1002/adma.201805130 30633407

[250] S. Sahu , J. Elenewski , C. Rohmann , M. Zwolak , Sci. Adv. 2019, 5, eaaw5478. 31309155 10.1126/sciadv.aaw5478 PMC6625819

[251] A. Fang , K. Kroenlein , D. Riccardi , A. Smolyanitsky , Nat. Mater. 2019, 18, 76. 30478453 10.1038/s41563-018-0220-4

[252] A. Fang , K. Kroenlein , A. Smolyanitsky , J. Phys. Chem. C 2019, 123, 3588.

[253] W. Guo , H. W. Xia , L. X. Cao , F. Xia , S. T. Wang , G. Z. Zhang , Y. L. Song , Y. G. Wang , L. Jiang , D. B. Zhu , Adv. Funct. Mater. 2010, 20, 3561.

[254] Z. Wang , S. Zhang , Y. Chen , Z. Zhang , S. Ma , Chem. Soc. Rev. 2020, 49, 708. 31993598 10.1039/c9cs00827f

[255] S. Wang , L. Yang , G. He , B. Shi , Y. Li , H. Wu , R. Zhang , S. Nunes , Z. Jiang , Chem. Soc. Rev. 2020, 49, 1071. 31971530 10.1039/c9cs00751b

[256] M. Ali , P. Ramirez , M. N. Tahir , S. Mafe , Z. Siwy , R. Neumann , W. Tremel , W. Ensinger , Nanoscale 2011, 3, 1894. 21423941 10.1039/c1nr00003a

[257] Z. Meng , H. Bao , J. Wang , C. Jiang , M. Zhang , J. Zhai , L. Jiang , Adv. Mater. 2014, 26, 2329. 24347524 10.1002/adma.201304755

[258] H. Ren , T. Xiao , Q. Zhang , Z. Liu , Chem. Commun. 2018, 54, 12310. 10.1039/c8cc06076b 30272063

[259] M. Tsutsui , S. Hongo , Y. H. He , M. Taniguchi , N. Gemma , T. Kawai , ACS Nano 2012, 6, 3499. 22424475 10.1021/nn300530b

[260] M. Tsutsui , Y. He , K. Yokota , A. Arima , S. Hongo , M. Taniguchi , T. Washio , T. Kawai , ACS Nano 2016, 10, 803. 26641133 10.1021/acsnano.5b05906

[261] M. Caglar , I. Silkina , B. T. Brown , A. L. Thorneywork , O. J. Burton , V. Babenko , S. M. Gilbert , A. Zettl , S. Hofmann , U. F. Keyser , ACS Nano 2020, 14, 2729. 31891480 10.1021/acsnano.9b08168 PMC7098055

[262] W. Guo , C. Cheng , Y. Wu , Y. Jiang , J. Gao , D. Li , L. Jiang , Adv. Mater. 2013, 25, 6064. 23900945 10.1002/adma.201302441

[263] S. Qin , D. Liu , Y. Chen , C. Chen , G. Wang , J. M. Wang , J. M. Razal , W. W. Lei , Nano Energy 2018, 47, 368.

[264] X. P. Zhang , M. J. Jia , L. L. Wang , P. Jia , L. Jiang , W. Guo , Phys. Status Solidi‐R. 2019, 13, 1900129.

[265] J. Z. Ji , Q. Kang , Y. Zhou , Y. P. Feng , X. Chen , J. Y. Yuan , W. Guo , Y. Wei , L. Jiang , Adv. Funct. Mater. 2017, 27, 1603623.

[266] W. Fei , M. Xue , H. Qiu , W. Guo , Nanoscale 2019, 11, 1313. 30604817 10.1039/c8nr07557c

[267] E. Barry , S. P. McBride , H. M. Jaeger , X. M. Lin , Nat. Commun. 2014, 5, 5847. 25517763 10.1038/ncomms6847

[268] N. Wang , Y. Gao , Y. X. Wang , K. Liu , W. Lai , Y. Hu , Y. Zhao , S. L. Chou , L. Jiang , Adv. Sci. 2016, 3, 1600013. 10.1002/advs.201600013 PMC5074262 27818908

[269] S. Rao , K. J. Si , L. W. Yap , Y. Xiang , W. Cheng , ACS Nano 2015, 9, 11218. 26486960 10.1021/acsnano.5b04784

[270] Z. Tan , S. Chen , X. Peng , L. Zhang , C. Gao , Science 2018, 360, 518. 29724951 10.1126/science.aar6308

[271] Z. G. Teng , G. F. Zheng , Y. Q. Dou , W. Li , C. Y. Mou , X. H. Zhang , A. M. Asiri , D. Y. Zhao , Angew. Chem. Int. Ed. 2012, 51, 2173. 10.1002/anie.201108748 22271504

[272] S. H. Liu , P. Gordiichuk , Z. S. Wu , Z. Y. Liu , W. Wei , M. Wagner , N. Mohamed‐Noriega , D. Q. Wu , Y. Y. Mai , A. Herrmann , K. Mullen , X. L. Feng , Nat. Commun. 2015, 6, 8817. 26577914 10.1038/ncomms9817 PMC4660032

[273] S. Liu , J. Zhang , R. Dong , P. Gordiichuk , T. Zhang , X. Zhuang , Y. Mai , F. Liu , A. Herrmann , X. Feng , Angew. Chem. Int. Ed. 2016, 55, 12516. 10.1002/anie.201606988 27603275

[274] Z. Q. Liang , Y. Pei , C. J. Chen , B. Jiang , Y. G. Yao , H. Xie , M. L. Jiao , G. G. Chen , T. Y. Li , B. Yang , L. B. Hu , ACS Nano 2019, 13, 12653. 31584264 10.1021/acsnano.9b04202

[275] J. X. Zhao , Y. Zhang , X. X. Zhao , R. T. Wang , J. X. Xie , C. F. Yang , J. J. Wang , Q. C. Zhang , L. L. Li , C. H. Lu , Y. G. Yao , Adv. Funct. Mater. 2019, 29, 1900809.

[276] Z. Fan , C. Wei , L. Yu , Z. Xia , J. Cai , Z. Tian , G. Zou , S. X. Dou , J. Sun , ACS Nano 2020, 14, 867. 31898892 10.1021/acsnano.9b08030

[277] Y. Chen , J. Meng , Z. Zhu , F. Zhang , L. Wang , Z. Gu , S. Wang , Langmuir 2018, 34, 6063. 29737857 10.1021/acs.langmuir.8b01061

[278] Z. P. Zhu , Z. W. Yu , F. F. Yun , D. Pan , Y. Tian , L. Jiang , X. L. Wang , Natl. Sci. Rev. 2021, 8, nwaa166. 34691554 10.1093/nsr/nwaa166 PMC8288373

[279] Z. Zhu , Y. Chen , Z. Xu , Z. Yu , X. Luo , J. Zhou , Y. Tian , L. Jiang , iScience 2021, 24, 102334. 33855283 10.1016/j.isci.2021.102334 PMC8027538

[280] S. F. Buchsbaum , M. L. Jue , A. M. Sawvel , C. T. Chen , E. R. Meshot , S. J. Park , M. Wood , K. J. Wu , C. L. Bilodeau , F. Aydin , T. A. Pham , E. Y. Lau , F. Fornasiero , Adv. Sci. 2021, 8, 2001802. 10.1002/advs.202001802 PMC7856893 33552850

[281] A. Roy , H. Joshi , R. Ye , J. Shen , F. Chen , A. Aksimentiev , H. Zeng , Angew. Chem. Int. Ed. 2020, 59, 4806. 10.1002/anie.201916287 PMC7093082 31950583

[282] P. Sun , F. Zheng , M. Zhu , Z. Song , K. Wang , M. Zhong , D. Wu , R. B. Little , Z. Xu , H. Zhu , ACS Nano 2014, 8, 850. 24401025 10.1021/nn4055682

[283] M. C. Zhang , P. X. Zhao , P. S. Li , Y. F. Ji , G. P. Liu , W. Q. Jin , ACS Nano 2021, 15, 5209. 33621056 10.1021/acsnano.0c10451

[284] R. Li , B. Lu , Z. Xie , J. Zhai , Small 2019, 15, 1904866. 10.1002/smll.201904866 31778019

[285] Y. Yang , R. Hillmann , Y. Qi , R. Korzetz , N. Biere , D. Emmrich , M. Westphal , B. Buker , A. Hutten , A. Beyer , D. Anselmetti , A. Golzhauser , Adv. Mater. 2020, 32, 1907850. 10.1002/adma.201907850 31945240

[286] W. Song , H. Joshi , R. Chowdhury , J. S. Najem , Y. X. Shen , C. Lang , C. B. Henderson , Y. M. Tu , M. Farell , M. E. Pitz , C. D. Maranas , P. S. Cremer , R. J. Hickey , S. A. Sarles , J. L. Hou , A. Aksimentiev , M. Kumar , Nat. Nanotechnol. 2019, 15, 73. 31844288 10.1038/s41565-019-0586-8 PMC7008941

[287] Y. Yang , X. Yang , L. Liang , Y. Gao , H. Cheng , X. Li , M. Zou , R. Ma , Q. Yuan , X. Duan , Science 2019, 364, 1057. 31197007 10.1126/science.aau5321

[288] S. J. Yang , T. Kim , K. Lee , Y. S. Kim , J. Yoon , C. R. Park , Carbon 2014, 71, 294.

[289] R. Tan , A. Wang , R. Malpass‐Evans , R. Williams , E. W. Zhao , T. Liu , C. Ye , X. Zhou , B. P. Darwich , Z. Fan , L. Turcani , E. Jackson , L. Chen , S. Y. Chong , T. Li , K. E. Jelfs , A. I. Cooper , N. P. Brandon , C. P. Grey , N. B. McKeown , Q. Song , Nat. Mater. 2019, 19, 195. 31792424 10.1038/s41563-019-0536-8

[290] X. You , H. Wu , R. Zhang , Y. Su , L. Cao , Q. Yu , J. Yuan , K. Xiao , M. He , Z. Jiang , Nat. Commun. 2019, 10, 4160. 31519877 10.1038/s41467-019-12100-0 PMC6744495

[291] Y. Zhou , X. X. Wang , L. Acauan , E. Kalfon‐Cohen , X. C. Ni , Y. Stein , K. K. Gleason , B. L. Wardle , Adv. Mater. 2019, 31, 1901916. 10.1002/adma.201901916 31157472

[292] Z. Cao , Q. Zhu , S. Wang , D. Zhang , H. Chen , Z. Du , B. Li , S. Yang , Adv. Funct. Mater. 2019, 30, 1908075.

[293] Q. Yun , L. Li , Z. Hu , Q. Lu , B. Chen , H. Zhang , Adv. Mater. 2019, 32, 1903826. 10.1002/adma.201903826 31566269

[294] X. Wang , T. Wang , J. Borovilas , X. He , S. Du , Y. Yang , Nano Res. 2019, 12, 2002.

[295] J. H. Yu , C. Yu , W. Guo , Z. Wang , S. F. Li , J. W. Chang , X. Y. Tan , Y. W. Ding , M. D. Zhang , L. Yang , Y. Y. Xie , R. Fu , J. S. Qiu , Nano Energy 2019, 64, 103921.

[296] Y. Q. Li , Y. X. Lu , Q. S. Meng , A. C. S. Jensen , Q. Q. Zhang , Q. H. Zhang , Y. X. Tong , Y. R. Qi , L. Gu , M. M. Titirici , Y. S. Hu , Adv. Energy. Mater. 2019, 9, 1902852.

[297] Q. D. Nguyen , J. Patra , C. T. Hsieh , J. L. Li , Q. F. Dong , J. K. Chang , ChemSusChem 2019, 12, 449. 30548119 10.1002/cssc.201802489

[298] G. H. Lee , J. K. Kang , Adv. Sci. 2020, 7, 1902986. 10.1002/advs.201902986 PMC7080513 32195098

[299] J. H. Jeong , G. W. Lee , Y. H. Kim , Y. J. Choi , K. C. Roh , K. B. Kim , Chem. Eng. J. 2019, 378, 122126.

[300] Z. Fan , Y. Wang , Z. Xie , D. Wang , Y. Yuan , H. Kang , B. Su , Z. Cheng , Y. Liu , Adv. Sci. 2018, 5, 1800750. 10.1002/advs.201800750 PMC6193160 30356956

[301] Y. M. Wang , X. Wang , X. L. Li , Y. Bai , H. H. Xiao , Y. Liu , R. Liu , G. H. Yuan , Adv. Funct. Mater. 2019, 29, 1900326.

[302] W. Li , S. Rao , Y. Xiao , Z. Gao , Y. Chen , H. Wang , M. Ouyang , iScience 2021, 24, 102401. 33997686 10.1016/j.isci.2021.102401 PMC8102908

[303] D. Zhang , S. Zhou , Y. Liu , X. Fan , M. Zhang , J. Zhai , L. Jiang , ACS Nano 2018, 12, 11169. 30376291 10.1021/acsnano.8b05695

[304] P. Jia , Q. Wen , D. Liu , M. Zhou , X. Jin , L. Ding , H. Dong , D. Lu , L. Jiang , W. Guo , Small 2019, 15, 1905355. 10.1002/smll.201905355 31714020

[305] S. Hong , F. Ming , Y. Shi , R. Li , I. S. Kim , C. Y. Tang , H. N. Alshareef , P. Wang , ACS Nano 2019, 13, 8917. 31305989 10.1021/acsnano.9b02579

[306] L. Ding , D. Xiao , Z. Lu , J. Deng , Y. Wei , J. Caro , H. Wang , Angew. Chem. Int. Ed. 2020, 59, 8720. 10.1002/anie.201915993 31950586

[307] Q. Y. Wu , C. W. Wang , R. L. Wang , C. J. Chen , J. L. Gao , J. Q. Dai , D. P. Liu , Z. W. Lin , L. B. Hu , Adv. Energy. Mater. 2019, 10, 1902590.

[308] J. H. Bae , D. C. Wang , K. K. Hu , M. V. Mirkin , Anal. Chem. 2019, 91, 5530. 30977642 10.1021/acs.analchem.9b00426

[309] Y. L. Tang , L. X. Cao , K. Zhan , Y. Xie , D. H. Sun , X. Hou , S. Y. Chen , Sens. Actuator B‐Chem. 2019, 286, 315.

[310] L. Liu , C. Yang , K. Zhao , J. Li , H. C. Wu , Nat. Commun. 2013, 4, 2989. 24352224 10.1038/ncomms3989 PMC3905707

[311] Y. Liu , L. Yobas , Nano Lett. 2014, 14, 6983. 25366228 10.1021/nl5032524

[312] J. Wang , J. Hou , H. Zhang , Y. Tian , L. Jiang , ACS Appl. Mater. Interfaces 2018, 10, 2033. 29266925 10.1021/acsami.7b16539

[313] K. Wu , X. Y. Kong , K. Xiao , Y. Wei , C. C. Zhu , R. Zhou , M. T. Si , J. J. Wang , Y. Q. Zhang , L. P. Wen , Adv. Funct. Mater. 2019, 29, 1807953.

[314] P. Tripathi , L. Shuai , H. Joshi , H. Yamazaki , W. H. Fowle , A. Aksimentiev , H. Fenniri , M. Wanunu , J. Am. Chem. Soc. 2020, 142, 1680. 31913034 10.1021/jacs.9b10993 PMC7175996

[315] Q. Y. Ouyang , L. Tu , Y. Zhang , H. Chen , Y. F. Fan , Y. F. Tu , Y. Y. Li , Y. Sun , Anal. Chem. 2020, 92, 14947. 33119273 10.1021/acs.analchem.0c02424

[316] K. Zhang , H. Wei , T. Xiong , Y. Jiang , W. Ma , F. Wu , P. Yu , L. Mao , Chem. Sci. 2021, 12, 7369. 34163826 10.1039/d1sc00061f PMC8171349

[317] M. Moser , J. F. Ponder , A. Wadsworth , A. Giovannitti , I. McCulloch , Adv. Funct. Mater. 2018, 29, 1807033.

[318] M. Berggren , X. Crispin , S. Fabiano , M. P. Jonsson , D. T. Simon , E. Stavrinidou , K. Tybrandt , I. Zozoulenko , Adv. Mater. 2019, 31, 1805813. 10.1002/adma.201805813 30620417

[319] E. Zeglio , O. Inganas , Adv. Mater. 2018, 30, 1800941. 10.1002/adma.201800941 30022545

[320] Y. Kim , A. Chortos , W. T. Xu , Y. X. Liu , J. Y. Oh , D. Son , J. Kang , A. M. Foudeh , C. X. Zhu , Y. Lee , S. M. Niu , J. Liu , R. Pfattner , Z. N. Bao , T. W. Lee , Science 2018, 360, 998. 29853682 10.1126/science.aao0098

[321] W. Lee , D. Kim , N. Matsuhisa , M. Nagase , M. Sekino , G. G. Malliaras , T. Yokota , T. Someya , Proc. Natl. Acad. Sci. U. S. A. 2017, 114, 10554. 28923928 10.1073/pnas.1703886114 PMC5635873

[322] J. Rivnay , S. Inal , A. Salleo , R. M. Owens , M. Berggren , G. G. Malliaras , Nat. Rev. Mater. 2018, 3, 17086.

[323] A. M. Pappa , H. Y. Liu , W. Traberg‐Christensen , Q. Thiburce , A. Savva , A. Pavia , A. Salleo , S. Daniel , R. M. Owens , ACS Nano 2020, 14, 12538. 32469490 10.1021/acsnano.0c01330

[324] S. M. Kim , C. H. Kim , Y. Kim , N. Kim , W. J. Lee , E. H. Lee , D. Kim , S. Park , K. Lee , J. Rivnay , M. H. Yoon , Nat. Commun. 2018, 9, 3858. 30242224 10.1038/s41467-018-06084-6 PMC6155079

[325] P. Zhang , M. Xia , F. Zhuge , Y. Zhou , Z. Wang , B. Dong , Y. Fu , K. Yang , Y. Li , Y. He , R. H. Scheicher , X. S. Miao , Nano Lett. 2019, 19, 4279. 31150262 10.1021/acs.nanolett.9b00525

[326] Y. Zou , K. Xiao , Q. Qin , J. W. Shi , T. Heil , Y. Markushyna , L. Jiang , M. Antonietti , A. Savateev , ACS Nano 2021, 15, 6551. 33822587 10.1021/acsnano.0c09661 PMC8155341

[327] X. Zhang , B. Song , L. Jiang , CCS Chem. 2021, 3, 1258.

[328] Y. W. Hao , S. Pang , X. Q. Zhang , L. Jiang , Chem. Sci. 2020, 11, 10035. 34094265 10.1039/d0sc03574b PMC8162446

[329] L. Fu , Y. Wang , J. Jiang , B. Lu , J. Zhai , ACS Appl. Mater. Interfaces 2021, 13, 35197. 34266231 10.1021/acsami.1c10183

[330] M. Y. Wu , Z. Q. Li , G. L. Zhu , Z. Q. Wu , X. L. Ding , L. Q. Huang , R. J. Mo , X. H. Xia , ACS Appl. Mater. Interfaces 2021, 13, 32479. 34191482 10.1021/acsami.1c06535

[331] R. J. Ye , C. L. Ren , J. Shen , N. Li , F. Chen , A. Roy , H. Q. Zeng , J. Am. Chem. Soc. 2019, 141, 9788. 31184884 10.1021/jacs.9b04096

[332] C. L. Ren , F. Chen , R. J. Ye , Y. S. Ong , H. F. Lu , S. S. Lee , J. Y. Ying , H. Q. Zeng , Angew. Chem. Int. Ed. 2019, 58, 8034. 10.1002/anie.201901833 30983075

[333] A. Credi , Angew. Chem. Int. Ed. 2019, 58, 4108.

[334] Y. Chen , D. Su , Y. Chen , Z. Zhu , W. Li , Cell Rep. Phys. Sci. 2021, 10.1016/j.xcrp.2021.100602

[335] Y. Feng , H. Dai , J. Chen , X. Kong , J. Yang , L. Jiang , J. Mater Chem A 2019, 7, 20182.

[336] M. Jia , X. Kong , L. Wang , Y. Zhang , D. Quan , L. Ding , D. Lu , L. Jiang , W. Guo , Small 2019, 16, 1905557. 10.1002/smll.201905557 31805218

[337] Y. B. Zhang , F. Y. Li , X. O. Kong , T. Y. Xue , D. Liu , P. Jia , L. L. Wang , L. P. Ding , H. L. Dong , D. N. Lu , L. Jiang , W. Guo , Adv. Funct. Mater. 2019, 30, 1907549.

[338] P. P. Zhang , J. Li , L. X. Lv , Y. Zhao , L. T. Qu , ACS Nano 2017, 11, 5087. 28423271 10.1021/acsnano.7b01965

[339] Y. Chen , J. Meng , Z. Gu , X. Wan , L. Jiang , S. Wang , Adv. Funct. Mater. 2019, 30, 1905287.

[340] S. Li , Z. Fan , G. Wu , Y. Shao , Z. Xia , C. Wei , F. Shen , X. Tong , J. Yu , K. Chen , M. Wang , Y. Zhao , Z. Luo , M. Jian , J. Sun , R. B. Kaner , Y. Shao , ACS Nano 2021, 15, 7821. 33834770 10.1021/acsnano.1c02271

